# N^6^‐methyladenosine and RNA secondary structure affect transcript stability and protein abundance during systemic salt stress in Arabidopsis

**DOI:** 10.1002/pld3.239

**Published:** 2020-07-24

**Authors:** Marianne C. Kramer, Kevin A. Janssen, Kyle Palos, Andrew D. L. Nelson, Lee E. Vandivier, Benjamin A. Garcia, Eric Lyons, Mark A. Beilstein, Brian D. Gregory

**Affiliations:** ^1^ Department of Biology University of Pennsylvania Philadelphia PA USA; ^2^ Cell and Molecular Biology Graduate Group Perelman School of Medicine University of Pennsylvania Philadelphia PA USA; ^3^ Department of Biochemistry and Biophysics Perelman School of Medicine University of Pennsylvania Philadelphia PA USA; ^4^ Epigenetics Institute Perelman School of Medicine University of Pennsylvania Philadelphia PA USA; ^5^ Biochemistry and Molecular Biophysics Graduate Group University of Pennsylvania PA USA; ^6^ School of Plant Sciences University of Arizona Tucson AZ USA; ^7^ Boyce Thompson Institute Cornell University Ithaca NY USA; ^8^ CyVerse University of Arizona Tucson AZ USA

**Keywords:** non‐coding RNAs, post‐transcriptional regulation, RNA covalent modifications, RNA processing, RNA stability, RNA‐binding proteins

## Abstract

After transcription, a messenger RNA (mRNA) is further post‐transcriptionally regulated by several features including RNA secondary structure and covalent RNA modifications (specifically N^6^‐methyladenosine, m^6^A). Both RNA secondary structure and m^6^A have been demonstrated to regulate mRNA stability and translation and have been independently linked to plant responses to soil salinity levels. However, the effect of m^6^A on regulating RNA secondary structure and the combinatorial interplay between these two RNA features during salt stress response has yet to be studied. Here, we globally identify RNA‐protein interactions and RNA secondary structure during systemic salt stress. This analysis reveals that RNA secondary structure changes significantly during salt stress, and that it is independent of global changes in RNA‐protein interactions. Conversely, we find that m^6^A is anti‐correlated with RNA secondary structure in a condition‐dependent manner, with salt‐specific m^6^A correlated with a decrease in mRNA secondary structure during salt stress. Taken together, we suggest that salt‐specific m^6^A deposition and the associated loss of RNA secondary structure results in increases in mRNA stability for transcripts encoding abiotic stress response proteins and ultimately increases in protein levels from these stabilized transcripts. In total, our comprehensive analyses reveal important post‐transcriptional regulatory mechanisms involved in plant long‐term salt stress response and adaptation.

**Abbreviations**
CDS: coding sequence
dsRNA: double‐stranded RNA
dsRNase: double‐stranded ribonuclease
m6A: N6‐methyladenosine
nt: nucleotide
PIP‐seq: protein interaction profile sequencing
PPS: protein protected site
RBP: RNA binding protein
ssRNA: single‐stranded RNA
ssRNase: single‐stranded ribonuclease
UTR: untranslated region

## 1 INTRODUCTION

Similar to proteins, RNAs must fold into specific intramolecular conformations to function properly. This notion is emphasized by the known importance of RNA folding, also known as RNA secondary structure, on the function of several classes of noncoding RNAs (ncRNAs; reviewed in Vandivier, Anderson, Foley, & Gregory, 2016). Traditional examples include housekeeping ncRNAs such as ribosomal RNAs (rRNAs) and transfer RNAs (tRNAs), where the specific conformations that they fold into allow for proper interaction with proteins and formation of functional ribonucleoprotein complexes (e.g. ribosomes; Brimacombe & Stiege, 1985; Petrov et al., 2014), and enable amino acid addition to growing polypeptide chains respectively (Kim et al., 1974; Robertus et al., 1974). Likewise, long noncoding RNAs (lncRNAs) adopt specific structural patterns to interact with regulatory proteins and affect their ultimate function (Guttman & Rinn, 2012; Tsai et al., 2010). LncRNAs are generally not well‐conserved at the sequence level, thus it is hypothesized that their secondary structure is crucial for function and that it may be the conserved feature of this class of RNAs (Zampetaki, Albrecht, & Steinhofel, 2018).

Recent studies have demonstrated that secondary structure is equally important for messenger RNA (mRNA) regulation, particularly in plants (Foley, Kramer, & Gregory, 2017; Tack, Su, Yu, Bevilacqua, & Assmann, 2020; Yang, Yang, Deng, & Ding, 2018). mRNA secondary structure is thought to form co‐transcriptionally and undergo conformational changes throughout the lifecycle of a mRNA as it gets modified and processed in the nucleus. Ultimately, most mRNAs are exported into the cytoplasm, where secondary structure can help regulate ribosomal recruitment by allowing specific regions of a mRNA to be accessible to various proteins to initiate translation. It can also affect translation efficiency and mRNA stability, as higher intramolecular base pairing can slow down ribosome progress along the transcript (Kozak, 1988; Svitkin et al., 2001) or increase transcript stability (Beaudoin et al., 2018; Mauger et al., 2019; Suay, Salvador, Abesha, & Klein, 2005), respectively. Thus, RNA secondary structure can regulate many steps in the lifecycle of a mRNA molecule from transcription to translation and ultimately degradation (Beaudoin et al., 2018; Goodarzi et al., 2012). In particular, the initial secondary structures formed in the nucleus are of great importance as these structures help dictate further mRNA processing and export, and ultimately its fate.

The mechanisms of RNA secondary structure formation are complex and involve several post‐transcriptional regulatory steps. An important force driving RNA secondary structure is the modification and editing of RNA nucleotides. One RNA modification in particular, N^6^‐methyladensine (m^6^A), is the most prevalent internal mRNA modification identified in eukaryotes (reviewed in Kramer, Anderson, & Gregory, 2018) and has been shown to affect RNA secondary structure by weakening intramolecular base pairing by conformational switching (Liu et al., 2015; Spitale et al., 2015; Sun et al., 2019). Moreover, m^6^A can regulate nearly every stage of post‐transcriptional gene regulation, including mRNA stability (Anderson et al., 2018; Wang, Lu, et al., 2014; Wang, Li, et al., 2014), localization (Wang, Lu, et al., 2014), and translation (Mao et al., 2019; Meyer et al., 2015; Wang et al., 2015). While the effect of m^6^A on RNA secondary structure is well‐characterized, this relationship is unstudied in plants. Furthermore, although the independent roles of m^6^A and RNA secondary structure during mRNA processing is being increasingly studied, the direct role of m^6^A‐mediated changes in RNA secondary structure on mRNA processing has not been directly tested.

In addition to the effect of m^6^A on secondary structure, an important driving force dictating RNA secondary structure is the interaction between RNA and RNA binding proteins (RBPs; Vandivier et al., 2016). All RNAs are constantly bound by a varying cohort of RBPs that regulate every step in the life of a RNA, and can function as chaperones to guide RNA folding (Foley, Kramer, et al., 2017). RBP‐RNA interactions occur in highly sequence‐ and structure‐specific contexts, leading to two nonmutually exclusive ideas that (1) the identity of RBPs bound to a specific RNA can dictate whether a region is single‐ or double‐stranded and (2) that the single‐ or double‐stranded inherent nature of a RNA permits certain RBPs to bind. In fact, previous machine learning studies found that RNA secondary structure is an important predictor of RBP binding sites (Sun et al., 2019).

Recently, researchers identified the RNA binding proteome in the model plant *Arabidopsis thaliana* (hereafter Arabidopsis) by isolating polyadenylated RNAs and performing mass spectrometry to identify all proteins bound to polyadenylated RNAs (Marondedze, Thomas, Serrano, Lilley, & Gehring, 2016; Reichel et al., 2016). Proteins that were previously unidentified as RNA binding were enriched for the gene ontology (GO) term “response to stress”, in particular “response to osmotic stress” and “response to salt stress” (Marondedze et al., 2016). Additionally, a class of nonspecific RBPs known as RNA chaperones function to provide assistance in the correct folding of RNA molecules during post‐transcriptional regulation. Several of these RNA chaperone proteins are known to function in abiotic stress response as well, including response to cold and salt stress (Kang, Park, & Kwak, 2013; Kim et al., 2013). While there is evidence that RBPs are important regulators of salt stress response in Arabidopsis, where RBPs bind to RNAs on a transcriptome‐wide scale during salt stress response has not been studied.

Salt stress is a major factor limiting crop yield worldwide. Modern agricultural practices, poor irrigation, and lack of drainage increase soil salinity globally. In fact, it is predicted that one‐third of all irrigated land is affected by increased soil salinity and that by the year 2050 over 50% of the suitable land will be affected (Jamil, Riaz, Ashraf, & Foolad, 2011). Excess salt in the soil makes it more difficult for the plant to absorb water from its surroundings, causing plants to not only experience stress in response to the added NaCl but also drought/osmotic stress (Munns & Tester, 2008). Over time, increases in soil salinity leads to decreased plant growth and development (Yamaguchi & Blumwald, 2005). During exposure to salt stress, plants undergo major transcriptomic reprogramming to respond properly (Anderson et al., 2018; Ding, Cui, et al., 2014; Kreps et al., 2002). Given the role of RBPs and RNA chaperone proteins in regulating response to salt stress, understanding the role of RBPs and post‐transcriptional regulation in systemic salt stress in hopes to better engineer crops to withstand the imminent increasing soil salinization should be a major focus of future research efforts.

To begin to address this knowledge gap, we used protein interaction profile sequencing (PIP‐seq) to simultaneously identify protein‐bound regions on a transcriptome‐wide scale and examine global patterns of RNA secondary structure during systemic salt stress response in Arabidopsis. This analysis shows that mRNA secondary structure significantly changes during salt stress response. Additionally, we show that the presence of m^6^A is anti‐correlated with mRNA secondary structure, suggesting that the presence of this modification alleviates intramolecular base pairing during salt stress response through direct or indirect mechanisms. We further demonstrate that transcripts that gain m^6^A in a salt‐dependent manner and are stabilized during salt stress are transcripts encoding proteins involved in stress response. These transcripts show major changes in RNA secondary structure and increased protein abundance during salt stress. Taken together, these data suggest a mechanism for engineering a system wherein m^6^A is deposited on transcripts encoding stress response proteins only when exposed to salt stress, resulting in increased mRNA stability. The m^6^A‐mediated increase in mRNA stability is associated with a subsequent decrease in mRNA secondary structure and ultimately results in increased protein abundance of proteins needed for proper response to systemic salt stress.

## 2 METHODS

### 2.1 Plant materials

All plants were grown in controlled chambers with a cycle of 16 hr light and 8 hr of dark at 22°C. All experiments were performed using UBQ10:NTF/ACT2p:BirA Columbia‐0 ecotype of *Arabidopsis thaliana* (Deal & Henikoff, 2010). Salt‐stress experiments for PIP‐seq, mRNA‐seq, GMUCT, m^6^A‐seq, and mass spectrometry were carried out as previously described (Anderson et al., 2018). Salt concentrations were optimized based on the decrease in fresh weight determined previously (Monihan et al., 2020; Monihan, Ryu, Magness, & Schumaker, 2019).

### 2.2 Crosslinking and INTACT

Before nuclei purification, control‐ and salt‐treated rosette leaves of UBQ10:NTF/ACT2p:BirA Columbia‐0 ecotype were crosslinked in a 1% (vol/vol) formaldehyde solution in 1X PBS under vacuum for 10 min followed by a 5 min quench in 125 mM glycine under vacuum. Crosslinked tissue was then frozen in liquid nitrogen until INTACT purification, as previously described (Deal & Henikoff, 2010; Gosai et al., 2015).

Briefly, 3 grams of rosette leaves from control‐ and salt‐treated conditions were pulverized in liquid nitrogen then resuspended in 30 ml ice‐cold nuclei purification buffer (NPB; 20 mM MOPS (pH = 7), 40 mM NaCl, 90 mM KCl, 2 mM EDTA, 0.5 mM EGTA, 0.5mM spermidine, 0.2 mM spermine) with RNase and protease inhibitors (0.5 µl/ml RNase OUT; Invitrogen; complete protease inhibitor; Roche). The solution was then passed over a 70 µM Nylon mesh filter and incubated on ice for at least 10 min. Samples were then centrifuged at 1,200 rcf for 10 min at 4°C and pelleted nuclei were gently resuspended in 3 ml (1 ml per gram of tissue) NPB plus inhibitors and separated into 3–1.7 ml tubes containing 1 ml each. Following resuspension, 25 µl streptavidin coated M‐280 Dynabeads (Invitrogen) per gram of tissue were washed twice with NPB before being ultimately resuspended in NPB and added to samples. Samples were incubated for at least 30 min at 4°C with end‐over‐end rotation, after which they were transferred to 15 ml conical tubes containing 12 ml NPB supplemented with 0.1% (vol/vol) Tween‐20 (NPBt). Samples were washed four times for 2 min each at 4°C with end‐over‐end rotation. After the last wash, the beads were resuspended in 1 ml NPBt and transferred to 1.7 ml tubes and washed two additional times with 1 ml NPBt for 2 min at 4°C. 1/10th of the final sample was removed, stained with DAPI, and visualized by fluorescence microscopy to ensure purity of nuclear samples and count the number of nuclei extracted. The final samples were resuspended in 20 µl NPB and samples from the same tissue were recombined before being frozen in liquid nitrogen and stored at −80°C until processing.

### 2.3 Western blotting

To validate nuclear purity, western blots from control‐ and salt‐treated INTACT purified nuclei or whole leaf lysate was performed using anti‐PEPC (1:1,000; AS09 458; Agrisera), anti‐CNX1/2 (1:500; AS12 2365; Agrisera) and anti‐H3 (1:1,000; ab1791; Abcam) antibodies. Briefly, nuclear lysates were separated on a 4%–12% SDS NuPAGE gel (Invitrogen) in 1X MES for 90 min at 100 V. Samples were then transferred to PVDF at 200 mA for 2 hr at 4°C. The membrane was then blocked in 5% milk in TBS with 0.1% (vol/vol) Tween‐20 (TBST) at room temperature for 2 hr, before blotted with the primary antibodies in 5% milk in TBST overnight at 4°C. Excess primary antibody was washed by three 10 min washes in TBST. The secondary antibody (goat anti‐rabbit IgG H&L; PhytoAB) was diluted 1:5,000 (CNX1/2) or 1:10,000 (PEPC, H3) in TBST and blotted for 1 hr at room temperature. Excess antibody was washed by three 10 min washes with TBST. The membrane was then removed from liquid and ECL Prime Western Blotting Detection Reagent (GE Healthcare) was applied to the membrane for five minutes. Images were taken incrementally every 10 s until saturation.

To examine abundance of P5CS1, a western blot of control‐ and salt‐treated rosette leaves was performed using anti‐P5CS1 (1:1,000; PhytoAB) and anti‐ACTIN (1:1,000; PhytoAB). ~2 grams of rosettes from control‐ and salt‐treated tissue were crushed in liquid nitrogen before being added to 5 ml RIP buffer (150 mM NaCl, 20 mM Tris‐HCl (pH = 8), 1 mM EDTA, 5 mM MgCl_2_, 0.5% NP‐40 with complete protease inhibitor; Roche) in a 50 ml conical tube, mixed well and incubated on ice for at least 30 min. Tissue was further broken up using an Omni Tissue Homogenizer (TH115; Omni International Inc.) twice for 30 s each on medium speed using Omni Soft Tissue probes (30750; Omni International Inc.). Samples were then centrifuged for 15 min at 8,000 rcf at 4°C. The supernatant was transferred to a new 15 ml conical and centrifuged for 15 min at 8,000 rcf at 4°C. Supernatant was transferred again and the concentration of protein quantified by Bradford assay. A western blot with 20 µg of protein from two biological replicates of control‐ and salt‐treated samples was then conducted as mentioned above. Secondary antibody (goat anti‐rabbit IgG H&L; PhytoAB) was diluted 1:10,000 in TBST. Quantification of bands was done as previously described (Davarinejad, 2015).

### 2.4 PIP‐seq library preparation

PIP‐seq libraries were constructed as previously described (Foley & Gregory, 2016; Kramer & Gregory, 2019). To summarize briefly, INTACT purified nuclei from 3 g of tissue per replicate were lysed and separated into footprinting and structure‐only samples. The footprinting samples were then treated with either dsRNase (RNaseV1; purified, tested, and validated in the Gregory lab with Protein Labs; ds‐P) or ssRNase (RNaseONE; Promega; ss‐P) before protein digestion by proteinase K and reversal of crosslinks. In the structure‐only samples, proteins were first digested with proteinase K before treatment with either dsRNase (P‐ds) or ssRNase (P‐ss). Each sample resulted in four libraries: two footprinting libraries (ss‐P, ds‐P) and two structure‐only libraries (P‐ss, P‐ds). All libraries were sequenced on an Illumina HiSeq2000 using the standard protocol for 50 base pair single read sequencing.

### 2.5 Read processing, and alignment

Read processing and alignment was done as previously described (Foley, Gosai, et al., 2017; Gosai et al., 2015; Shan, Anderson, & Gregory, 2019; Silverman et al., 2014). To accurately identify PPSs without sequencing depth biases between the structure‐only control samples and the footprinting samples, the fastq files from the Illumina sequencing for each replicate of footprinting and structure‐only libraries from the same condition (control or salt) and RNase treatment (dsRNase or ssRNase) were paired (i.e. control rep1 dsRNase in the presence of protein [P‐ds] or absence of protein [ds‐P]) and the larger of the libraries was then randomly reduced to contain the same amount of reads as the smaller library. After sequencing, all PIP‐seq libraries were trimmed to remove 3’ sequencing adapters using cutadapt (version 1.9.1 with parameters ‐e 0.06 ‐O 6 ‐m14). The resulting trimmed and untrimmed reads were collapsed to unique reads and first mapped to rRNA, tRNA and repetitive regions of the *Arabidopsis thaliana* TAIR10 genome using TopHat (version 2.0.10 with parameters ‐‐library‐type fr‐secondstrand ‐‐read‐mismatches 2 ‐‐read‐edit‐dist 2 ‐‐max‐multihits 10 ‐‐b2‐very‐sensitive ‐‐transcriptome‐max‐hits 10 ‐‐no‐coverage‐search ‐‐no‐novel‐juncs). The remaining reads were then mapped to the TAIR10 genome. PCR duplicates were collapsed to single reads for all subsequent analyses.

### 2.6 PIP‐seq library reproducibility

Read coverage for all PIP‐seq libraries was calculated in 1,000 nt bins with a 100 nt sliding window (i.e. 0–1,000, 100–1,100…) using coverageBed with ‐s to define strandedness. The number of reads in each bin were than normalized by the total number of reads in each library per million and replicates were plotted against each other. Pearson's correlation was performed to determine reproducibility.

Additionally, read coverage for all PIP‐seq libraries was calculated in 1,000 nt tiled bins (0–1,000, 1,000–2,000…) using coverageBed with ‐s to define strandedness. DESeq2 (Love, Huber, & Anders, 2014) was then used to cluster libraries together by coverage in 1,000 nt tiled bins across the genome.

### 2.7 Identification of PPSs

PPSs were identified using a modified version of the CSAR software package, as previously described (Foley, Gosai, et al., 2017; Gosai et al., 2015; Shan et al., 2019; Silverman et al., 2014). Briefly, read coverage was calculated at each nucleotide in the genome and a Poisson test was used to determine an enrichment score for footprint compared to structure‐only samples. PPSs were then called with a false discovery rate of 5% as previously described (Foley, Gosai, et al., 2017; Gosai et al., 2015; Shan et al., 2019; Silverman et al., 2014). PPSs within each biological replicate that overlapped by at least 1 nucleotide were merged to generate a single PPS. High‐confidence PPSs were identified by intersectBed, with PPSs sharing at least 1 nucleotide in both biological replicates counted as high‐confidence. Control‐specific and salt‐specific high‐confidence PPSs were identified as PPSs that are found in both replicates of either control‐ or salt‐treated tissue and absent from both biological replicates of salt‐ or control‐treated tissue respectively.

### 2.8 Functional analysis of PPSs

Annotation of PPS location in mRNAs was done ‘greedily’ using the TAIR10 genome annotations, such that all functional annotations that overlapped with a given PPS were counted equally. Annotation of PPS location in ncRNAs was done similarly but with the Araport11 ncRNA annotation. Conservation was scored using PhastCons scored from six flowering plants generated previously (Li, Zheng, Vandivier, et al., 2012) and average PhastCons scores across a PPS was compared to equal sized regions flanking the PPS to the 5’ and 3’ end. PPS enrichment was calculated by comparing the average number of nucleotides in each PPS to the number of nucleotides in each genic region across the Arabidopsis transcriptome.

### 2.9 RBP density profiles

#### 2.9.1 mRNAs

**FIGURE 1:**
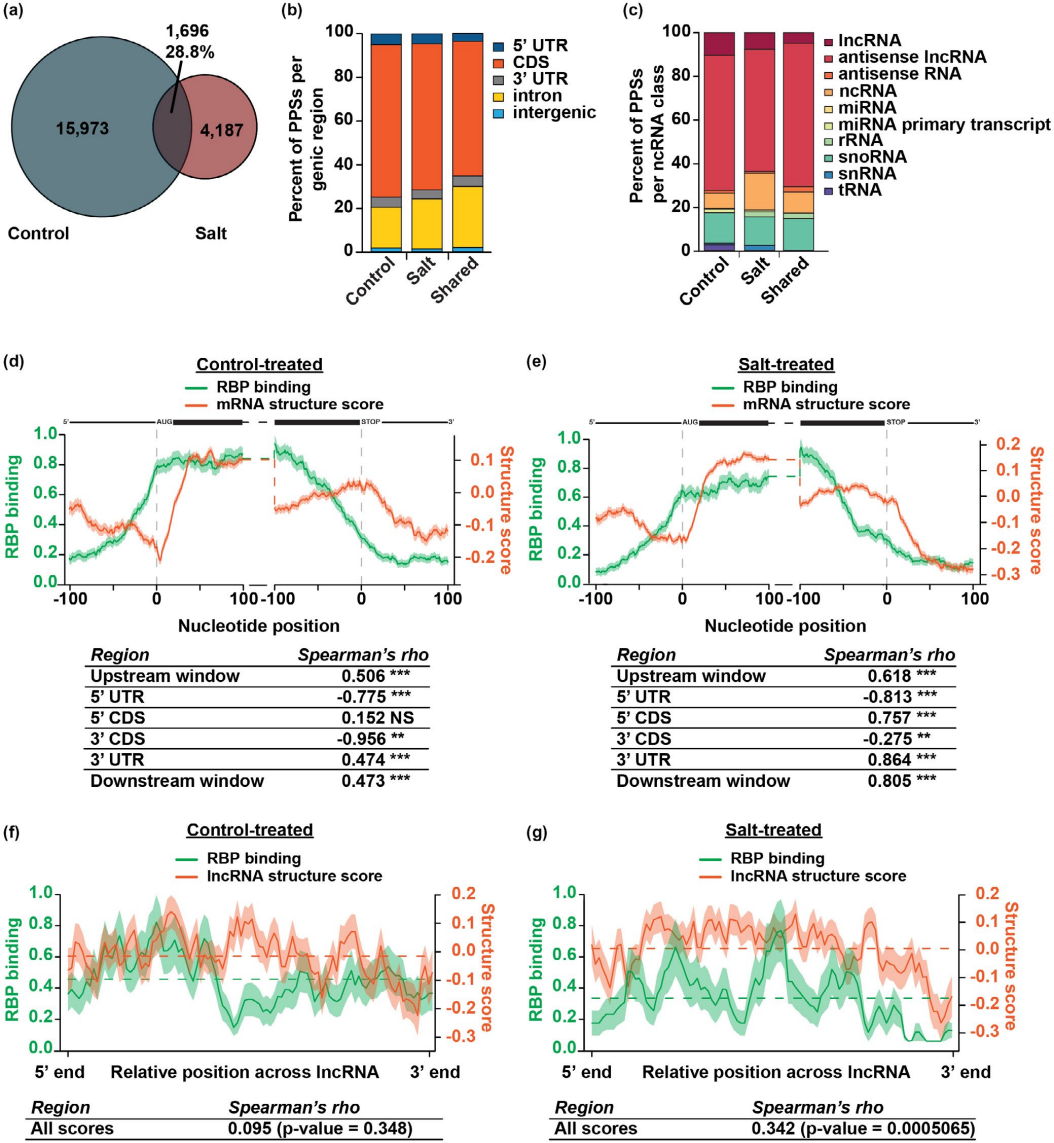
RNA secondary structure and RBP binding are correlated in 4‐week‐old rosette leaves. (a) Overlap between high‐confidence PPSs identified in both replicates of either control‐ (blue) or salt‐treated (red) tissue. The intersection indicates PPSs that overlap by at least one nucleotide. See Data Sets S1–S5. (b) Distribution of high‐confidence control‐specific, salt‐specific, and shared PPSs identified in each genic region within protein‐coding mRNAs. (c) Distribution of high‐confidence control‐specific, salt‐specific, and shared PPSs identified in various types of noncoding RNAs. (d,e) Average RBP binding (green line) and structure score (orange line) at each nucleotide ±100 nt of the annotated start and stop codon in nuclear mRNAs in control‐treated (d) or salt‐treated (e) tissue. The tables represent Spearman's rho correlations between RBP binding and structure score across the entire upstream window (±100 nt of the start codon), 5’ UTR, 5’ CDS, 3’ CDS, 3’ UTR, and downstream window (±100 nt of the stop codon) across all plotted transcripts. Shading around the line indicates the *SEM* across all plotted transcripts. High‐confidence PPSs identified in both replicates of control‐treated (*N* = 17,669) or salt‐treated (*N* = 5,883) tissue was used to calculate RBP binding. *N* = 14,461 mRNAs. *, **, and ***p < .05, .01, and .001, respectively, Spearman's asymptotic t approximation. mRNA diagrams above plots are not to scale. (f,g) Average RBP binding (green line) and structure score (orange line) across all binned, spliced lncRNAs (lncRNA, antisense lncRNAs, antisense RNA, ncRNA) in control‐treated (f) or salt‐treated (g) tissue. The tables represent Spearman's rho correlations between RBP binding and structure score across the entire binned window of the lncRNAs. Dashed lines indicate the average RBP binding (green) or structure score (orange) across the entire binned transcript. Shading around the line indicates the *SEM* across all plotted lncRNAs. High‐confidence PPSs identified in both replicates of control‐treated (*N* = 17,669) or salt‐treated (*N* = 5,883) tissue was used to calculate RBP binding. *N* = 906 lncRNAs. See Data Set S6. *p*‐values are as denoted; Spearman's asymptotic t approximation.

**FIGURE 2:**
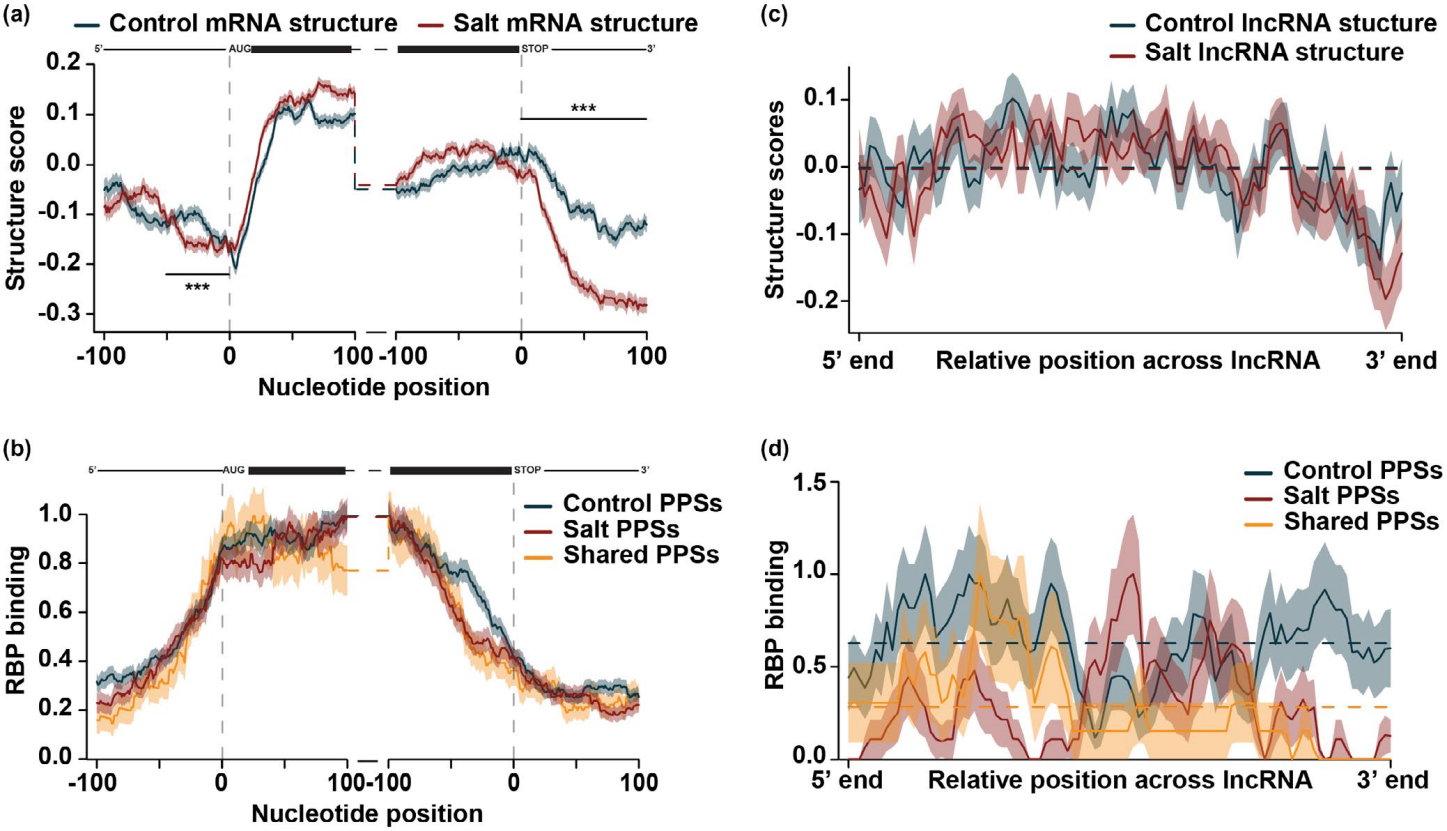
Nuclear RNA secondary structure significantly changes during salt stress response. (a,b) Average structure score (a) and RBP binding (b) in the ± 100 nt of the annotated start and stop codon in nuclear protein‐coding mRNAs expressed in both control‐treated (blue line) and salt‐treated (red line) tissue. High‐confidence PPSs were divided into those that were expressed exclusively in control‐treated tissue (blue line), salt‐treated tissue (red line) or common to both treatments (yellow line). See Data Sets S3–S5. Shading around the line indicates the *SEM* across all plotted transcripts. *N* = 14,461 mRNAs. ***p < .001, Wilcoxon test. mRNA diagrams above plots are not to scale. Grey shading is to highlight the 50 nt upstream of the start codon. (c,d) Average structure score (c) and RBP binding (d) across binned, spliced all lncRNAs (lncRNA, antisense lncRNAs, antisense RNA, ncRNA) expressed in both control‐treated (blue line) or salt‐treated (red line) tissue. High‐confidence PPSs were divided into those that were expressed exclusively in control‐treated tissue (blue line), salt‐treated tissue (red line), or common to both treatments (yellow line). See Data Sets S3–S5. Shading around the line indicates the *SEM* across all plotted transcripts. *N* = 906 lncRNAs.

RBP density was calculated by assigning a value of 1 or 0 at each nucleotide with a value of 1 indicating a PPS is found at that nucleotide and a 0 indicating no PPS is found. Only mRNAs with a minimum of 50 reads in all libraries across the entire transcript in both conditions, a ≥ 45 nt 5’ UTR, and ≥140 nt 3’ UTR were considered. RBP density was calculated for (1) all PPSs identified in each replicate separately (Figure S3c,d), (2) high‐confidence PPSs identified in both biological replicates (Figure 1f–g and Figure S7e,f), and (3) high‐confidence control‐specific, salt‐specific, and shared PPSs (Figure 2b,d and Figure S9b,c).

To generate profiles, introns were removed and the RBP density at each nucleotide was averaged for all transcripts that passed the above criteria and was plotted such that the highest bound region was normalized to a density of 1.0 in each window examined (i.e. the 100 nt window around the start codon are normalized together). Shading around the line represents the *SEM* at each nucleotide.

#### 2.9.2 lncRNAs

The Araport11 annotation of “lncRNAs”, “antisense lncRNAs”, “antisense RNA”, and “ncRNA” were merged together for all lncRNA analyses (Data Set S6). Only lncRNAs with a minimum coverage of five reads across the entire transcript in both conditions were considered. To generate profiles, introns were spliced out and each lncRNA was divided into 100 equal sized bins. RBP density was plotted such that the highest bound region within the binned lncRNA was normalized to a density of 1.0.

#### 2.9.3 m^6^A peaks

RBP binding for each m^6^A peak as well as equal sized regions flanking were extracted before being divided into equal sized bins. Average RBP binding was plotted for each bin with shading around the line representing the *SEM* at each nucleotide and normalized such that the highest bound regions across the flanks and m^6^A peaks were normalized to a score of 1.0.

### 2.10 m^6^A density profile analysis

m^6^A peaks were converted to a score at each nucleotide, with 1 indicating a m^6^A peak is found at that nucleotide and a 0 indicating no m^6^A peak found. The average m^6^A density was then averaged for all genes that passed the above expression criteria. m^6^A density was plotted such that the highest bound region was normalized to a density of 1.0 in each window examined (i.e. the 100 nt window around the start codon are normalized together). Shading around the line represents the *SEM* at each nucleotide.

### 2.11 Calculating structure score—normalizing by total structure

Structure score was calculated using the structure‐only samples as previously described (Foley, Gosai, et al., 2017; Gosai et al., 2015; Shan et al., 2019; Silverman et al., 2014). Briefly, for every base in our set of detectable transcripts, we calculated the ratio of the amount of coverage in the dsRNA‐seq and ssRNA‐seq libraries. For every value of dsRNA‐seq (*n_ds_*) and ssRNA‐seq (*n_ss_*) of a given base I, the structure score is calculated as follows:

$$\begin{array}{c} S_{i}=g\log\left(ds_{i}\right)‐g\log\left(ss_{i}\right)\\ \;=\log_{2}\left(ds_{i}+\sqrt{1+ds_{i}^{2}}\right)‐\log_{2}\left(ss_{i}+\sqrt{1+ss_{i}^{2}}\right). \end{array}$$

$$ds_{i}=n_{\mathrm{ds}}\frac{\max\left(L_{\mathrm{ds}},L_{\mathrm{ss}}\right)}{L_{\mathrm{ds}}},\;ss_{i}=n_{\mathrm{ss}}\frac{\max\left(L_{\mathrm{ds}},L_{\mathrm{ss}}\right)}{L_{\mathrm{ss}}}$$

where *S_i_* is the structure score, *ds_i_* and *ss_i_* are the normalized read coverages and *L_ds_* and *L_ss_* are the total covered length by mapped dsRNA‐seq and ssRNA‐seq libraries respectively. We then calculated standardized structure scores to normalize by read coverage. Structure scores were calculated for each replicate individually as well as each sample with the replicates merged together.

Average structure score was calculated by averaging all standardized scores within the 5’ UTR, CDS, 3’ UTR, or the whole spliced transcripts in control‐ and salt‐treated tissue. Fold change was calculated by subtracting the log‐transformed standardized structure scores (described above) in control from salt. Transcripts with a fold‐change >0 were termed ‘greater structure in salt’, and transcripts with fold‐change <0 were termed ‘lower structure in salt.’

### 2.12 Structure score profiles

#### 2.12.1 mRNAs

The structure score for every nucleotide of detected mRNAs was calculated using all mapped and spliced reads. We only considered mRNAs with a minimum of 50 reads in all libraries across the entire transcript in both conditions, a ≥ 45 nt 5’ UTR, and ≥140 nt 3’ UTR. To generate profiles, introns were spliced out and each nucleotide in the spliced transcript was normalized by the average structure score across the entire spliced transcript. At the start and stop codons, the average structure score at each nucleotide was plotted with shading around the line representing the *SEM* at each nucleotide.

For plotting secondary structure across the entire transcript, the 5’ UTR, CDS, and 3’ UTR were each divided into 100 equally sized bins. We then plotted the Z‐score of the structure score for each nucleotide with respect to the graphed bin as previously described (Berkowitz et al., 2016), with shading around the line representing the *SEM* at each nucleotide. The Z‐score of the structure score for each replicate separately was calculated for each nucleotide with respect to the graphed window as previously described (Berkowitz et al., 2016).

#### 2.12.2 lncRNAs

The Araport11 annotation of “lncRNAs”, “antisense lncRNAs”, “antisense RNA”, and “ncRNA” were merged together for all lncRNA analyses (Data Set S6). Only lncRNAs with a minimum coverage of five reads across the entire transcript in both conditions were considered. The structure score for every nucleotide of detected lncRNAs was calculated using all mapped and spliced reads. To generate profiles, each nucleotide in the spliced transcript was normalized by the average structure score across the entire unspliced transcript and introns were spliced out. Each lncRNA was divided into 100 equal sized bins and the Z‐score of the structure score for each nucleotide with respect to the graphed bin as previously described (Berkowitz et al., 2016), with shading around the line representing the *SEM* at each nucleotide.

#### 2.12.3 m^6^A peaks

Structure scores for each m^6^A peak as well as equal sized regions flanking were extracted before being divided into equal sized bins. Average structure score was plotted for each bin with shading around the line representing the *SEM* at each nucleotide. We generated shuffled m^6^A sites by using the bedtools function shuffleBed. Parameter ‐i was used to shuffle m^6^A peaks into random sites, parameter ‐incl was used to constrain shuffling to regions within annotated genes, ‐chrom to keep shuffled peaks on the same chromosome as the m^6^A peak, and ‐noOverlapping does not allow shuffled peaks to overlap at all.

### 2.13 Comparison to Tack *et al*

Reactivity scores were downloaded from Tack *et al*. for control‐ and salt‐treated tissue. Only transcripts with reactivities in both control and salt in Tack *et al*. and both control‐ and salt‐treated tissue from PIP‐seq were used for comparison. Pearson correlation was calculated to examine correlation and significance. Plots were made using geom_hex in the ggplot2 package with 50 bins specified. Color of each bin indicates the number of transcripts that fall within that range.

### 2.14 *AT2G39800* (*P5CS1*) structure

Structure score for the m^6^A peaks identified in the 3’ UTR of *AT2G39800* were extracted along with equal sized regions flanking. RNAfold (http://rna.tbi.univie.ac.at/cgi‐bin/RNAWebSuite/RNAfold.cgi) was used for constrained RNA folding using PIP‐seq structure score data. Nucleotides with structure scores greater than 2.0 were constrained to be double‐stranded and nucleotides with structure score <−0.5 were constrained to be single‐stranded. All other nucleotides had no constraint enforced. The resulting dot‐bracket notation was transferred to the *forna* RNA secondary structure visualization tool (http://rna.tbi.univie.ac.at/forna/) and secondary structure model was generated without ‘circularize exterior loop’ enforced and the colors set to represent the structure scores for each nucleotide, with darker colors indicating higher structure score, thus higher probability of being double‐stranded. Structure score plots were generated by plotting the structure score for each nucleotide as well as the equal sized flanking regions.

### 2.15 Differential abundance analysis

Gene counts for each transcript were called using HTseq‐count on aligned mRNA‐seq reads using the parameters–format = bam–stranded = reverse–mode = intersection‐strict. Differentially abundant transcripts were called using the R package DESeq2 and the fold change and normalized read counts provided by this package were used for subsequent analyses, as previously described (Anderson et al., 2018).

### 2.16 mRNA stability

Proportion uncapped was calculated as described previously (Anderson et al., 2018). Transcripts that had a higher proportion uncapped in salt compared to control were termed ‘destabilized in salt’ while transcripts with lower proportion uncapped were termed ‘stabilized in salt’ (Data Set S9).

### 2.17 Protein lysate for MS

Three biological replicates of control‐ and salt‐treated rosette leaves were crushed in liquid nitrogen and transferred to 2 ml of 8 M urea and 100 mM ammonium bicarbonate with complete protease inhibitor (Roche; Basel, Switzerland) for further grinding. Lysate was then transferred to six 1.7 ml tubes with 300 µl each and sonicated for 30 min at 4°C 30 s on/2 min off. Samples were then spun at >20,000 rcf for 5 min at 4°C. The supernatant was transferred to a new tube and flash frozen in liquid nitrogen until used for mass spectrometry analysis.

### 2.18 Mass spectrometry

Samples were reduced by incubating 100 µl aliquots of 3 µg/µl protein in 10 mM dithiothreitol at 56°C for 30 min. Samples were cooled to room temperature and alkylated by adding 11 µl of 0.5 M iodoacetamide and incubating at room temperature in the dark for 40 min. The solutions were diluted to 500 µl in 50 mM Tris‐HCl (pH 8.3) and treated with 6 µl of 1 µg/µl trypsin. Tubes were placed on a rotator at 37°C and incubated overnight to digest proteins. To prepare peptides for mass spectrometry analysis, Stop and Go Extraction tip (Stagetip) were used on 10 µg aliquots as previously described (Rappsilber, Ishihama, & Mann, 2003). In brief, samples were loaded onto C18 resin in 0.1% trifluoroacetic acid (TFA), washed with 0.1% TFA, and eluted in 0.1% TFA in 60% acetonitrile (ACN). Samples were dried in a Savant SpeedVac and resuspended in 0.1% formic acid (FA) at 1 µg/µl.

A Thermo Easy NanoLC 1,000 was used to inject 1 µg of sample onto a column (75 µm × 15 cm) packed in‐house with C18 resin (Dr. Maisch, GMBH). Samples were loaded in buffer A (0.1% FA) and separated using a gradient of 2% buffer B (0.1% FA in ACN) to 30% buffer B over 90 min. Data dependent acquisition was performed on a Thermo Orbitrap Fusion mass spectrometer and data were processed in MaxQuant. iBAQ values were normalized to each run and only proteins with peptides identified in at least two biological replicates in control‐ and salt‐treated tissue were used for all analyses.

### 2.19 GO enrichment

GO enrichment analyses of transcripts that contain salt‐specific m^6^A and were either stabilized or destabilized during salt stress were performed using the DAVID online tool (Huang, Sherman, & Lempicki, 2009). All detectable transcripts with greater than 1 RPM in control‐ and salt‐treated tissue were used as a background.

## 3 RESULTS AND DISCUSSION

### 3.1 PIP‐seq identifies thousands of stress‐specific protein‐bound sites

Given the importance of RNA secondary structure on post‐transcriptional regulation and the role of RBPs during salt stress, we aimed to obtain a global view of RBP‐RNA interactions and RNA secondary structure in the nucleus during salt stress. To this end, we used the isolation of nuclei tagged in specific cell types (INTACT; Deal & Henikoff, 2010) system to isolate nuclear samples after a long‐term salt treatment that mimicked agriculturally relevant salt stress conditions. Briefly, we planted seeds of the Arabidopsis ecotype Columbia‐0 (Col‐0) that ubiquitously express a biotin ligase receptor peptide fusion protein that is targeted to the nuclear envelope (UBQ10:NTF/ACT2p:BirA Col‐0; Deal & Henikoff, 2010) and allowed the seeds to germinate and grow under standard conditions until the first true leaves were established, approximately 10 days post germination. At this time, we either continued with normal watering conditions or introduced the long‐term salt treatment, as previously described (Anderson et al., 2018). For systemic salt stress treatment, we slowly increased the concentration of NaCl in the watering solution, beginning with 50 mM NaCl and increasing to a final concentration of 150 mM NaCl in 50 mM increments every three days. We continued to water at 150 mM NaCl for 10 days before collecting the rosettes leaves and crosslinking RNA‐protein interactions with 1% formaldehyde (Figure S1a). After exposure to long‐term salt stress, the salt‐treated plants were smaller and darker in color as a result of the production of the stress pigment anthocyanin in salt stress compared to control, indicating that they are being stressed by the introduction of NaCl into the water (Figure S1b).

Using the INTACT system, we isolated nuclei enriched in the nuclear marker H3 but devoid of the cytoplasmic marker PEPC (Figure S1c). It was also noticed that there were detectable levels of the endoplasmic reticulum (ER) marker, CNX1/2, in the isolated nuclei, indicating that our sample contains a majority of nuclear RNAs as well as some RNAs associated with the ER. However, the isolated nuclei were free from cytoplasmic and cellular debris, as visualized by microscopy and DAPI staining (Figure S1d). With ~1.7–2 million nuclei per biological replicate (from a total of 3 grams of rosette tissue), we performed protein interaction profile sequencing (PIP‐seq), a technique developed to study RNA‐protein interactions and RNA secondary structure on a transcriptome‐wide scale (Foley, Gosai, et al., 2017; Gosai et al., 2015; Silverman et al., 2014). In PIP‐seq, the nuclei were lysed and divided into two groups termed the structure‐only and footprinting samples. The structure‐only samples were treated first with proteinase K to obtain a pool of RNA devoid of RBPs before being divided again and treated with structure specific ribonucleases (RNases) that digest single‐stranded RNA (ssRNA; ssRNase) or double‐stranded RNA (dsRNA; dsRNase). After RNA‐seq library preparation and sequencing, nuclear RNA secondary structure is predicted in control‐ and salt‐treated tissue by comparing the structure‐only samples treated with ssRNase to those treated with dsRNase (Foley & Gregory, 2016; Kramer & Gregory, 2019).

In parallel, the footprinting samples were used to identify RBP bound regions of RNA. The footprinting samples were first divided in half and treated with the structure‐specific RNases in the presence of proteins. This permits digestion of all accessible ssRNA or dsRNA, while regions that are bound by protein will be protected from digestion. Thus, after subsequent protein digestion, RNA‐seq library preparation, and sequencing, regions bound by protein are identified as sequences that are enriched in the footprinting sample compared to the structure‐only sample, which are defined as protein protected sites (PPSs; Foley & Gregory, 2016; Kramer & Gregory, 2019). The resulting PIP‐seq libraries (4 per sample; 2 structure‐only, 2 footprinting libraries) produced between 58–200 million raw reads per library. To determine reproducibility, we used a 100 nucleotide (nt) sliding window to generate 1,000 nt bins to calculate the correlation of nonredundant sequence read abundance between biological replicates of the footprinting and structure‐only libraries in control‐ and salt‐treated tissue. This revealed high correlations between biological replicates for all libraries (Pearson correlation R > 0.86), indicating the high reproducibility of these PIP‐seq libraries (Figure S2a–h). Similarly, a principle component analysis of nonredundant sequence read abundance in 1,000 nt tiled bins using DESeq2 (Love et al., 2014) revealed that libraries produced from the distinct RNase treatments clustered together. Within each RNase treatment, the conditions also primarily clustered together, further indicating the high‐quality and specificity of these nuclear PIP‐seq libraries (Figure S2i).

To identify PPSs in control‐ and salt‐treated tissue, a Poisson distribution model was used to identify enriched regions in the footprinting sample compared to the structure‐only libraries with a false discovery rate of 5%, as described previously (Foley, Gosai, et al., 2017; Gosai et al., 2015; Shan et al., 2019; Silverman et al., 2014). In total, we identified 45,826 and 52,384 PPSs in both biological replicates of control‐ and salt‐treated tissue, respectively, with 17,669 (~63%) and 5,883 (~23%) PPSs identified in both biological replicates in control‐ and salt‐treated tissue respectively (Figure S3a,b; Data Sets S1, S2). The low overlap between salt‐treated biological replicates is likely due to biological differences during the stress response. To ensure reproducibility of PPSs in control‐ and salt‐treated tissue, we calculated RBP binding density of all PPSs identified in each biological replicate by assigning each nucleotide a score of 1 or 0 based on whether or not a PPS was identified at that site, with 1 indicating that nucleotide is within a PPS and 0 indicating that nucleotide is not within a PPS. RBP binding was then plotted such that the highest region of occupancy is normalized to a density of 1.0. RBP binding for both biological replicates of both control‐ and salt‐treated tissue shared similar patterns of RBP binding and overall RBP binding densities, confirming the reproducibility of the identified PPSs (Figure S3c,d).

To guard against artifacts in our subsequent analyses, we focused on PPSs identified in both biological replicates, hereafter referred to as high‐confidence PPSs. Comparison of high‐confidence PPSs from control‐ and salt‐treated tissues found that 28.8% (1,696; shared PPSs; Data Set S3) of high‐confidence PPSs identified in salt‐treated tissue were also identified in control‐treated tissue, suggesting that these PPSs represent regions in the transcriptome that are constitutively bound by RBPs in 4‐week‐old plants (Figure 1a). Additionally, there were 15,973 PPSs exclusively found in control‐treated (high‐confidence control‐specific) and 4,187 PPSs exclusively found in salt‐treated tissue (high‐confidence salt‐specific; Figure 1a; Data Sets S4, S5), indicating that regions of the transcriptome are bound in a condition‐specific manner.

To examine the functional importance of the identified high‐confidence PPSs, we compared average PhastCons conservation scores from flowering plants (Li, Zheng, Vandivier, et al., 2012) for control‐specific, salt‐specific, and shared high‐confidence PPSs to average scores of equal sized regions flanking the PPSs. Since RBPs tend to bind in a sequence dependent manner, there is likely evolutionary pressure to retain the sequences of these sites. In accordance with this and as observed previously (Foley, Gosai, et al., 2017; Gosai et al., 2015; Silverman et al., 2014), PPSs in all three classes were significantly (*p* < 1 × 10^−10^, Kolmogorov‐Smirnov test) more conserved than regions within the same genomic regions flanking the PPS (Figure S4a). The majority (>96%) of high‐confidence PPSs identified were located within protein‐coding mRNAs (Figure S4b), particularly in the coding region (CDS; ~61%–70%) and introns (~19%–27%) of protein‐coding transcripts for all three classes of PPSs (Figure 1b).

To determine if the enrichment we observed was simply due to the fact that the CDS and introns constitute the majority of the transcriptome, we compared the number of bases bound by RBPs compared to the number of bases annotated as each feature (5’ UTR, CDS, 3’ UTR, intron) in the TAIR10 genome (Figure S4c). Similar to our previous studies (Foley, Gosai, et al., 2017; Gosai et al., 2015), all high‐confidence PPSs were enriched in the CDS and under‐represented in the untranslated regions (UTRs). Thus, the high protein binding in the CDS appears to be an inherent quality of nuclear mRNAs in Arabidopsis. This high protein binding in the CDS may be indicative of the importance to maintain and protect the CDS from external factors, aid in co‐transcriptional processes such as mRNA splicing, and ultimately help direct export into the cytoplasm, but additional studies are needed to test this hypothesis.

While the majority of PPSs were localized in protein‐coding genes, there was a distinct fraction that were located in noncoding RNAs (ncRNAs; Figure S4b). NcRNAs consist of several classes of RNAs that are broadly defined as RNAs that do not encode proteins. Using the Araport11 annotation of ncRNAs, the majority of PPSs found in ncRNAs were in long noncoding RNAs (lncRNAs), specifically “antisense lncRNAs” (Figure 1c). LncRNAs closely resemble protein‐coding transcripts, as they are similar in length (>200 nt), are usually polyadenylated, can be spliced, and have a 5’ cap, but differ in that they either lack or have an open reading frame of less than 100 amino acids. Given the high overlap in definition of the longer ncRNAs in the Araport11 annotation, we combined transcripts annotated as “lncRNAs”, “antisense lncRNAs”, “antisense RNA”, and “ncRNA” into a single group for all future analyses (Data Set S6). Aside from PPSs localized in lncRNAs, the next largest subset of PPSs was found to be in small nucleolar RNAs (snoRNAs; Figure 1c), which are known to be highly protein bound, nuclear‐retained small RNAs (60–200 nt long) that guide modification of nucleotides in rRNAs (Reichow, Hamma, Ferré‐D’Amaré, & Varani, 2007). Thus, PIP‐seq can identify RBP binding sites within ncRNAs known to be highly protein‐bound as well as identify condition‐specific, global RBP‐RNA interaction sites throughout the plant transcriptome. Identifying what proteins bind in a condition‐specific manner will be a subject for future studies.

### 3.2 Secondary structure and RBP binding show complex patterns in mRNAs and are positively correlated in 4‐week‐old rosette leaves

To examine the relationship between nuclear RBP binding and RNA secondary structure during salt stress, we calculated the density of high‐confidence PPSs and structure scores at each nucleotide (termed RBP binding and RNA secondary structure, respectively). Using the structure‐only samples, structure scores were calculated as a generalized log ratio of the reads in the dsRNA‐seq library (produced by the ssRNase) compared to the ssRNA‐seq library (produced by the dsRNase) at each nucleotide (Shan et al., 2019). The raw structure scores were then normalized to the average structure score of the entire spliced transcript, resulting in structure scores in which the positive or negative values indicate the likelihood of a nucleotide being double‐stranded (more structured) or single‐stranded (less structured) respectively. To ensure reproducibility of the calculated structure scores, structure scores for each biological replicate of control‐ and salt‐treated tissue were calculated separately. This revealed that the overall structure patterns and scores were significantly (Spearman's rho > 0.735; *p* < 2.2 × 10^–16^; asymptotic t approximation) similar between biological replicates in control‐ and salt‐treated tissue in the 200 nt surrounding the start and stop codon of nuclear mRNAs expressed in both tissues, further confirming the high reproducibility of the PIP‐seq experiments (Figure S5a,b). Thus, all further analyses were performed using structure scores calculated from merged biological replicates.

To compare the patterns of RNA secondary structure and RBP binding, we focused on the region 100 nt up‐ and downstream of the start and stop codon of nuclear mRNAs expressed in both control‐ and salt‐treated tissue, as these regions have important regulatory functions in mRNA fate. The highest RBP binding density of high‐confidence PPSs identified in control‐ and salt‐treated tissue was in the CDS of nuclear mRNAs with drastic increases and decreases over the start and stop codons, respectively (Figure 1d,e; green lines). This distribution is consistent with the observed PPS localization (Figure 1b) and enrichment (Figure S4c) as well as previous studies in the nuclei from 10‐day‐old whole seedlings and roots (Foley, Gosai, et al., 2017; Gosai et al., 2015), ultimately suggesting that nuclear mRNAs are bound predominantly in the CDS in Arabidopsis.

Similar to protein binding, RNA secondary structure scores were higher in the CDS compared to the 5’ UTR and 3’ UTR. This is contrary with previous findings in the nuclei from 10‐day‐old whole seedlings and roots (Foley, Gosai, et al., 2017; Gosai et al., 2015), suggesting that RNA secondary structure may be regulated in a tissue‐ and/or developmental time‐specific manner. These structural signatures of 4‐week‐old leaves as compared to young seedlings may represent an added layer of post‐transcriptional regulation to help dictate mRNA fate in a developmental time‐specific manner. In agreement with numerous studies of RNA secondary structure across multiple organisms (Ding, Tang, et al., 2014; Foley, Gosai, et al., 2017; Gosai et al., 2015; Li, Zheng, Ryvkin, et al., 2012; Li, Zheng, Vandivier, et al., 2012), there was a dip in RNA secondary structure directly over the start codon in both control‐ and salt‐treated tissue (Figure 1d,e; orange lines). Thus, the structural features surrounding the start codon is a consistent feature of the Arabidopsis nuclear and, more broadly, eukaryotic mRNA transcriptomes, but the patterns of secondary structure across mRNAs is regulated in a developmental and/or tissue‐specific manner.

Since RBP‐RNA interactions are highly dependent on RNA secondary structure and/or RBPs determine RNA secondary structure, we directly compared RBP binding and structure scores. Opposite to what was previously observed in nuclei from 10‐day‐old whole seedlings and roots (Foley, Gosai, et al., 2017; Gosai et al., 2015), there was an overall positive correlation around the start (upstream window; Spearman's rho = ~0.5–0.6; *p* < 2.2 × 10^–16^; asymptotic t approximation) and stop codon (downstream window; Spearman's rho = 0.4–0.8; *p* < 2.2 × 10^–16^; asymptotic t approximation) in both control‐ and salt‐treated 4‐week‐old Arabidopsis nuclei (Figure 1d,e). This further supports a model in which interactions between RBPs and RNA secondary structure are developmental and/or stress‐dependent. While there was an overall positive correlation in the 200 nt around the start and stop codons, a look closer at the 5’ UTR, and CDS identified a different trend, with significant anti‐correlation in the 5’ UTR (Spearman's rho <−0.775; *p* < 2.2 × 10^–16^; asymptotic t approximation) and within the 100 nt upstream of the stop codon (3’ CDS; Spearman's rho <−0.275; *p* < .01; asymptotic t approximation). Overall, the relationship between RNA secondary structure and RBP binding is highly dependent on the transcript region of inquiry and is regulated in a condition dependent manner.

As lncRNAs closely resemble protein‐coding mRNAs but lack protein‐coding capacity, we asked whether the relationship observed between RNA secondary structure and RBP binding is a specific feature of protein‐coding mRNAs. To do so, we took the entire length of annotated lncRNAs (Data Set S6) and divided each transcript into 100 equal sized bins and graphed the average structure score and RBP binding of each bin. Similar to mRNAs, there was a positive correlation between structure scores and RBP binding in control‐ (Spearman's rho = 0.095; *p* > .05; asymptotic t approximation) and salt‐treated plants (Spearman's rho = 0.342; *p* < .001; asymptotic t approximation; Figure 1f,g). Whereas there were distinct patterns of RNA structure and RBP binding at the start and stop codon of protein‐coding transcripts, lncRNAs lacked any notable pattern, suggesting that RNA secondary structure is a feature that can be used for categorization of protein‐coding transcripts and lncRNAs. The preservation of the positive correlation between protein‐coding and noncoding transcripts suggests that this relationship is a feature of nuclear RNAs and not a result of the protein‐coding capacity of mRNAs. Overall, while RNA secondary structure and protein binding are positively correlated in lncRNAs and larger regions of mRNAs, this is highly dependent on the specific regions that are interrogated.

### 3.3 RNA secondary structure of protein‐coding transcripts shows large‐scale changes in response to systemic salt stress

RNA secondary structure was previously shown to fluctuate in a developmental‐ (Beaudoin et al., 2018; Foley, Gosai, et al., 2017) and stress‐dependent manner (Tack et al., 2020), where it played a role in regulating mRNA fate. To determine if RNA secondary structure fluctuated upon salt stress, we directly compared RNA secondary structure in control‐ and salt‐treated tissue across the entire mRNA transcript. There were large rearrangements of RNA secondary structure upon exposure to salt stress (Figure S5c), particularly an increase in structure scores (more double‐stranded) in the 5’ UTR and CDS in salt‐treated tissue compared to control‐treated tissue (*p* = .089 and *p* < 2.2 × 10^–16^, respectively; Wilcoxon test; Figure S5c). In contrast, RNA secondary structure in the 3’ UTR was significantly lower (more single‐stranded) in salt‐treated tissue compared to control (*p* < 2.2 × 10^–16^; Wilcoxon test; Figure S5c). A previous study by Tack and colleagues examining RNA secondary structure in the total cellular RNA of shoots from 24‐day‐old Col‐0 plants treated with short‐term salt stress using a chemical‐based structure probing assay to modify ssRNA found a similar trend of structural changes (Tack et al., 2020).

To examine if RNA secondary structure is decided in the nucleus and maintained in the cytoplasm during salt stress response, we compared structure inferred by nucleotide reactivity to the chemical DMS by Tack and colleagues from whole shoot tissue treated with short‐term salt stress (Tack et al., 2020) to our nuclear structure scores calculated by PIP‐seq. While not overly striking, there was a significant correlation between average structure score calculated by PIP‐seq (where lower scores indicated lower structure/more single‐stranded) and reactivity (where higher reactivity indicated lower structure/more single‐stranded) in both control‐ and salt‐treated tissue, especially in the CDS but also in the 5’ UTR, 3’ UTR, and when the whole transcript was analyzed (Figure S6a–h). These findings suggest that RNA secondary structure formed in the nucleus is at least partly maintained upon export into the cytosol.

To get a more detailed view of the structure changes observed upon salt stress (Figure S5c), we specifically compared RNA secondary structure scores from control‐ and salt‐treated tissue in the 100 nt up‐ and downstream of the start and stop codon. As noted previously (Figure 1d,e), there was an increase in structure score from the 5’ UTR to the CDS and a dip in secondary structure (more single‐stranded) directly over the start codon in both control‐ and salt‐treated tissue (Figure 2). While patterns of structure scores were overall similar in control‐ and salt‐treated tissue, the dip in structure around the start codon in salt‐treated tissue was broader and less pronounced than that found in control‐treated tissue (grey highlight; *p* < 1.86 × 10^–9^; Wilcoxon test). This indicates that, during salt stress, a larger region upstream of the start codon is alleviated of secondary structure, possibly allowing for increased ribosome recognition of this transcript region during salt stress response, though further experiments are needed to directly test this hypothesis. At the stop codon, while the trend of decreased RNA secondary structure from the CDS to 3’ UTR was shared in control‐ and salt‐treated tissue, RNA secondary structure in the 3’ UTR was significantly lower in salt‐treated tissues compared to control‐treated, indicating a loss of structure during salt stress in this region (*p* < 2.2 × 10^–16^; Wilcoxon test; Figure 2a). Overall, RNA secondary structure significantly changes during salt stress response.

The changes observed in RNA secondary structure during salt stress indicate regulation by external factors since, if primary sequence was the sole factor driving RNA structure formation, the structures would look the same in control‐ and salt‐treated tissue. Given the positive correlation between RBP binding and RNA secondary structure (Figure 1d–g), we hypothesized that the changes in secondary structure observed during salt stress response may be due to changes in RBP binding density. To test this, we directly compared RBP binding of control‐specific, salt‐specific, and shared high‐confidence PPSs in the same 100 nt up‐ and downstream of the start and stop codon of mRNAs present in both control‐ and salt‐treated tissue. As was seen previously for all high‐confidence PPSs identified in control‐ and salt‐treated tissue (Figure 1d,e), the density of control‐specific, salt‐specific and shared high‐confidence PPSs increased over the start codon and decreased over the stop codon, with high protein binding throughout the CDS (Figure 2b). While there are few changes in RBP binding density between control‐ and salt‐treated tissue around the start codon, there was an increase in binding of control‐specific PPSs in the ~50 nt upstream of the stop codon compared to PPSs identified in either condition on its own, suggesting that RBPs that bind in this region may be important to regulate processes occurring specifically in control conditions (Figure 2b). Altogether, while the similarity of RBP binding densities for control‐ and salt‐specific PPSs indicates that it is unlikely that changes in global protein binding are the major cause of the changes in RNA secondary structure that were observed, the identity of the proteins bound may change and affect the structure, a subject for future studies.

To determine if the large changes in secondary structure observed during salt stress was specific to protein‐coding transcripts, we also examined RNA secondary structure across nuclear lncRNAs expressed in both control‐ and salt‐treated tissue. When comparing RNA secondary structure between control‐ and salt‐treated tissue along spliced lncRNAs, there were no substantial changes observed (Figure 2c). In fact, in both control‐ and salt‐treated tissue, lncRNAs had an average structure score of ~0.0 (dashed lines), indicating that there was enough coverage across the length of the lncRNA to calculate a structure score, but that there was an equal number of reads in the dsRNA‐seq and ssRNA‐seq libraries, resulting in a value of 0. This suggests that the RNA secondary structure of lncRNAs is dynamic, rapidly pairing, and unpairing throughout their lifecycle.

We also directly compared binding of control‐specific, salt‐specific, and shared high‐confidence RBP binding densities across the length of lncRNAs expressed in both treatments. On average, there was an increase in RBP binding for control‐specific PPSs compared to salt‐specific PPSs or those shared between conditions (Figure 2d). On the whole, nuclear lncRNAs do not have distinguishable profiles of RNA secondary structure or RBP binding. Similar to the case with mRNAs, while the presence of RBPs along the lncRNAs (RBP binding) is consistent between control‐ and salt‐treated tissue, the identity of the proteins bound likely helps define the function of these nuclear lncRNAs during salt stress response. In total, while mRNA secondary structure significantly changes during salt stress, this is a unique feature to protein‐coding transcripts. Furthermore, the similar RBP binding of control‐ and salt‐specific PPSs further suggests that RBP binding is not the sole cause of the changes in RNA secondary structure observed.

### 3.4 m^6^A density is anti‐correlated with mRNA secondary structure

While RNA secondary structure and RBP binding were positively correlated, RBP binding does not appear to be the primary cause of RNA secondary structure changes observed upstream of the start codon and in the 3’ UTR (Figure 2a and Figure S5c), leading to the question of what other mRNA features aid in these structural rearrangements. In recent years, m^6^A has been shown to function in nearly every step of post‐transcriptional gene regulation, including RNA secondary structure, nuclear export, mRNA stability, and translation (reviewed in Kramer et al., 2018). In plants, m^6^A has roles in development, leaf morphology, fruit ripening, and stress response, in particular response to salt stress (Liang et al., 2020). Since m^6^A is primarily located in the 3’ UTR of protein‐coding mRNAs (Anderson et al., 2018; Meyer et al., 2012; Shen et al., 2016) and can affect RNA secondary structure (Liu et al., 2015; Sun et al., 2019), we hypothesized that m^6^A may cause the large structural changes in the 3’ UTR observed in mRNAs between control‐ and salt‐treated plants (Figure 2a and Figure S5c).

We previously performed m^6^A RNA immunoprecipitation and sequencing (m^6^A‐seq) on polyA^+^ RNA from control‐ and salt‐treated rosette leaves and identified ~15,000 and ~17,000 m^6^A peaks present in both biological replicates from control‐ and salt‐treated tissue respectively (Anderson et al., 2018). Agreeing with previously published literature (Liu et al., 2015; Sun et al., 2019), these identified m^6^A peaks were localized primarily in the 3’ UTR and the stop codon of mRNAs (Anderson et al., 2018). While nearly 90% of high‐confidence m^6^A peaks identified in control‐treated tissue were also identified in salt‐treated tissue (shared; *N* = 13,375), distinct classes of m^6^A peaks were identified exclusively in control‐treated tissue (control‐specific; *N* = 1,731), or in salt‐treated tissue (salt‐specific; *N* = 4,473; Anderson et al., 2018). In fact, transcripts that gained m^6^A upon salt stress were enriched for mRNAs that encode proteins involved in salt and osmotic stress response, indicating a potential mechanism of regulation in response to salt stress (Anderson et al., 2018).

**FIGURE 3:**
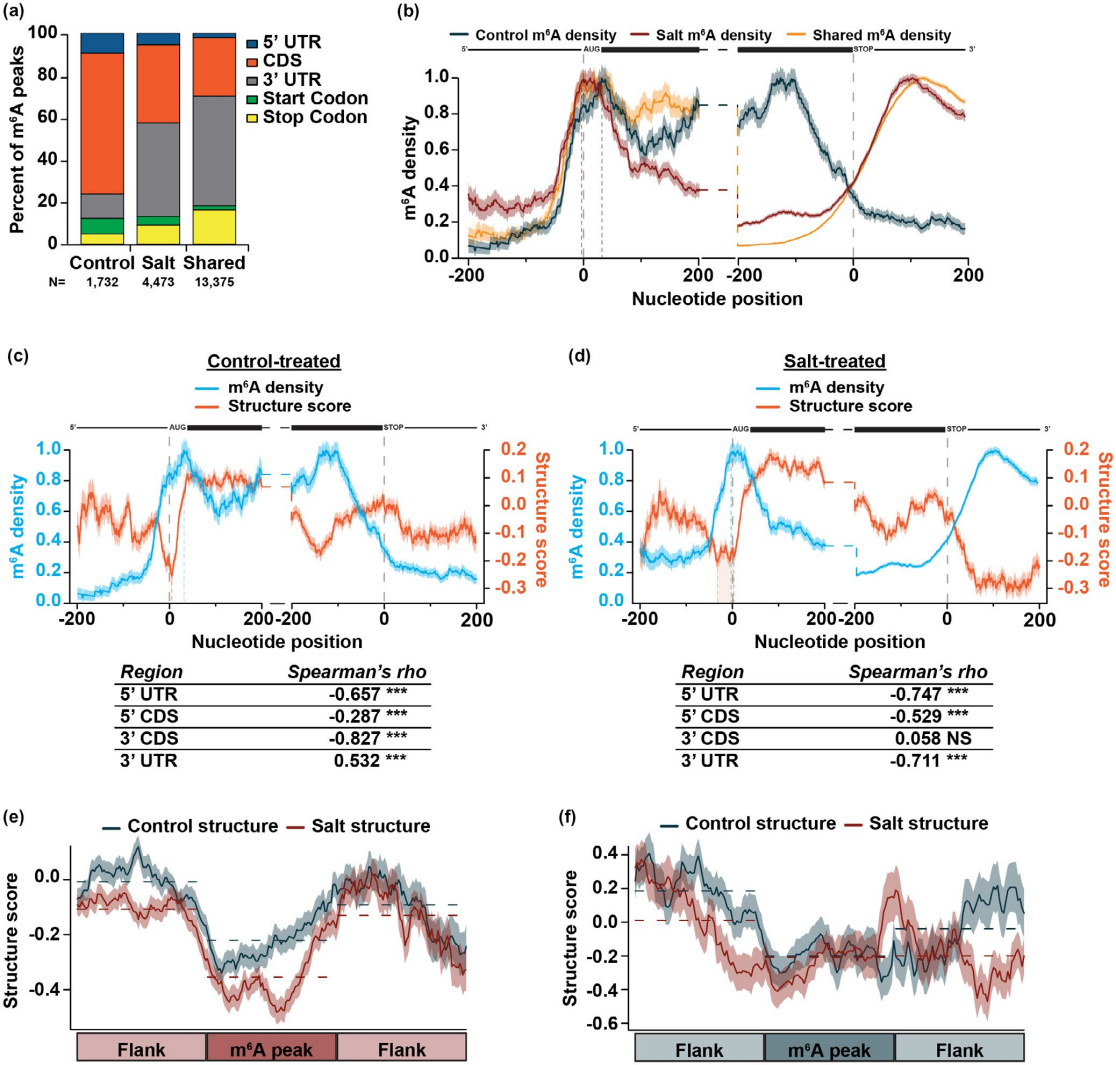
m^6^A is highly dynamic during exposure to long‐term salt stress response and is anti‐correlated with RNA secondary structure. (a) Classification for m^6^A peaks within protein‐coding genes found only in control‐treated tissue (*N* = 1,732 peaks), only in salt‐treated tissue (*N* = 4,473 peaks), or common to both (*N* = 13,375 peaks). (b) m^6^A density distribution in the ± 200 nt of the start and stop codon for control‐specific (blue), salt‐specific (red), and share m^6^A peaks (yellow). Dashed vertical lines near the start codon represent the apex of the peak in m^6^A density at the start codon for control‐treated (blue) and salt‐treated (red) tissue. *N* = 6,515 mRNAs. NS *p* > .05; *, **, and *** denote *p *< .05, 0.01, and 0.001, respectively, Spearman's asymptotic t approximation. mRNA diagrams above plots are not to scale. (c,d) Average m^6^A density (light blue line) and structure score (orange line) at each nucleotide ± 200 nt of the annotated start and stop codon in nuclear mRNAs in control‐treated (c) or salt‐treated (d) tissue. The tables represent Spearman's rho correlations between m^6^A density and structure score in the 5’ UTR, 5’ CDS, 3’ CDS, and 3’ UTR across all plotted transcripts. Shading around the line indicates the *SEM* across all plotted transcripts. *N* = 4,260 mRNAs. Dashed vertical light blue lines indicate the apex of the peak in m^6^A density at the start codon. Dashed orange lines indicate the dip in secondary structure at the start codon. Orange shading at the start codon represents the broad dip in salt stress (d). mRNA diagrams above plots are not to scale. (e‐f) RNA secondary structure scores in control‐treated (blue) and salt‐treated (red) tissues across binned salt‐specific (e) or control‐specific (f) m^6^A peaks located in the 3’ UTR and equal‐sized flanking regions to the 5’ and 3’ end. Dashed lines represent the average structure scores in each bin. Shading around the line indicates the *SEM* across all plotted transcripts.

While m^6^A is primarily located in the 3’ UTR and stop codon (Anderson et al., 2018; Liu et al., 2015; Sun et al., 2019), previous research showed that m^6^A localization can be dynamic during stress response (Zhou et al., 2018), where exposure to stress conditions causes a global shift in m^6^A location, ultimately affecting the fate of the mRNAs. Given these findings, we asked whether there was a shift in m^6^A location in a condition‐specific manner of high‐confidence control‐specific, salt‐specific, or shared m^6^A peaks. While m^6^A peaks common to both conditions and specific to salt‐treated tissue remained primarily located in the 3’ UTR, the majority of control‐specific m^6^A peaks were located in the CDS (Figure 3a,b), indicating that m^6^A deposition is indeed dynamic during systemic salt stress. In fact, while nearly 50% of salt‐specific m^6^A peaks were located in the 3’ UTR, only ~10% of control‐specific peaks were located in this region (Figure 3a).

We then took a closer look at this phenomenon by extracting transcripts that (1) contained m^6^A in control‐treated tissue, but lost all m^6^A in during salt treatment, (2) did not contain m^6^A in control‐treated tissue but gained m^6^A during stress, and (3) contained m^6^A in both conditions, but in independent locations (Figure S7a). m^6^A located on transcripts that were m^6^A modified in both conditions (Group 3) were located in the CDS and 3’ UTR in close to equal frequencies in control‐ and salt‐treated tissues (Figure S7b), suggesting that if a transcript is modified in both conditions, the new m^6^A added during salt stress occurs in a similar transcript location (i.e. loss in 3’ UTR in control and gain in this same region in salt; Figure S7a,b). However, the m^6^A events in transcripts that completely lose this mark upon salt stress remains primarily in the CDS, while upon salt stress, previously unmodified transcripts mostly gain m^6^A in the 3’ UTR (Figure S7b). Overall, this suggests that specific classes of transcripts are marked differentially in a condition‐specific manner and that the location of m^6^A within a transcript may be important for salt stress response.

To examine the pattern of m^6^A deposition on mRNAs in control‐ and salt‐treated tissue, we calculated m^6^A density using a similar calculation as for RBP binding. Each nucleotide was assigned a score of 1 or 0 based on whether or not a m^6^A peak was identified at that nucleotide, with 1 indicating that nucleotide is within a m^6^A peak and 0 indicating that nucleotide is not within a m^6^A peak. m^6^A density is then graphed such that the highest region of occupancy is normalized to a density of 1.0. We focused on the 200 nt up‐ and downstream of the start and stop codon to incorporate more of the CDS and 3’ UTR to better visualize m^6^A dynamics. In agreement with m^6^A classification (Figure 3a), there was a large shift visible between control‐ and salt‐specific m^6^A density (Figure 3b), particularly in the 3’ CDS and UTR.

As this shift in m^6^A density in the 3’ UTR occurred in the same region as the large decrease in RNA secondary structure during salt stress (Figure 2a), we next directly compared RNA secondary structure and m^6^A density. To do so, we again focused on the 200 nt up‐ and downstream of the start and stop codon and found that m^6^A density was strongly anti‐correlated with RNA secondary structure. Specifically, an increase in m^6^A density was accompanied by a decrease in RNA secondary structure and vice versa (Figure 3c,d). This was particularly evident for salt‐specific m^6^A sites, as the increase in m^6^A density in the 3’ UTR in salt‐treated tissue was accompanied by a significant decrease in RNA secondary structure (Spearman's rho <−0.711; *p* < 2.2 × 10^–16^; asymptotic t approximation; Figure 3d). Similarly, the increase of m^6^A density in the 3' CDS of control‐treated tissue was accompanied by a decrease in RNA secondary structure in the same region (Figure 3c). Interestingly, the regions with the highest changes in m^6^A density for control‐treated (3’ CDS) and salt‐treated (3’ UTR) tissue demonstrate the largest anti‐correlations, suggesting that the high density of m^6^A in these regions resulted in drastic decreases in RNA secondary structure.

Additionally, towards the 5’ end of transcripts, the strong dip in RNA secondary structure observed at the start codon previously (Figure 2a) was concurrent with a peak in m^6^A density at the same position (Figure 3c,d). There was a shift in the m^6^A density distribution upstream of the start codon between control‐ and salt‐specific m^6^A peaks, where salt‐specific m^6^A density tended to peak ~20 nt upstream of control‐specific m^6^A density (Figure 3b; vertical dashed red and blue lines). This shift may result in the decrease in RNA secondary structure in salt‐treated tissue observed upstream of the start codon (Figures 2a and 3d). Previous studies demonstrated that m^6^A deposition in the 5’ UTR results in increased translation (Meyer et al., 2015) and as mentioned earlier, the characteristic dip in mRNA secondary structure at the start codon in eukaryotes is hypothesized to permit recognition of the start codon by translation machinery. While future studies are required to confirm this, we hypothesize that this shift in m^6^A density and associated widening of the dip in RNA secondary structure at the start codon in salt‐treated tissue may lead to increased translation when exported into the cytoplasm. Overall, our findings reveal that m^6^A density and mRNA secondary structure are highly anti‐correlated.

To interrogate if m^6^A was directly responsible for the changes in structure observed, we examined RNA secondary structure scores directly at control‐ and salt‐specific m^6^A peaks located in the 3’ UTR, as the largest changes in both m^6^A density and mRNA secondary structure are in this region. To do this, we took the entire length of the control‐ and salt‐specific high‐confidence m^6^A peaks, divided each peak into equal sized bins and graphed the average structure score along the length of these peaks as well as equal sized regions flanking the m^6^A peaks. At salt‐specific m^6^A peaks located in the 3’ UTR, there was a significant decrease in RNA secondary structure in salt‐treated tissue compared to control (Figure 3e; *p* < 2.2 × 10^–16^; Wilcoxon test). There was also a significant loss of RNA secondary structure in salt‐treated tissue in the region upstream of the m^6^A peak, suggesting that salt‐dependent m^6^A deposition causes loss of structure not only at the m^6^A peak, but can also affect structure of a wider distance (Figure 3e; *p* < 2.2 × 10^–16^; Wilcoxon test). This pattern was specific to m^6^A peaks as shuffled, equal‐sized control regions did not show this structural pattern (Figure S7c,d). Additionally, this change in structure results in an overall decrease in RBP binding as compared to control conditions likely from a decrease in control‐specific RBP binding events. Overall, these results suggest that an increase in RBP binding events is not the main driver of these structural changes (Figure S7e,f).

To determine if this local change in structure was a feature common to all m^6^A sites, we examined RNA secondary structure at control‐specific m^6^A sites located in the 3’ UTR as well. While one might expect that there would be lower structure during control conditions compared to salt stress conditions at control‐specific m^6^A sites, we did not see this trend (Figure 3f). This may be due to the major shift in localization of m^6^A in control conditions, resulting in significantly fewer m^6^A peaks located in the 3’ UTR in control conditions compared to salt (Figure 3a,b and Figure S7b). Overall, our results suggest that salt‐dependent m^6^A located in the 3’ UTR can cause significant local changes in RNA secondary structure in the Arabidopsis transcriptome.

### 3.5 Changes in mRNA secondary structure alone are not sufficient to alter the abundance of mRNAs during plant salt stress

**FIGURE 4:**
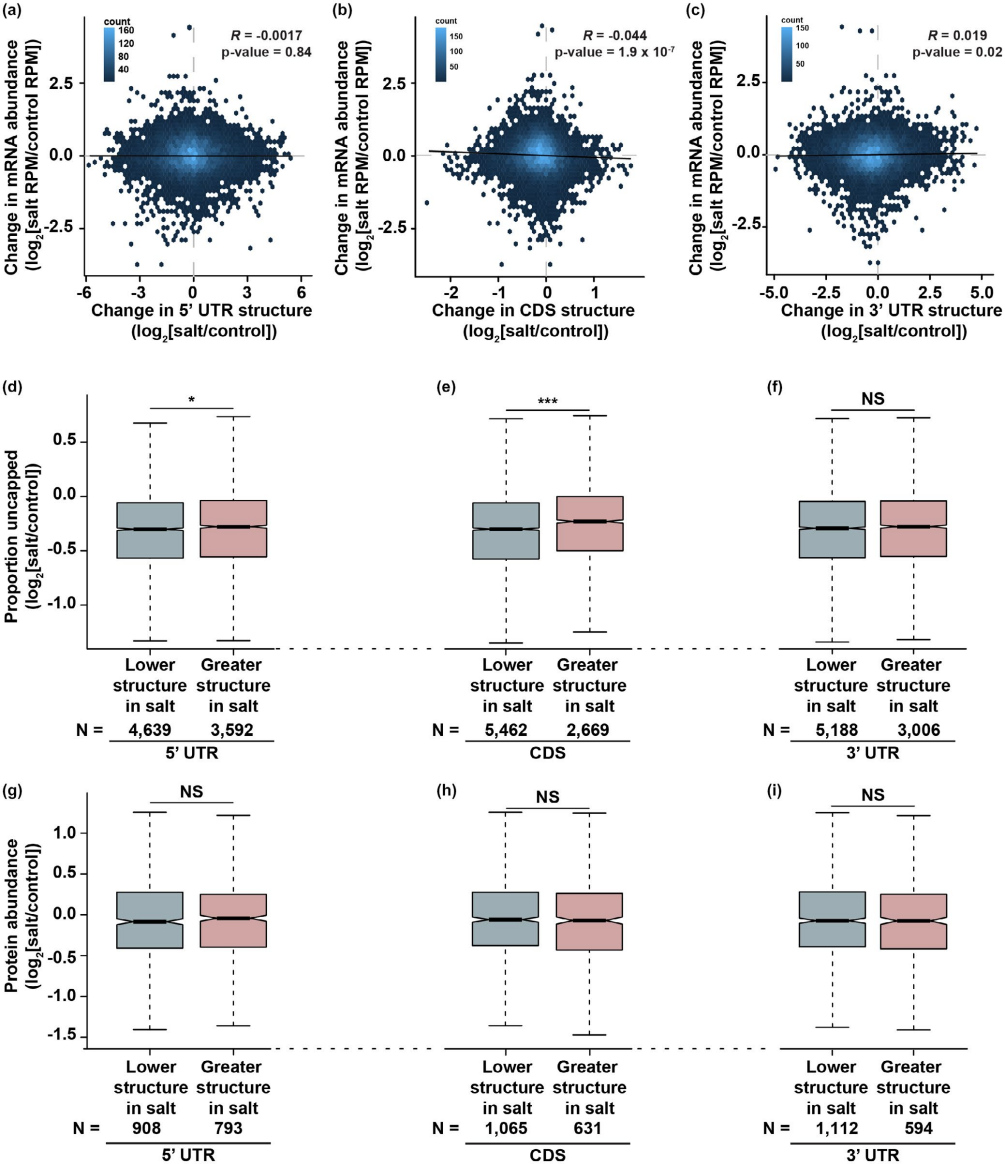
RNA secondary structure alone does not substantially affect mRNA abundance, stability, or translation output. (a–c) mRNA abundance fold change (y‐axis; log_2_[RPM_Salt_/RPM_Control_]) compared to RNA secondary structure fold change (x‐axis; log_2_[avg. structure score_Salt_/avg. structure score_Control_]) in the 5’ UTR (a), CDS (b), and 3’ UTR (c). Plots were made using geom_hex in the ggplot2 package in 50 bins. Color of each bin indicates the number of transcripts that fall within that range. R and *p*‐value calculated from Pearson coefficient. Solid black line represents the linear regression of each plot. *N* = 14,313. See Data Set S7. (d–f) Proportion uncapped fold change (log_2_[proportion uncapped_Salt_/proportion uncapped_Control_]) for transcripts that lose (light blue; log_2_[avg. structure score_Salt_/avg. structure score_Control_] <0) or gain (light red; log_2_[avg. structure score_Salt_/avg. structure score_Control_] >0) RNA secondary structure in the 5’ UTR (D), CDS (e), or 3’ UTR (f). *p < .05; **p < .001; NS denotes *p* > .05, Wilcoxon test. (g–i) Protein abundance fold change (log_2_[salt/control]) for transcripts that lose (light blue; log_2_[avg. structure score_Salt_/avg. structure score_Control_] <0) or gain (light red; log_2_[avg. structure score_Salt_/avg. structure score_Control_] >0) RNA secondary structure in the 5’ UTR (g), CDS (h), or 3’ UTR (I). NS denotes *p* > .05, Wilcoxon test. See Data Set S8.

RNA secondary structure was previously demonstrated to regulate many post‐transcriptional processes including mRNA translation and stability (Beaudoin et al., 2018; Goodarzi et al., 2012; Sun et al., 2019). In fact, a recent study examining mRNA secondary structure during short‐term salt stress in found a negative correlation between mRNA abundance and secondary structure in the 5’ UTR, CDS, and 3’UTR, suggesting that transcripts that have lower structure in the context of salt stress response are less abundant (Tack et al., 2020). To determine if this was also true in our study of long‐term salt stress, we calculated the fold change of structure score (FC_structure_ = log_2_[salt/control]) in the 5’ UTR, CDS, and 3’ UTR for transcripts expressed in both control‐ and salt‐treated tissue (Data Set S7), where values higher than 0 are more structured in salt‐treated tissue and vice versa. We then compared secondary structure fold change in each region to changes in mRNA abundance from our previously published mRNA‐seq experiment performed in control‐ and salt‐treated tissues from these same treatment conditions (log_2_[RPM_salt_/RPM_control_]) (Anderson et al., 2018; Figure 4a–c). In contrast with Tack and colleagues, there was no substantial relationship between changes in mRNA secondary structure in any region and mRNA abundance (Figure 4a–c; 5’ UTR: R = −0.0017; CDS: R = −0.044; 3’ UTR: R = 0.019; Pearson correlation). Direct comparison of average structure score and mRNA abundance in control‐ or salt treated tissue found similar trends (Figure S8a–f). Of note, while average structure score in the CDS was anti‐correlated in control‐treated tissue, with lower abundant genes being more structured (Figure S8b; R = −0.06; *p* = 9.0 x 10^–13^; Pearson correlation), the opposite was observed in salt‐treated tissue with lower abundant genes being less structured (Figure S8e; R = 0.049; *p* = 3.9 × 10^–9^; Pearson correlation). Overall, this suggests that changes in mRNA secondary structure alone are insufficient to affect transcript abundance during long‐term salt stress response, but this is condition‐specific and highly dependent on the region of secondary structure interrogated.

Since mRNA secondary structure also contributes to regulation of mRNA stability and translation (Beaudoin et al., 2018; Goodarzi et al., 2012; Sun et al., 2019), we next asked if changes in mRNA secondary structure in the 5’ UTR, CDS, or 3’ UTR affected these two processes. To examine mRNA stability, we used our previously published global mapping of uncapped and cleaved transcripts (GMUCT) data from control‐ and salt‐treated tissue (Anderson et al., 2018) to calculate the proportion uncapped metric, which is the log_2_ ratio of RPM from GMUCT for a given transcript to total mRNA‐seq for the same transcript (log_2_[RPM_GMUCT_/RPM_mRNA‐seq_]) (Anderson et al., 2018; Vandivier et al., 2015; Willmann, Berkowitz, & Gregory, 2014). This metric was previously shown to be a good measure of mRNA stability, with higher proportion uncapped values indicating transcript instability and vice versa (Anderson et al., 2018; Vandivier et al., 2015). We then calculated the fold change in proportion uncapped between salt‐ and control‐treated tissue (log_2_[proportion uncapped_Salt_/proportion uncapped_Control_]), where a fold change greater than 0 indicates that a transcript is destabilized in salt‐treated tissue and vice versa. To determine if changes in mRNA secondary structure regulated mRNA stability, we compared proportion uncapped fold change for transcripts that lost (FC < 0; light blue) or gained (FC > 0; light red) mRNA secondary structure in the 5’ UTR, CDS, or 3’ UTR during salt stress (Figure 4d–f, Data Set S7). While changes in RNA secondary structure in the 3’ UTR during salt stress did not significantly change (*p* > 0.05; Wilcoxon test) mRNA stability (Figure 4f), transcripts that had greater structure in salt conditions in the 5’ UTR and CDS were significantly (5’ UTR: *p* < .05; CDS: *p* < .001; Wilcoxon test) destabilized during salt stress. Thus, the role of mRNA secondary structure in regulation of mRNA stability may be dependent on the region of the transcript that alters in structure.

Lastly, to determine if mRNA structure contributes to protein abundance, we performed mass spectrometry on protein lysates isolated from control‐ and salt‐treated tissue. We then calculated protein abundance fold change as the ratio of average iBAQ intensities in salt‐treated tissue compared to those in control‐treated tissue (log_2_[salt/control] ; Data Set S8). Similar to the results observed for mRNA abundance and stability, there were no effects of changes in 5’ UTR, CDS, or 3’ UTR mRNA secondary structure on protein production (Figure 4g–i). It is of note that due to the lower sensitivity of mass spectrometry compared to RNA‐seq technologies, we are restricted in the number of proteins identified, thus the N of our proteomics data is substantially lower than that of mRNA‐seq and GMUCT. Overall, in our system, mRNA structure by itself does not substantially regulate mRNA abundance, stability, or protein levels. Given the low correlation between nuclear RNA secondary structure calculated by this study and total cell RNA secondary structure calculated by Tack and colleagues (Figure S6), it is possible that the differences in secondary structure that occur after export from the nucleus are primarily the cause of the relationship between RNA secondary structure and mRNA abundance they observed. Additionally, prior studies found that the association between lower structure and increased translation is a result of unzipping of RNA secondary structure by the ribosome, rather than the lower structure promoting increased translation (Beaudoin et al., 2018). Thus, it is possible that the changes in RNA secondary structure is linked to another process/signal and this process/signal affects mRNA fate.

### 3.6 m^6^A deposition and stabilization is concurrent with changes in mRNA secondary structure and increases in protein abundance for transcripts encoding stress related proteins

**FIGURE 5:**
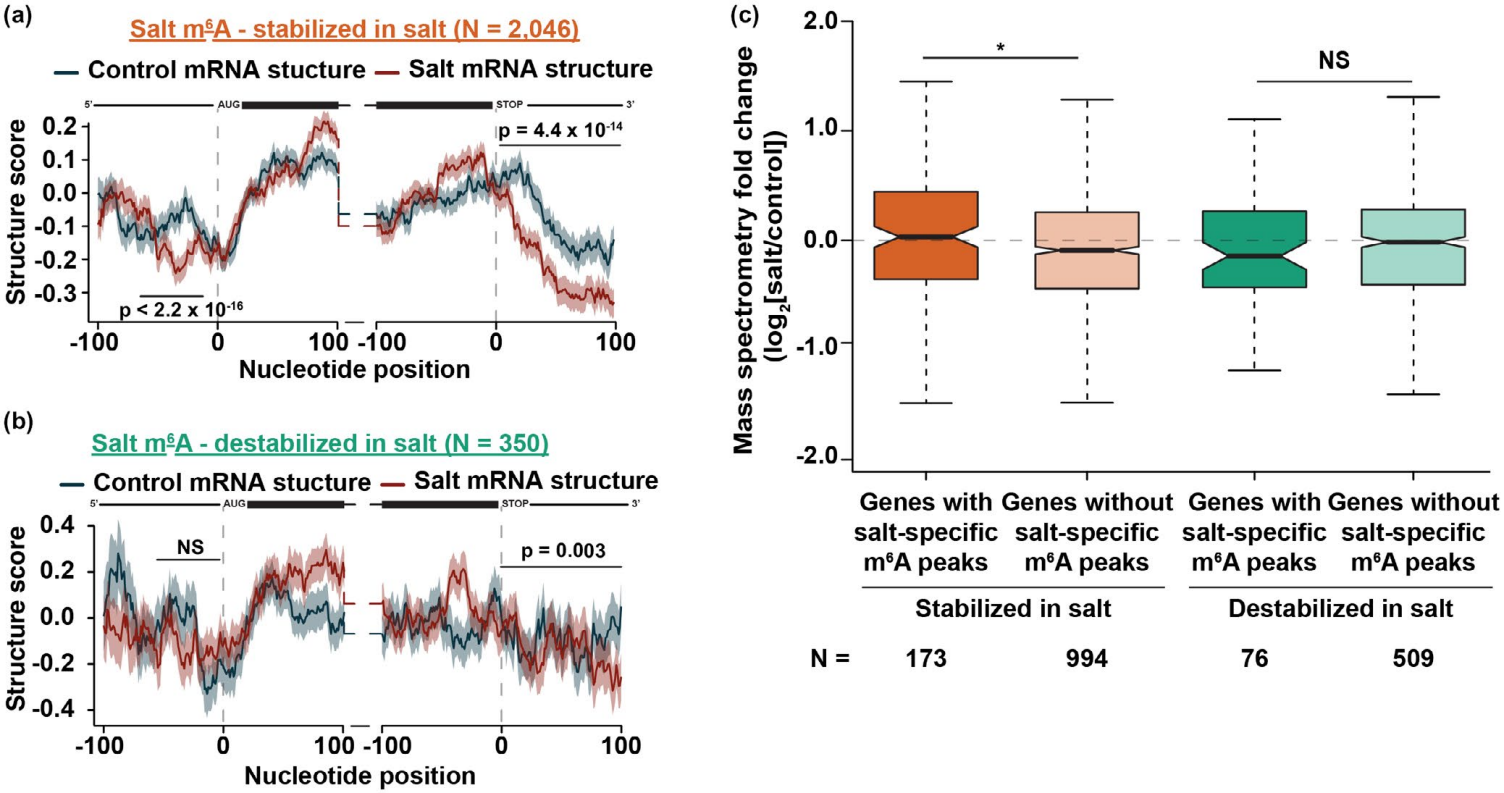
Transcripts that gain m^6^A and are stabilized upon systemic salt stress response lose RNA secondary structure at the start codon and 3’ UTR and produce more protein. (a,b) Average structure score in control‐treated (blue line) and salt‐treated (red line) tissue in the ±100 nt of the annotated start and stop codon of nuclear protein‐coding transcripts that gain m^6^A and are stabilized (a) or destabilized (b) during long‐term salt stress response. See Data Set S9. Shading around the line indicates the *SEM* across all plotted transcripts. *p*‐values were calculated using a Wilcoxon test and are denoted over the specific regions. mRNA diagrams above plots are not to scale. (c) Protein abundance fold change (log_2_[salt/control]) for transcripts that contain salt‐specific m^6^A peaks (darker colors) or lack salt‐specific m^6^A peaks (lighter colors) and are stabilized (orange) or destabilized (green) during salt stress response. NS and *p > .05 or <.05, respectively, Wilcoxon test. See Data Set S8.

We previously found that, upon salt stress, m^6^A was specifically deposited on transcripts encoding proteins involved in osmotic and salt stress response and these transcripts were significantly more stable in salt‐treated tissue than those that lacked m^6^A (Anderson et al., 2018). While this is generally the case, there is still a subset of transcripts that are destabilized, even with the addition of m^6^A, thus we speculated that the large changes in RNA secondary structure might help determine whether a transcript that gains m^6^A is stabilized or destabilized during salt stress. To test this, we first extracted transcripts that gained m^6^A specifically during salt stress and were either destabilized (*N* = 436) or stabilized (*N* = 1,981; Data Set S9; Anderson et al., 2018) during salt stress and examined secondary structure scores in the 100 nt ± the start and stop codon. While transcripts that gained m^6^A and were stabilized during salt stress maintained the salt‐dependent structural rearrangements observed previously in the 50 nt upstream of the start codon and in 3’ UTR (Figure 2a), those that gained m^6^A but were destabilized did not maintain these structural rearrangements (Figure 5a,b). Importantly, the location of salt‐specific m^6^A sites is similar for transcripts that were stabilized or destabilized (Figure S9a), thus the changes observed were not due to changes in m^6^A localization. These results suggest that the combination of m^6^A deposition and the corresponding loss in secondary structure in response to long‐term salt stress plays a role in transcript stabilization through an unknown direct or indirect mechanism.

Though lower RNA structure tends to be correlated with increased degradation by exonucleases (Beaudoin et al., 2018), and we previously saw no relationship between changes in 3’ UTR structure and mRNA stability (Figure 4f), transcripts that gain m^6^A and are stabilized have lower structure in their 3’ UTR. Thus, it is possible that the combination of m^6^A deposition and a decrease in secondary structure could permit certain RBPs to bind, resulting in the stabilization we observed. While global RBP binding doesn't change for high‐confidence control‐specific, salt‐specific, or shared PPSs for transcripts that gain m^6^A and are stabilized or destabilized (Figure S9b,c), the identity of the proteins binding likely changes. Additionally, the identity of the RBPs bound to stabilized or destabilized transcripts may also contribute the structural changes observed. Thus, the loss of structure for transcripts that demonstrate m^6^A‐associated stabilization may allow for salt‐specific proteins to bind and contribute to the increased mRNA stability of these transcripts in a salt‐dependent manner. Future studies will be focused on identifying RBP motifs in the regions that are more single‐stranded in the 3’ UTR upon salt stress and contain salt‐dependent m^6^A. These motifs can then be used to identify RBPs that bind to that specific sequence in a salt‐dependent manner, as this methodology has been successfully used to identify novel nuclear RBPs and regulators of root hair cell fate previously (Foley, Gosai, et al., 2017; Gosai et al., 2015).

As noted above, we previously observed that transcripts that gain m^6^A upon salt stress were transcripts involved in stress response, most notably response to salt and osmotic stress, but also a variety of other stresses including response to wounding, cold, jasmonic acid, abscisic acid, and oxidative stress (Anderson et al., 2018). To determine what transcripts gained m^6^A and were stabilized or destabilized during salt stress, we performed a gene ontology (GO) analysis using DAVID (Huang et al., 2009) on these subsets of transcripts. Transcripts that were stabilized by m^6^A during salt stress were enriched for genes involved in osmotic stress response while those that were destabilized were enriched for genes involved in other abiotic stresses, such as cold and abscisic acid (Figure S9d). Since the plants were exposed to a long‐term salt stress experiment, at the time of tissue collection, the plants were mostly affected by the lack of available water due to the high concentrations of NaCl, thus the m^6^A deposition and stabilization of transcripts encoding osmotic response proteins fits with the model of salt and osmotic adaptation as expected. We posit a model in which m^6^A is initially deposited on transcripts involved in several different abiotic stresses as an initial response to the stress. However, over time the plant better recognizes the specific stress as salt/osmotic stress, and thus degrades those transcripts involved in other abiotic stresses, as they are not needed for that specific stress response, resulting in the destabilization of transcripts involved in cold and abscisic acid stress during salt stress, despite the presence of m^6^A. Future studies are required to measure the direct role of m^6^A on mRNA stability during salt stress.

We previously hypothesized that the m^6^A deposition and subsequent stabilization during salt stress functioned to allow for increased protein levels of osmotic and salt stress related proteins and proper salt stress response (Anderson et al., 2018). To test this hypothesis, we measured total protein abundance by mass spectrometry in control‐ and salt‐treated tissue and calculated protein abundance fold change (log_2_[salt/control]) for transcripts that contained (darker colors) or lacked m^6^A (lighter color) and were stabilized (orange) or destabilized (green) upon salt stress (Data Set S9). Transcripts that gained m^6^A and were stabilized were found to produce significantly (*p* < .05; Wilcoxon test) more protein than those that were stabilized but lacked m^6^A (Figure 5c). Moreover, transcripts that gained m^6^A but were destabilized produced less protein in salt‐treated tissue than control‐treated (fold change <0), though this difference does not reach statistical significance (*p* > .05; Wilcoxon test). While we hypothesize that the increase in protein abundance is due to an increase in translation of transcripts required for salt stress response, we cannot rule out that decreases in protein degradation causes the increase in protein abundance observed. Future ribosome profiling studies in control‐ and salt‐treated tissue to track ribosome progress along transcripts that gain m^6^A and are stabilized during salt stress will help distinguish between these two possibilities.

As a control, we also examined genes with or without control‐specific m^6^A peaks that are stabilized or destabilized during salt stress response (Figure S10). Transcripts that have control‐specific m^6^A maintain the loss of RNA secondary structure in the 3’ UTR during salt stress regardless of whether they are stabilized or destabilized (Figure S10a,b). The presence of control‐specific m^6^A also does not appear to regulate protein abundance (Figure S10c) and is still enriched in the CDS regardless of stability (Figure S10d), suggesting that the location of m^6^A within a transcript is essential for affecting mRNA abundance, stability, and secondary structure (Figure 3a,b).

**FIGURE 6:**
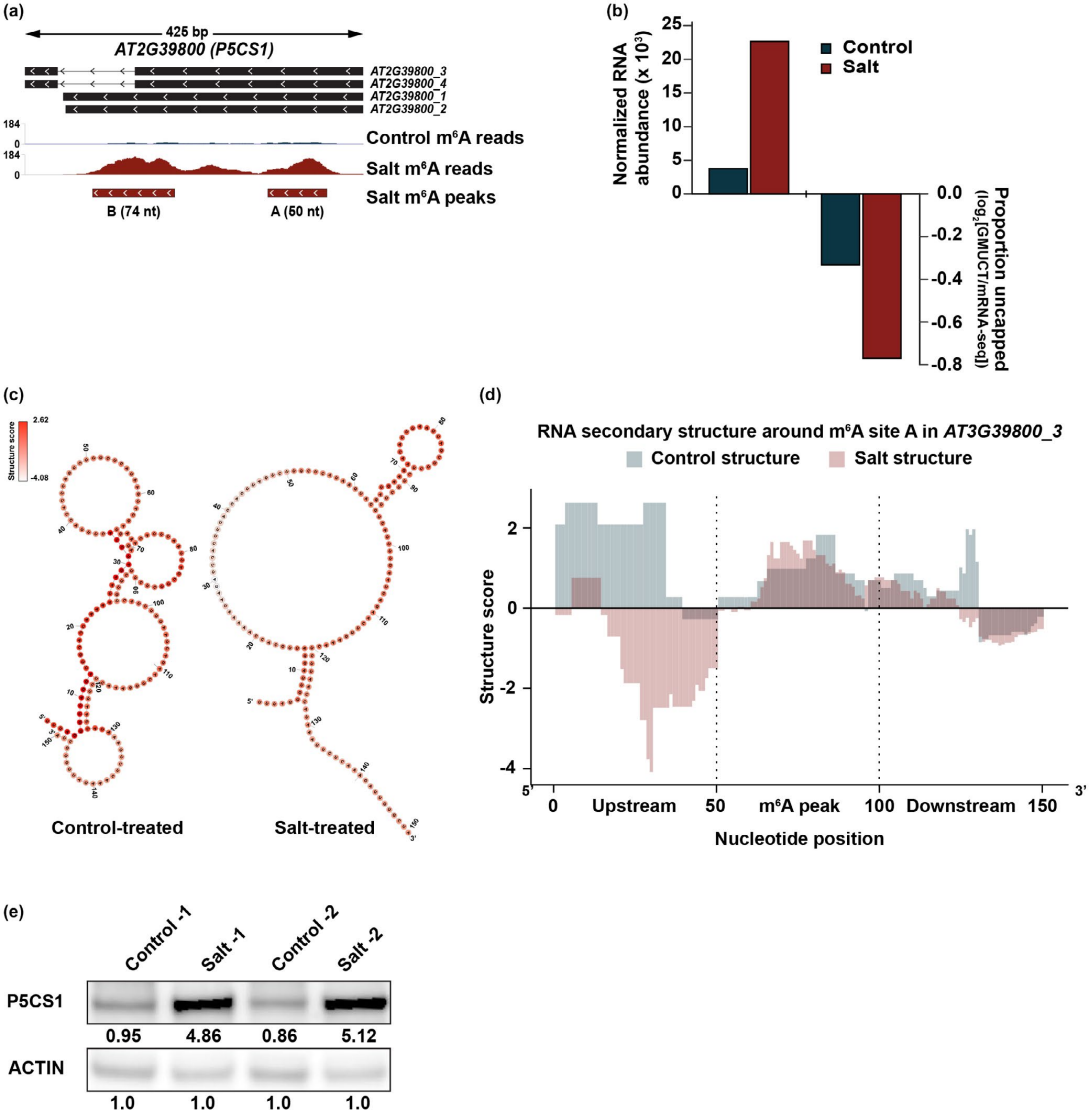
m^6^A modified, salt stress related gene *P5CS1* loses structure, is stabilized and its protein abundance increases during salt stress. (a) Representative image of the location of two salt‐specific m^6^A sites (denoted in the salt m^6^A peaks track) found in *AT2G39800* (*P5CS1*) and read coverage from m^6^A‐seq in control‐treated (blue) and salt‐treated (red) tissue. (b) Normalized RNA abundance calculated by DESeq2 and proportion uncapped in control‐ and salt‐treated tissue for *AT2G39800*. (c) RNA fold model for m^6^A peak A in *AT2G39800* in control‐ (left) and salt‐treated (right) tissue constrained with PIP‐seq determined structure scores. Color of each nucleotide indicates structure score, with darker colors indicating higher structure score. (d) RNA structure score scores from PIP‐seq for peak A within *AT2G39800* and equal sized regions flanking to the 5’ and 3’ end. (e) Western blot in control‐ and salt‐treated tissue for P5CS1 and ACTIN. Quantifications were calculated as previously described (Davarinejad, 2015).

**FIGURE 7:**
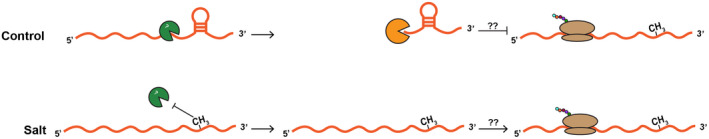
Hypothesized model of the role of m^6^A and mRNA secondary structure in transcript stabilization and translation during salt stress response. m^6^A is specifically deposited on transcripts encoding proteins involved in osmotic stress response in salt‐treated tissue where it relieves RNA secondary structure in the 3’ UTR and protects from degradation. This allows for translation of these transcripts and proper salt stress response.

To test the model transcripts that have m^6^A and are stabilized during salt stress indeed produce more protein, we focused on the salt stress related transcript *AT2G39800* (*DELTA 1‐PYRROLINE‐5‐CARBOXYLATE SYNTHASE; P5CS1*). *P5CS1* encodes an enzyme that catalyzes the rate‐limiting step in the biosynthesis of proline (Yoshiba et al., 1995) and is known to function during water deprivation, desiccation, and salt stress response (Feng et al., 2016; Székely et al., 2008). In fact, plants lacking P5CS1 are highly sensitive to water stress (Chen et al., 2018). Our results revealed that *P5CS1* contains two salt‐specific m^6^A peaks in its 3’ UTR (Figure 6a; denoted peak A and B), increases in RNA abundance, is stabilized upon salt stress (Figure 6b; Data Set S9), and loses RNA secondary structure in the area surrounding its two m^6^A peaks (Figure 6c,d and Figure S11a,b). In western blots of protein lysates from two biological replicates, P5CS1 indeed increased ~5‐fold in protein abundance in salt‐treated tissue compared to control (Figure 6e), further supporting the hypothesized model that deposition of m^6^A, and the associated mRNA stabilization and loss of RNA secondary structure in salt stress correlates with an increase in protein abundance (Figure 7).

In conclusion, using PIP‐seq, we identified RBP‐RNA interactions transcriptome‐wide and globally profiled nuclear RNA secondary structure during systemic salt stress in Arabidopsis (Figure 1). While the patterns of RBP‐RNA interactions are generally unchanged during systemic salt stress, whether there is binding of control‐ or salt‐specific RBPs during stress response remains an avenue for future research. Furthermore, these analyses reveal that RNA secondary structure significantly changes during systemic salt stress, in agreement with prior studies of RNA secondary structure in whole cell RNA (Tack et al., 2020). It is possible that these observed salt‐dependent changes in secondary structure are due to salt‐dependent m^6^A deposition that helps alleviate RNA secondary structure (Figure 3). Moreover, during our systemic salt stress treatment, changes in RNA secondary structure alone are generally insufficient to regulate mRNA fate as measured by mRNA abundance, stability, and protein output, though this is in part reliant on the genic region examined (Figure 4). While this is the case, it appears that the combination of salt‐specific deposition of m^6^A on transcripts encoding proteins involved in osmotic stress response and associated decreases in RNA secondary structure results in increases in transcript stability and protein abundance. In total, our findings suggest a model wherein m^6^A is deposited on and stabilizes transcripts encoding proteins involved in osmotic stress response, and these transcripts experience an associated decrease in RNA secondary structure and ultimately an increase in protein abundance (Figure 7). This increase in protein abundance may be due to an increase in translation or a decrease in protein degradation. Given the increase in stability for these transcripts, we favor the hypothesis that m^6^A deposition and stabilization leads to increased translation of proteins required for response to salt stress. Overall, our findings uncover evidence of an epitranscriptome, secondary structure‐mediated post‐transcriptional regulatory mechanism involved in plant long‐term salt stress response and adaptation.

## AUTHOR CONTRIBUTIONS

B.D.G. conceived the study. M.C.K., B.A.G., M.A.B., and B.D.G. designed the experiments. M.C.K., K.A.J., K.R.P., L.E.V., A.D.L.N., and B.D.G. performed the experiments. M.C.K., K.A.J., B.A.G., and B.D.G. analyzed the data. M.C.K. and B.D.G. wrote the paper with assistance from all authors. The authors have read and approved the manuscript for publication.

## Supporting information

Data Set S1‐S9

Figures S1‐S11

Supplementary Material

## Data Availability

**Accession numbers**: The raw and processed data for m^6^A‐seq, RNA‐seq, and GMUCT from our analyses of control‐ and salt‐treated Arabidopsis tissue were previously deposited into the NCBI Gene Expression Omnibus (GEO) database under the accession number GSE108852. The raw and processed data for PIP‐seq from our control‐ and salt‐stressed tissue produced for this study have been deposited into the NCBI GEO database under accession number GSE147812. **Genome browser availability**: The sequencing data presented here is also available through the EPIC‐CoGe genome browser: https://genomevolution.org/coge/NotebookView.pl?nid=2717.
