# Supplementary material for: N6‐methyladenosine and RNA secondary structure affect transcript stability and protein abundance during systemic salt stress in Arabidopsis
Source: Plant Direct. 2020 Jul 24;4(7):e00239. doi: 10.1002/pld3.239 (PMC7379018; doi:10.1002/pld3.239)
Supplement: Supplementary file 2 — Figures S1‐S11 [file PLD3-4-e00239-s002.pdf]

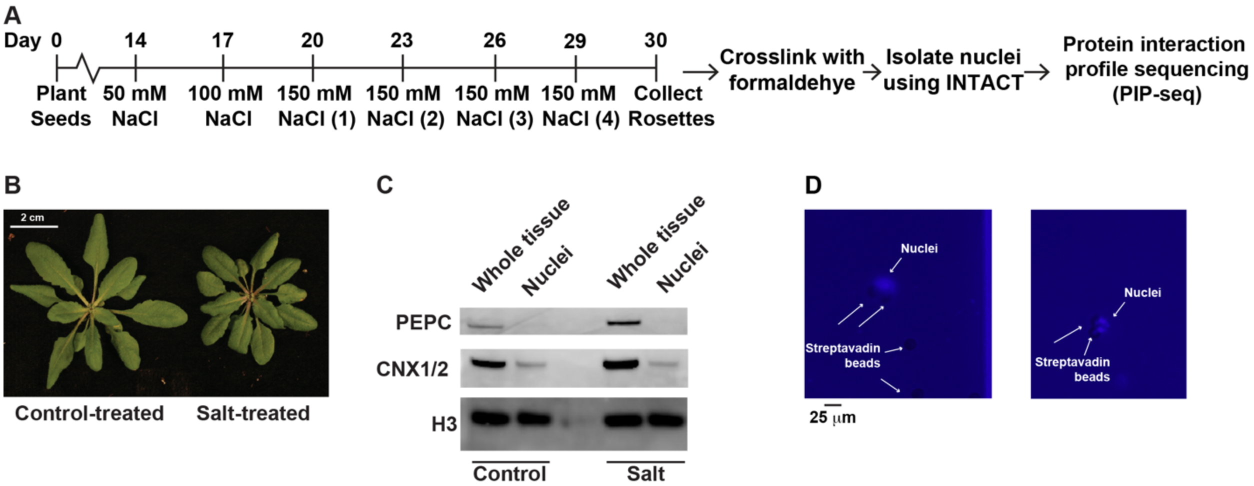

Supplemental Figure 1: INTACT to isolate nuclei from plants under systemic salt stress

(A) Timeline of salt stress experiment.

(B) Representative photos of Arabidopsis rosette leaves after control- or salt-treatment.

(C) Western blot in whole tissue and nuclear samples in control- and salt-treated tissue isolated by INTACT for cytoplasmic, endoplasmic reticulum, and nuclear markers (PEPC, CNX1/2, H3, respectively).

(D) Representative images of bead-bound nuclei from INTACT.

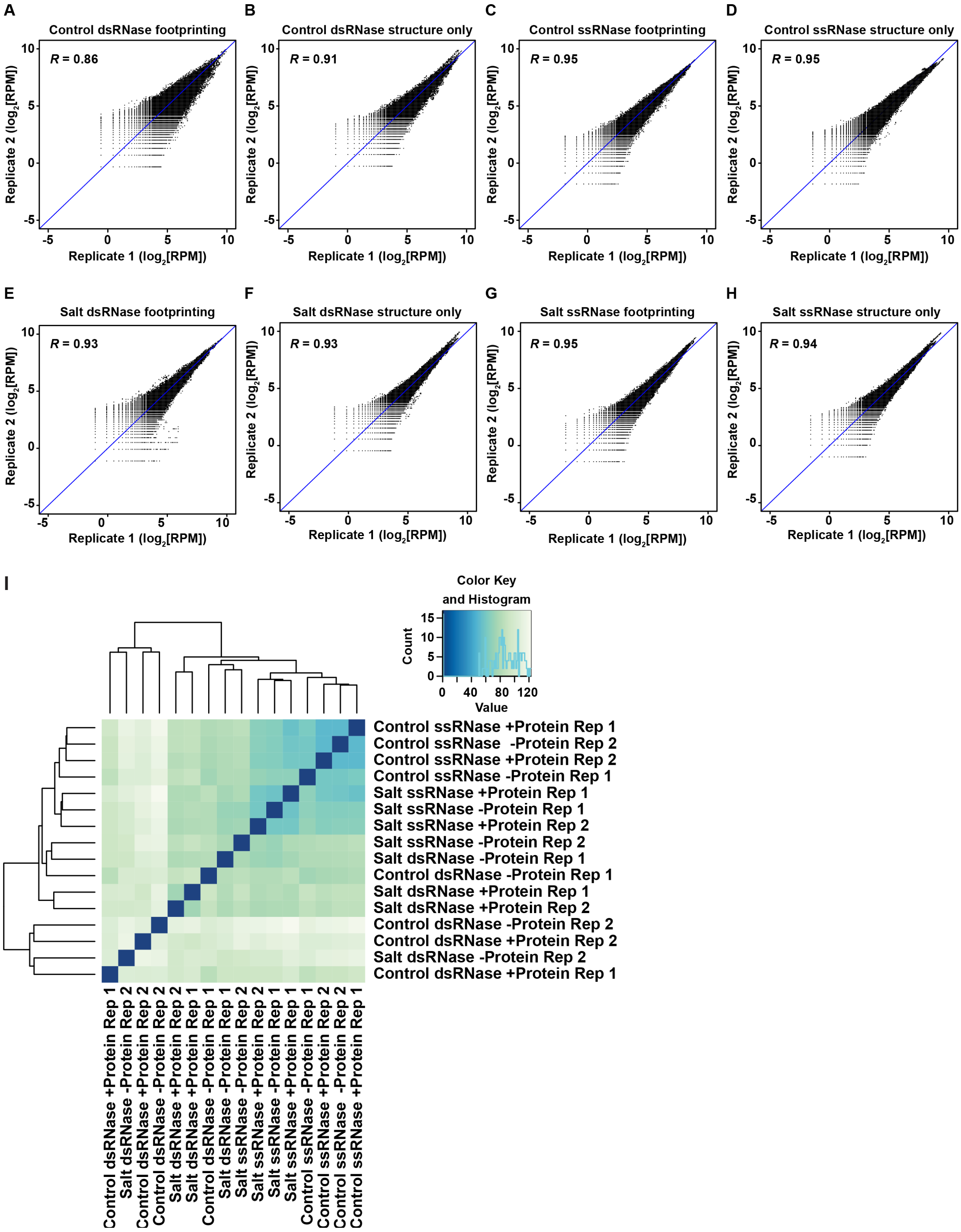

A
Control dsRNase footprinting
R = 0.86
B
Control dsRNase structure only
R = 0.91
C
Control ssRNase footprinting
R = 0.95
D
Control ssRNase structure only
R = 0.95
E
Salt dsRNase footprinting
R = 0.93
F
Salt dsRNase structure only
R = 0.93
G
Salt ssRNase footprinting
R = 0.95
H
Salt ssRNase structure only
R = 0.94
Replicate 2 (log2[RPM])
Replicate 1 (log2[RPM])
-5
0
5
10
I
Color Key
and Histogram
Count
Value
0
5
10
15
40
80
120
Control ssRNase +Protein Rep 1
Control ssRNase -Protein Rep 2
Control ssRNase +Protein Rep 2
Control ssRNase -Protein Rep 1
Salt ssRNase +Protein Rep 1
Salt ssRNase -Protein Rep 1
Salt ssRNase +Protein Rep 2
Salt ssRNase -Protein Rep 2
Salt dsRNase -Protein Rep 1
Control dsRNase -Protein Rep 1
Salt dsRNase +Protein Rep 1
Salt dsRNase +Protein Rep 2
Control dsRNase -Protein Rep 2
Control dsRNase +Protein Rep 2
Salt dsRNase -Protein Rep 2
Control dsRNase +Protein Rep 1

Supplemental Figure 2: High reproducibility and quality of PIP-seq libraries in control- and salt-treated tissue

(A-H) Correlation plots between biological replicates of PIP-seq libraries from control- (A-D) and salt-treated (E-H) tissue. Pearson correlation.

(I) Clustering of PIP-seq libraries by DESeq2.

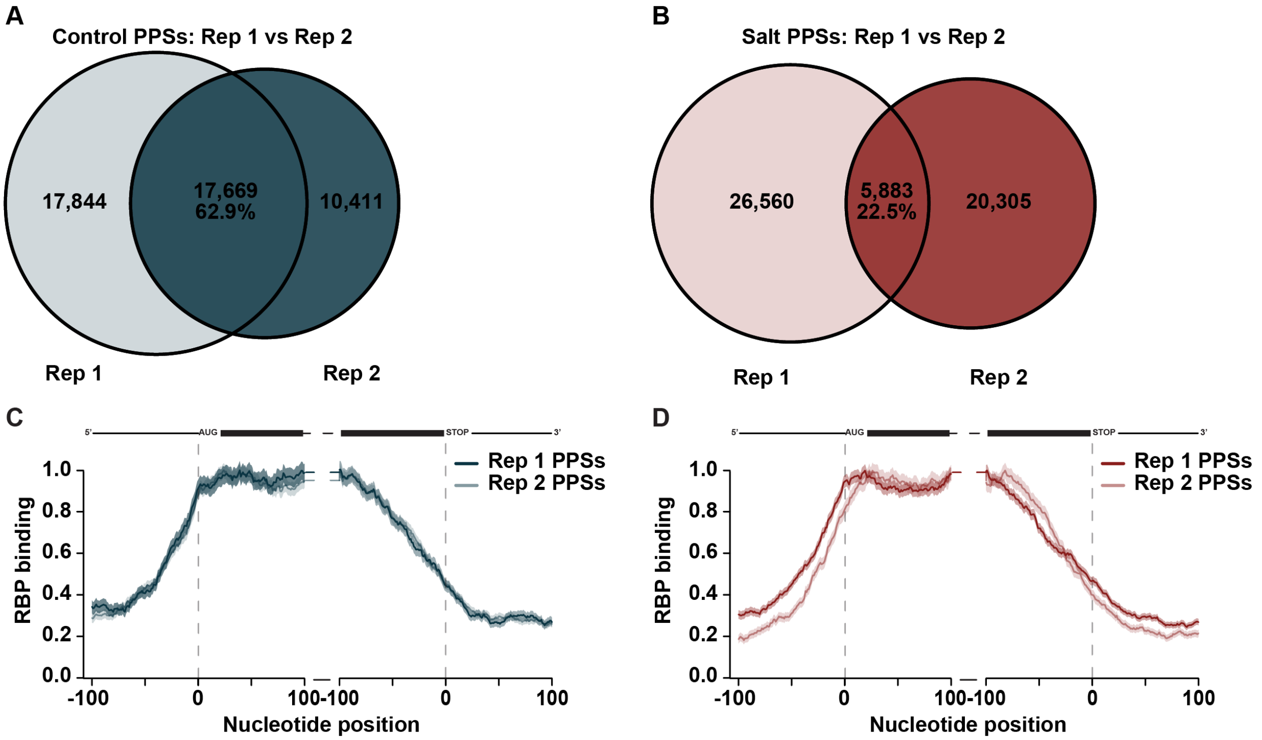

Supplemental Figure 3: Overlaps between PPSs identified in nuclear PIP-seq in control- and salt-treated tissue

(A-B) Overlap between biological replicates of control-treated (A) and salt-treated (B) tissue for all PPSs. PPSs must overlap by at least 1 nucleotide to be classified as common between the replicates.

(C-D) RBP binding density plots for all PPSs identified in each biological replicate from control- (C) and salt-treated (D) tissue. Solid line represents average RBP binding densities at each nucleotide and shading represents SEM. mRNA diagrams above plots are not to scale.

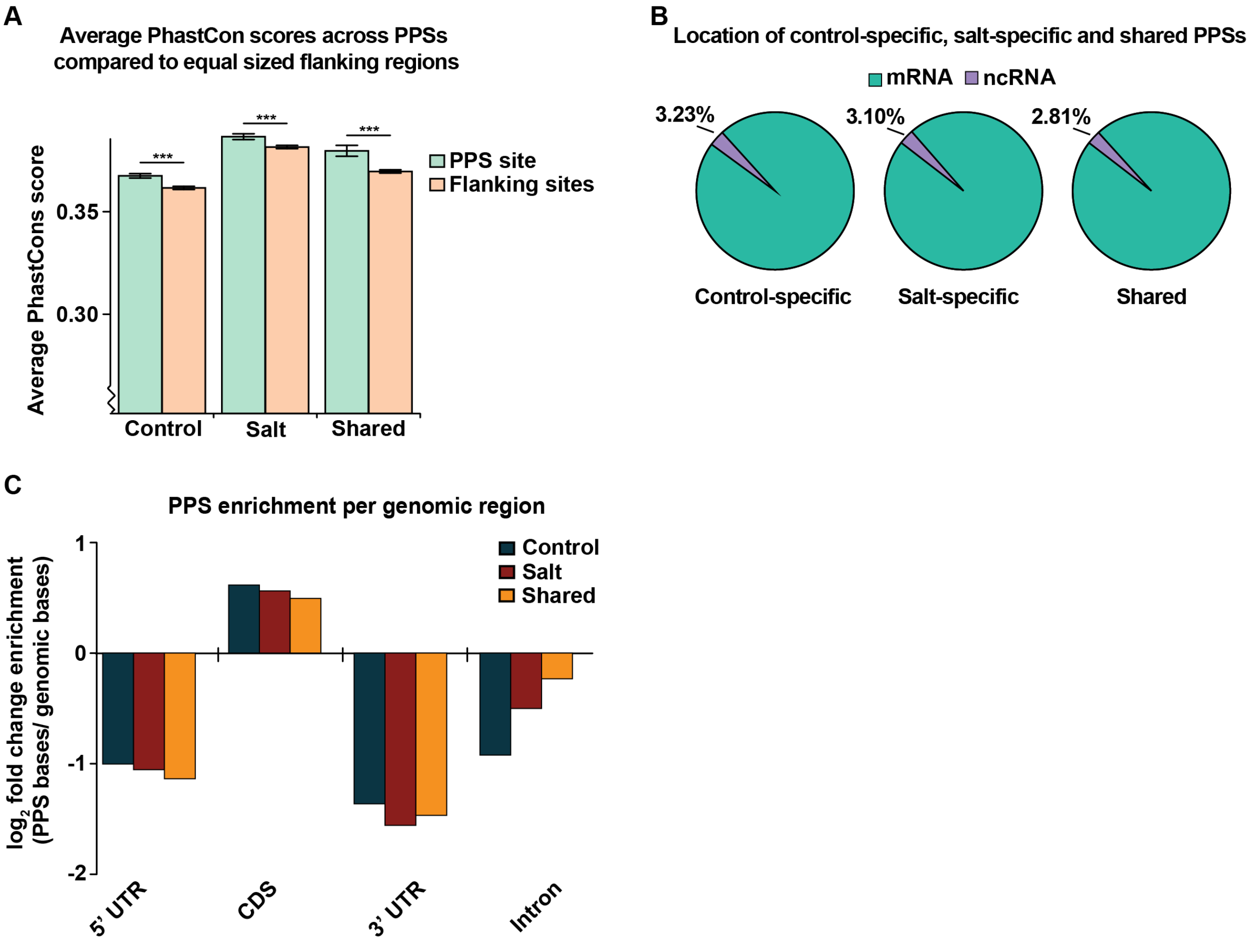

Supplemental Figure 4: Functional validation of PPSs identified in nuclear PIP-seq in control- and salt-treated tissue

(A) Average PhastCons scores for control-specific, salt-specific, and shared high-confidence PPSs (green bars) and equal sized flanking regions (peach bars). Error bars indicate SEM. *** denotes p-value < $1x10^{-10}$, Kolmogorov-Smirnov test.

(B) Percentage of control-specific, salt-specific, and shared high-confidence PPSs identified in protein-coding mRNAs and ncRNAs.

(C) PPS enrichment in 5' UTR, CDS, 3' UTR, and intron for control-specific (blue), salt-specific (red), and shared high-confidence PPSs (yellow).

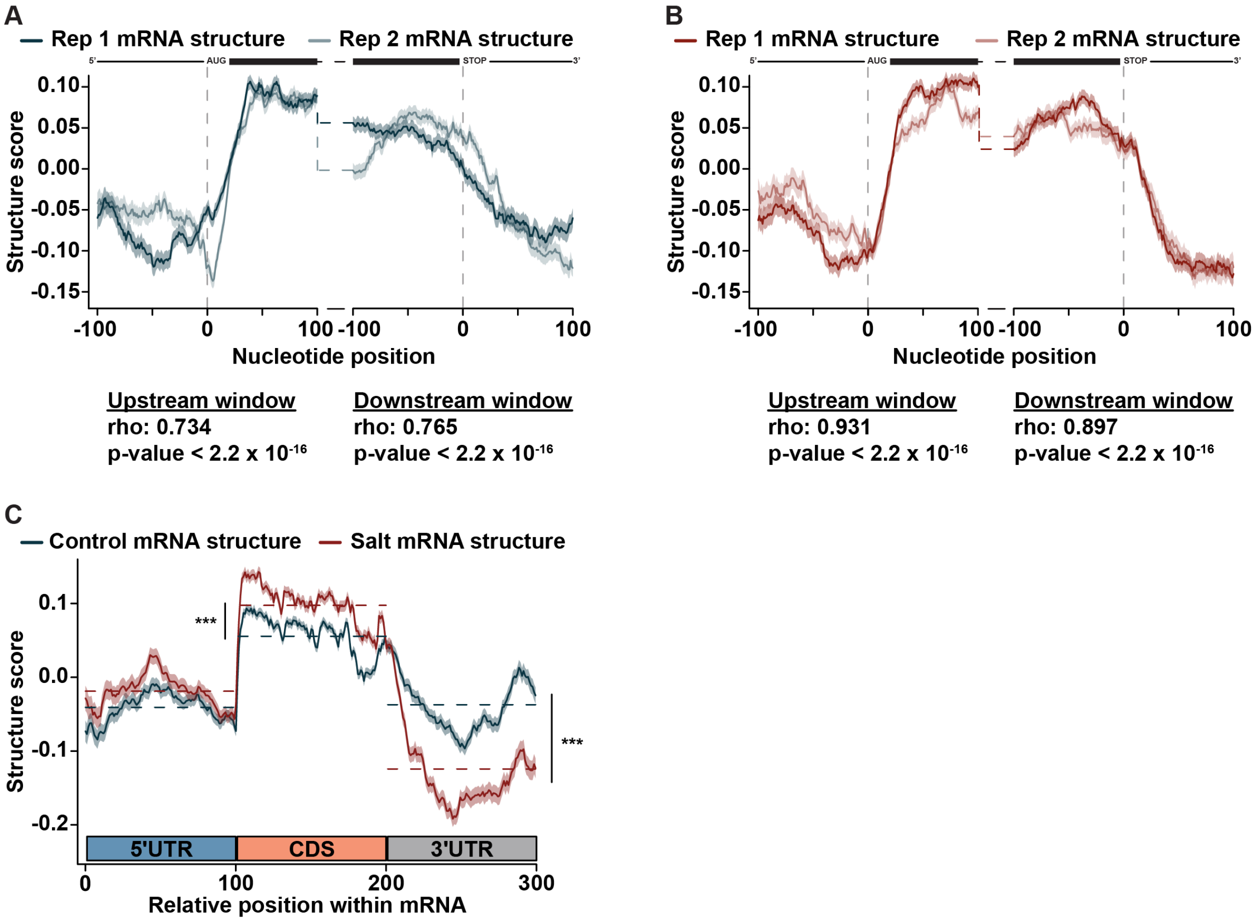

Supplemental Figure 5: RNA secondary structure is highly reproducible and changes significantly upon salt stress.

(A-B) RNA secondary structure scores at the +/- 100 nt the start and stop codon for each biological replicate from control- (A) and salt-treated (B) tissue. Solid line represents average structure scores at each nucleotide and shading represents SEM. mRNA diagrams above plots are not to scale.

(C) Average structure scores plotted over the 5’ UTR, CDS and 3’ UTR of all detectable protein-coding mRNAs in control-treated (blue) and salt-treated (red) tissue. The overall average structure score for each region is plotted as a dotted line. Shading around the line indicates the SEM across all plotted transcripts. N = 14,461 mRNAs. *** denotes p-value < 0.001, Wilcoxon test.

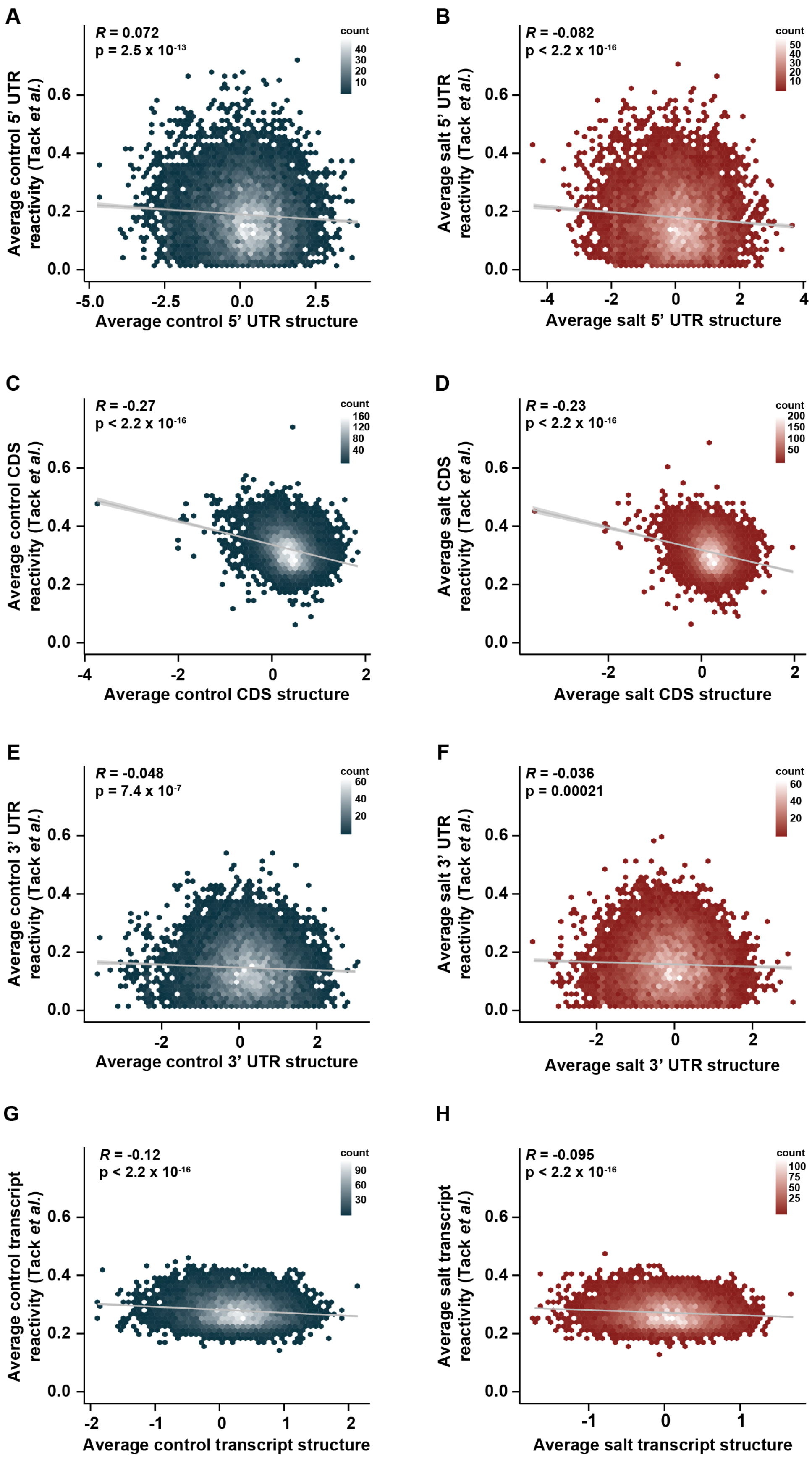
A
R = 0.072
p = 2.5 x 10^-13
Average control 5' UTR reactivity (Tack et al.)
Average control 5' UTR structure
B
R = -0.082
p < 2.2 x 10^-16
Average salt 5' UTR reactivity (Tack et al.)
Average salt 5' UTR structure
C
R = -0.27
p < 2.2 x 10^-16
Average control CDS reactivity (Tack et al.)
Average control CDS structure
D
R = -0.23
p < 2.2 x 10^-16
Average salt CDS reactivity (Tack et al.)
Average salt CDS structure
E
R = -0.048
p = 7.4 x 10^-7
Average control 3' UTR reactivity (Tack et al.)
Average control 3' UTR structure
F
R = -0.036
p = 0.00021
Average salt 3' UTR reactivity (Tack et al.)
Average salt 3' UTR structure
G
R = -0.12
p < 2.2 x 10^-16
Average control transcript reactivity (Tack et al.)
Average control transcript structure
H
R = -0.095
p < 2.2 x 10^-16
Average salt transcript reactivity (Tack et al.)
Average salt transcript structure
count

Supplemental Figure 6: Correlations between PIP-seq derived, nuclear RNA secondary structure scores and reactivity scores from Tack *et al*.

(A-H) Comparisons between average structure scores in control- (A,C,E,G) and salt-treated (B,D,F,H) tissue in the 5’ UTR (A-B), CDS (C-D), 3’ UTR (E-F), and whole transcripts (G-H) calculated by PIP-seq and DMS-seq by Tack *et al*. Pearson correlation. Plots generated using geom_hex from ggplot2 R package with 50 bins specified. Colors indicate the number of transcripts within each bin. Grey lines indicate linear regression.

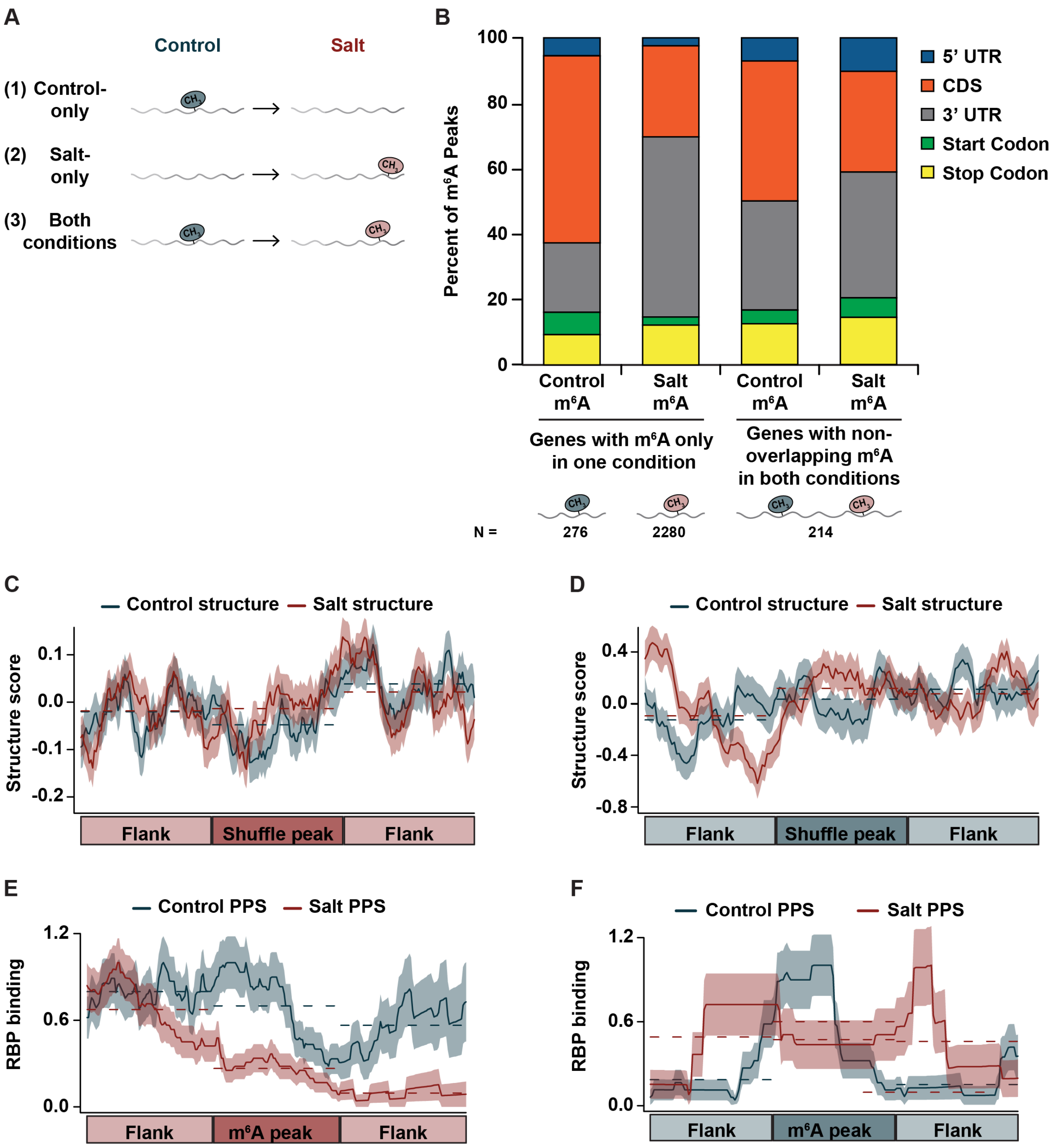

Supplemental Figure 7: RNA secondary structure and RBP binding across shuffled control-specific and salt-specific m^{6}A peaks

(A) Representative figure demonstrating three classes of m^{6}A modified transcripts. (1) Control-only transcripts that contain a high-confidence m^{6}A peak(s) in control-treated tissue, but no high-confidence m^{6}A peaks in salt-treated tissue, (2) Salt-only transcripts that contain a high-confidence m^{6}A peak(s) in salt-treated tissue, but no high-confidence m^{6}A peaks in control-treated tissue, and (3) Transcripts that contain a high-confidence m^{6}A peak in both conditions, but the peaks are independent and do no overlap.

(B) Classification of the location of m^{6}A peaks within mRNAs for genes that only have high-confidence m^{6}A peak(s) in control-treated tissue, only in salt conditions, or contain non-overlapping high-confidence m^{6}A peaks in both control- and salt-treated tissue.

(C-D) RNA secondary structure scores in control-treated (blue) and salt-treated (red) tissues across shuffled binned salt-specific (C) or control-specific (D) m^{6}A peaks as well as equal sized flanking regions. Dashed lines represent the average structure for each region. Shading around the line indicates the SEM across all detectable transcripts.

(E-F) RBP binding in control-treated (blue) and salt-treated (red) tissues across binned salt-specific (E) or control-specific (F) m^{6}A peaks located in the 3’ UTR as well as equal sized flanking regions. Dashed lines represent the average structure for each region. Shading around the line indicates the SEM across all plotted transcripts.

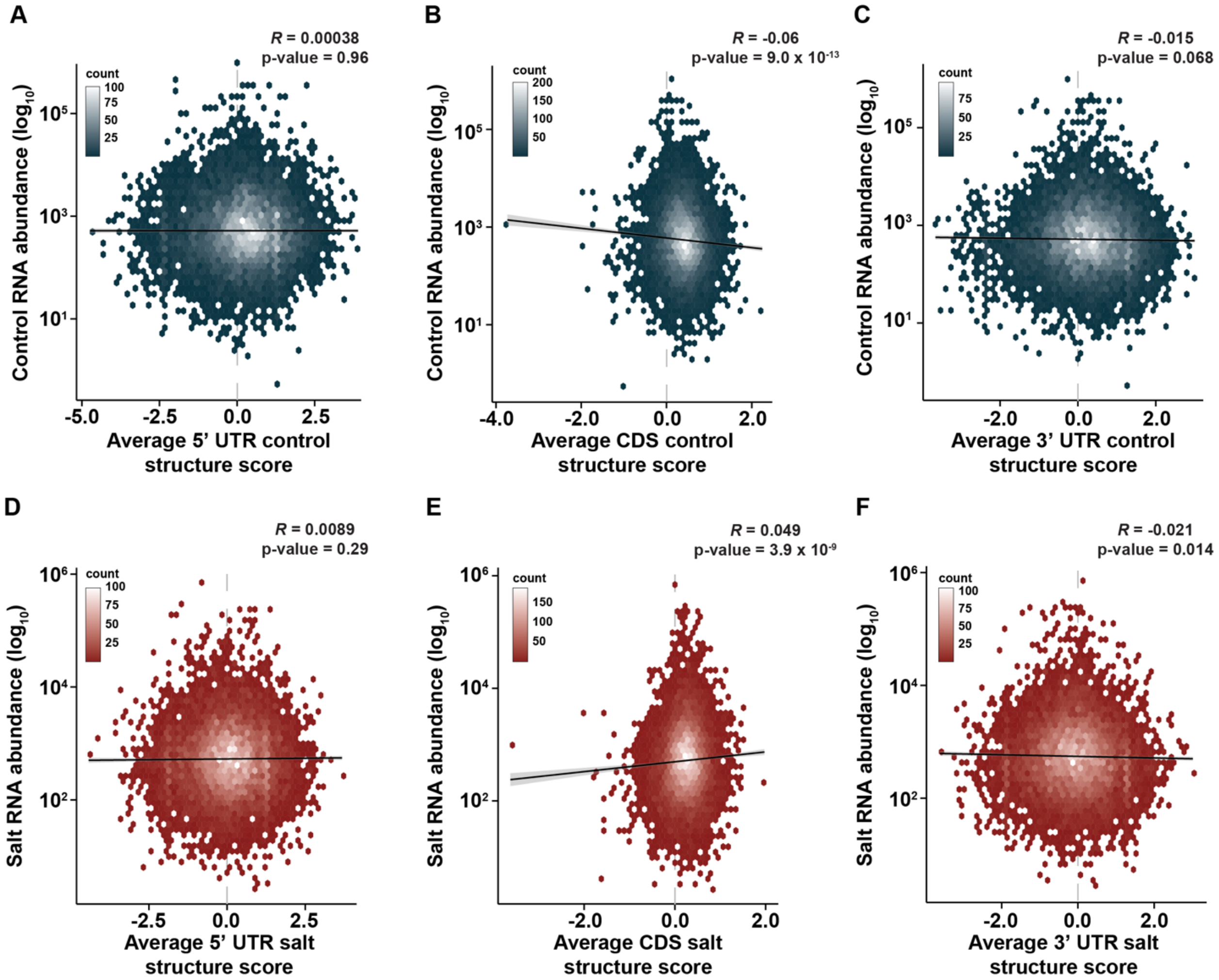

Supplemental Figure 8: Correlations between RNA secondary structure and mRNA abundance in control- and salt-treated tissue.

(A-C) Relationship between average RNA secondary structure in the 5' UTR (A), CDS (B), and 3' UTR (C) and mRNA abundance in control-treated tissue. Pearson correlation (D-F) Relationship between average RNA secondary structure in the 5' UTR (D), CDS (E), and 3' UTR (F) and mRNA abundance in salt-treated tissue. Pearson correlation.

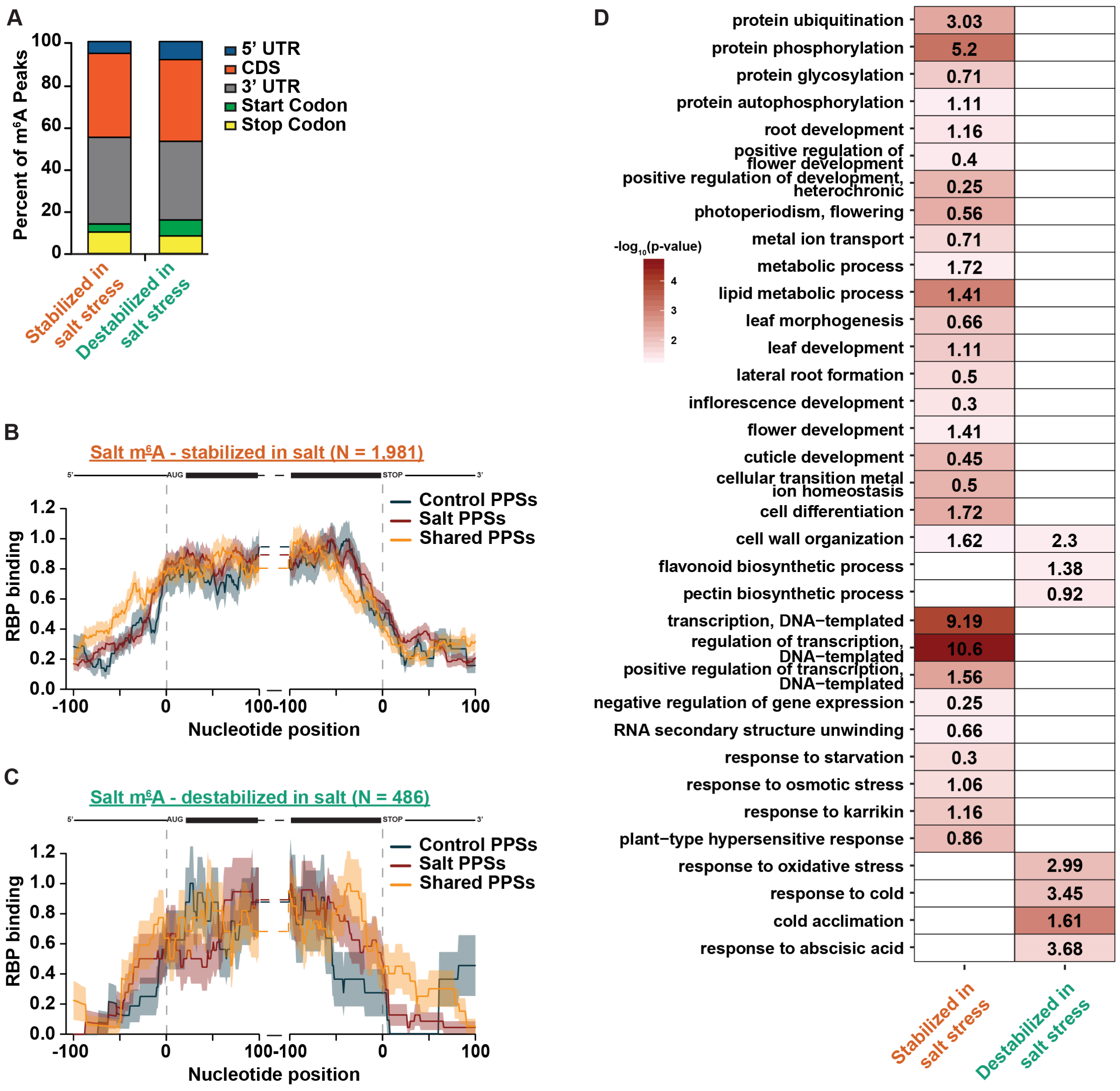

Supplemental Figure 9: Transcripts encoding proteins involved in osmotic stress response gain m^{6}A and are stabilized during systemic salt stress response

(A) Classification for salt-specific m^{6}A peaks within protein-coding mRNAs that are destabilized or stabilized during systemic salt stress response.

(B-C) RBP binding in the +/- 100 nt of the annotated start and stop codon in nuclear protein- coding mRNAs that gain m^{6}A and are stabilized (C) or destabilized (D) during salt stress response. PPSs were divided into those that were expressed exclusively in control-treated tissue (blue line), salt-treated tissue (red line) or common to both treatments (yellow line). mRNA diagrams above plots are not to scale.

(D) Gene ontology (GO) analysis using DAVID (Huang et al., 2009) for transcripts that gain m^{6}A and are destabilized or stabilized during salt stress response. Color within each cell represents the calculated -log$_{10}$(p-value), while the values denoted within each cell is the fold enrichment provided by the DAVID analysis package.

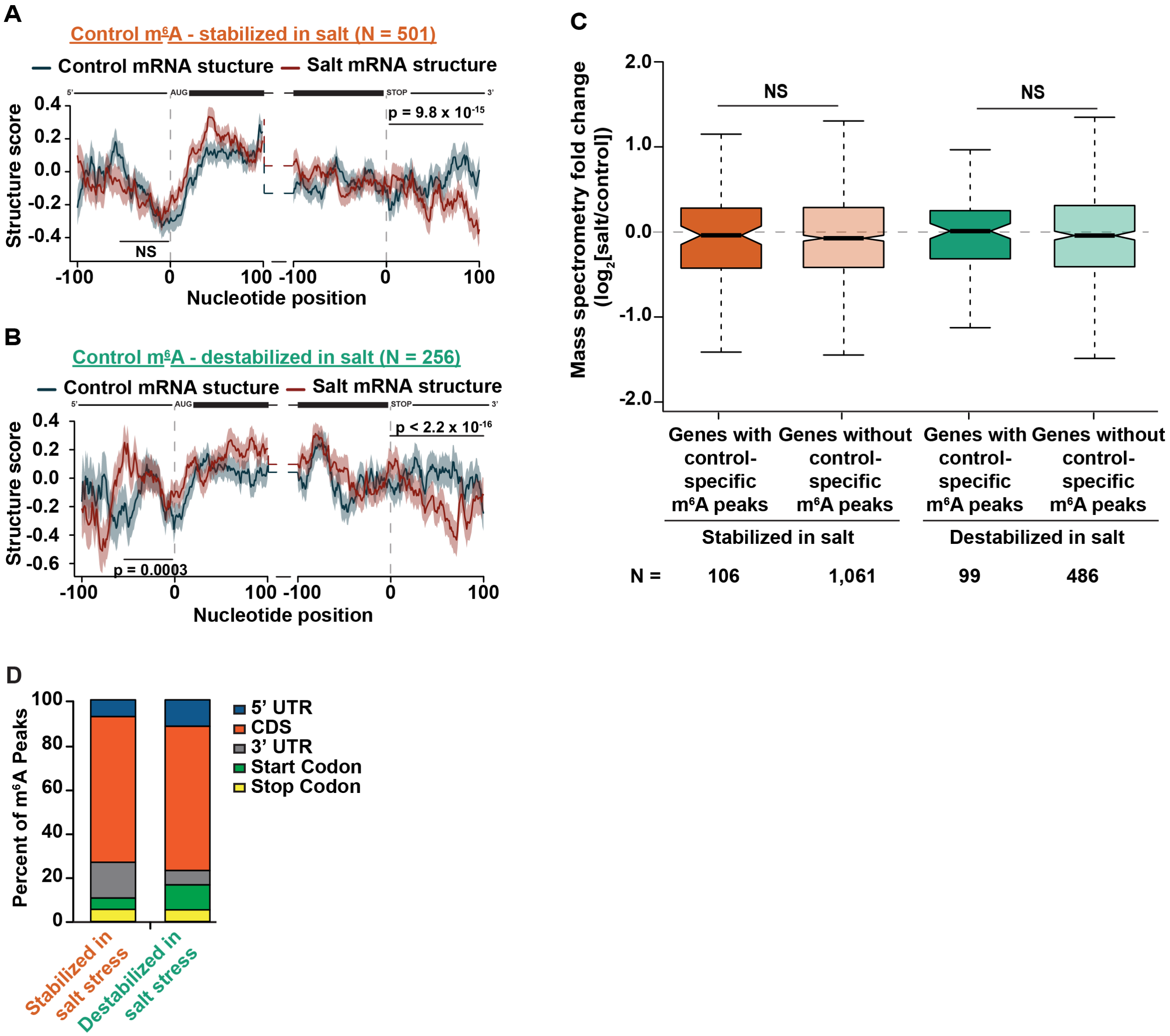

Supplemental Figure 10: mRNA stability of transcripts that have control-specific m^{6}A doesn't affect RNA secondary structure, protein abundance or m^{6}A location

(A-B) Average structure score in control-treated (blue line) and salt-treated (red line) tissue in the +/- 100 nt of the annotated start and stop codon of nuclear protein-coding transcripts that have control-specific m^{6}A and are stabilized (A) or destabilized (B) during long-term salt stress response. Shading around the line indicates the SEM across all plotted transcripts. P-values were calculated using a Wilcoxon test and are denoted over the specific regions.

(C) Protein abundance fold change (log$_2$[salt/control]) for transcripts that contain control-specific m^{6}A peaks (darker colors) or lack control-specific m^{6}A peaks (lighter colors) and are stabilized (orange) or destabilized (green) during salt stress response. NS denote p-value > 0.05, Wilcoxon test.

(D) Classification for control-specific m^{6}A peaks within protein-coding mRNAs that are destabilized or stabilized during systemic salt stress response.

A

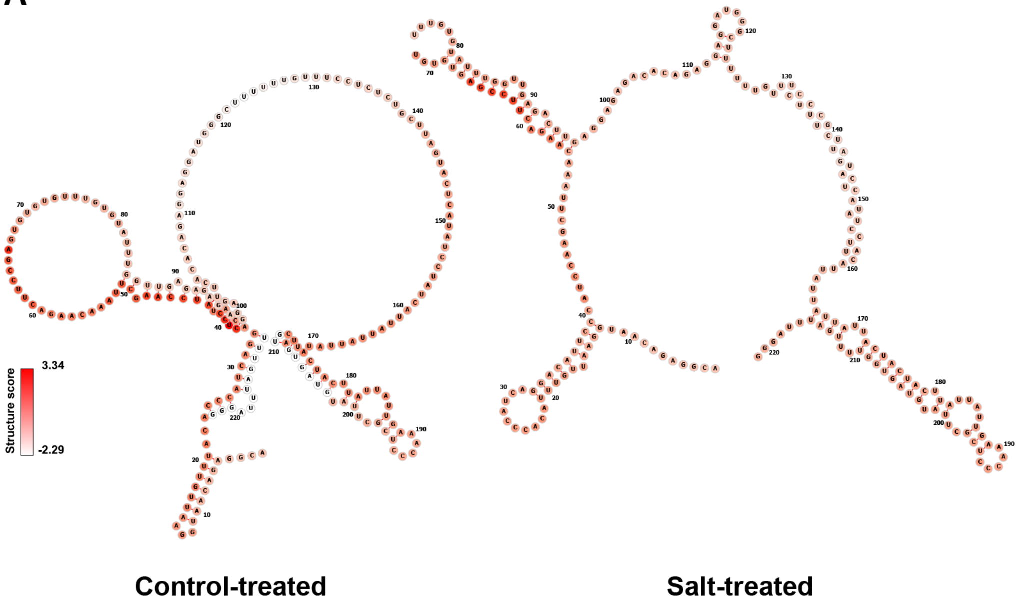

B

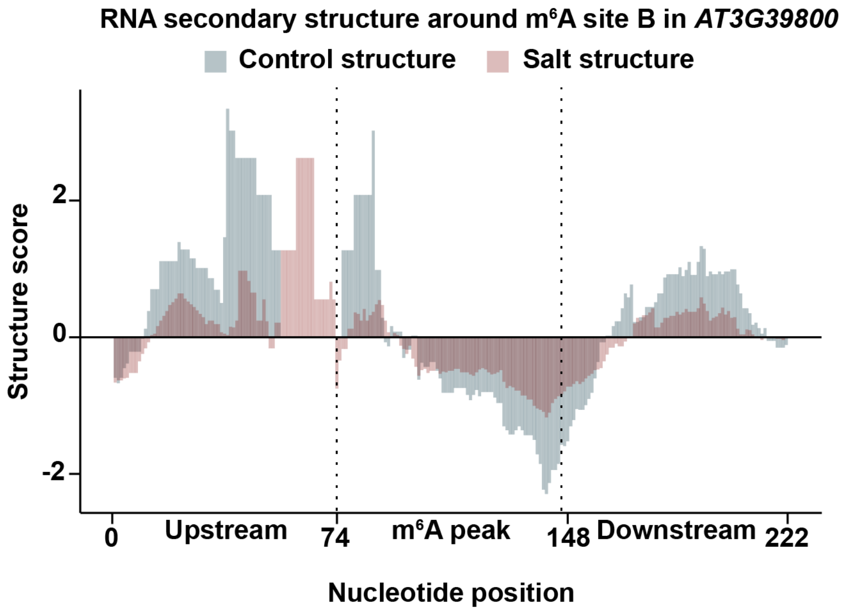

Supplemental Figure 11: RNA secondary structure surrounding m^{6}A peak B in *AT2G39800*.

(A) RNAfold model for m^{6}A peak B in *AT2G39800* in control- (left) and salt-treated (right) tissue constrained with PIP-seq determined structure scores. Color of each nucleotide indicates structure score, with darker colors indicating higher structure score.

(B) RNA structure scores from PIP-seq for peak B within *AT2G39800* and equal sized flanking regions to the 5' and 3' end.
