# Supplementary material for: N6‐methyladenosine and RNA secondary structure affect transcript stability and protein abundance during systemic salt stress in Arabidopsis
Source: Plant Direct. 2020 Jul 24;4(7):e00239. doi: 10.1002/pld3.239 (PMC7379018; doi:10.1002/pld3.239)
Supplement: Supplementary file 3 — Supplementary Material [file PLD3-4-e00239-s003.pdf]

<u>Selected comments to address by Dr. Ivan Baxter</u>

3. In Supplemental figure 2, the overlap of salt PPSs between replicates is 22.5% which is very low. Even under control condition, the PPSs between replicates is 63%. It seems like poor reproducibility between the replicates.

We confirmed the high quality and reproducibility of each library between biological replicates by examining read coverage in all libraries in 1000 nucleotide bins with a 100 nt sliding window. All libraries have Pearson's correlation (*R*) > 0.86 (new Supplemental Figures 2A-H). Additionally, the DESeq2 heat plot of read coverage in 1000 nt bins demonstrates the high reproducibility of the libraries (new Supplemental Figure 2I). We have also calculated RBP binding density for each biological replicate separately and examined the pattern of RBP binding around the start and stop codon and found virtually identical patterns of binding, indicating that, while not all PPSs are found in both biological replicates, they share the same patterns of binding (new Supplemental Figures 3C-D). We acknowledge the low overlap between salt biological replicates and given the high quality and reproducibility of the PIP-seq (RNA-seq variant) libraries between replicates (new Supplemental Figures 2E-H), this low overlap is likely due to biological variations that occur during systemic salt stress. We do note that the 62.9% overlap of PPSs between biological replicates in control-treated tissue is extremely high for a transcriptome-wide approach looking at global RBP, especially when one compares this value to the average overlap in single protein approaches such as CLIP-seq which are usually less than 45%. Regardless of the strong evidence of reproducibility of PIP-seq libraries and the expected biological variation in our PPS overlaps, we chose to perform all subsequent analyses for our revised manuscript with the high-confidence PPSs that were found in both biological replicates in both control and salt treatments (control N = 17,669; salt N = 5,883) (new Supplemental Figures 3A-B). These revised analyses using only these high-confidence PPSs did not result in any significant changes to the overall conclusions of our study.

5. Following the salt treatment (150mM NaCl), the seedlings were transferred into the nuclear purification buffer (40mM NaCl). Given the fact that Arabidopsis response to Na+ change within minutes (Yang et al., 2017), how did the authors assure the sufficient salt treatment? Why not increase the NaCl concentration in the nuclear purification buffer?

For our study, we used salt concentrations optimized and published previously (Monihan et al. 2019; Monihan et al. 2020) to study post-transcriptional regulation of long-term (systemic) salt stress. Specifically, salt concentrations were chosen for this study based on the decrease in fresh weigh determined previously (Monihan et al. 2019; Monihan et al. 2020).

All RNA-RNA and RNA-protein interactions are stably crosslinked using formaldehyde immediately after being collected from both control- and salt-

treated flats and then flash frozen in liquid N_2 and stored at -80 until nuclei isolation. Subsequently, INTACT and PIP-seq library preparation are both performed using these stably crosslinked tissues, thus the salt concentration shouldn't affect any RNA-RNA or RNA-protein interactions as they are fixed in the crosslinking process.

6. The Poisson-based test was used to identify the enriched region of footprinting signal by comparing to the structure-only signal, which did not take account for the biological variations for both footprinting signals and structure-only signals. A previous study (Choudhary et al., 2019) has demonstrated how to identify differentially reactive regions between two groups of samples based on the differential analysis accounting for biological variations. It is important to include the measurement of biological variations for both structure scores and RBP binding scores while comparing the variations between two groups (control vs salt conditions). For instance, if the authors plot the structure scores/RBP binding scores for each replicate for both control and salt conditions, then the authors could confidently identify the differentially reactive regions between control and salt conditions. In this way, the authors could avoid identifying both false positive and false negative signals using only average scores.

To address this concern, we calculated RBP binding and RNA secondary structure for each biological replicate separately (new Supplemental Figures 3C-D; new Supplemental Figures 5A-B). From these analyses, we found that both replicates produced highly similar and correlated patterns of RBP binding and structure scores, as well as similar RBP binding density values and structure scores between the biological replicates for both treatments. Based on these highly overlapping results, we conclude that the biological replicates are extremely similar, and therefore proceeded with PPSs that were found in both biological replicates and calculated structure scores for merged biological replicates in all subsequent analyses presented in the revised manuscript.

1) One fundamental approach is RNA footprinting, that is heavily dependent on a group of enzymes like proteinase K, ribonucleases (RNases) that digest single-stranded RNA (ssRNA; ssRNase--RNaseONE (?) or double stranded RNA (dsRNA; dsRNase--RNaseV1 (?). The authors did not provide detailed information for these enzymes---RNase V1 has been out of market for a while as far as this reviewer knows-if so, what did the authors use here?). The main issue is that whether enzymes work always well and equally efficiently with different samples prepared from plants with different developmental stages and plants that grow different environmental conditions?

Information about the RNases is included in the citations of two PIP-seq methods papers (Foley and Gregory, 2016; Kramer and Gregory, 2019), as well as in the other nuclear PIP-seq studies cited in this paper (Gosai et al., 2015 and Foley et al., 2017a). To address this concern, we have expanded the Methods section for PIP-seq library preparation in our revised manuscript to include the information about the RNases used as the following.

"PIP-seq libraries were constructed as previously described (Foley and Gregory, 2016; Kramer and Gregory, 2019). To summarize briefly, INTACT purified nuclei from 3 grams of tissue per replicate were lysed and separated into footprinting and structure-only samples. The footprinting samples were then treated with either dsRNase (RNaseV1; purified, tested, and validated in the Gregory lab with Protein Labs (San Diego, CA, USA) ds-P) or ssRNase (RNaseONE; Promega; Madison, WI, USA; ss-P) before protein digestion by proteinase K and reversal of crosslinks."

The RNases are acting on purified nuclei, thus the input to PIP-seq RNase digestion is essentially the same as that from nuclei isolated from 10-day-old whole seedlings as well as root hair and non-hair cells. Thus, the tissue or developmental time point should not affect the function of the enzymes since the material being used in library construction at this point is nearly identical in nature (RNA in the RNase buffer). Furthermore, similar numbers of nuclei were used as starting material for all nuclear PIP-seq studies performed in the Gregory lab (Gosai et al., 2015; Foley et al., 2017a; this study).

In fact, some results underscored this concern: Careful examination of protein protected sites (PPSs) in Supp Fig S2A and S2B does not really show the reproductivity of high-quality and specificity of the nuclear PIP-seq libraries as indicated. There are only ~22% of all PPSs in salt-treated tissue, identified in two biological replicates. Given there is a huge variation within samples with the same treatment, how could one compare the data between treatments? In addition, the authors pooled all PPSs even not reproducible, for further analysis in Fig 1A, Fig S3B, Fig 1B, Fig S3C, Fig 1C, etc, this reviewer is unable to not see how the conclusion could be drew (indicating that nuclear RBP-RNA interactions vary during salt stress response---P8, line 242).

This concern on the biological variation between replicates was addressed in comments to previous concerns described above. Briefly, we confirmed the quality and reproducibility of the PIP-seq libraries (new Supplemental Figure 2) in multiple ways, directly compared RBP binding and structure scores for each biological replicate separately (new Supplemental Figures 3C-D; new Supplemental Figures 5A-B, respectively), and to be extremely careful only examined the high-confidence PPSs found in both biological replicates (new Supplemental Figures 3A-B) for all subsequent PPS and RBP binding density analyses.

2) Another example: line 325-328 "Similar to protein binding, RNA secondary structure was higher in the CDS compared to the 5' UTR and 3' UTR. This is contrary to previous findings in the nuclei from 10-day-old whole seedlings and roots (Gosai et al., 2015; Foley et al., 2017a), signifying that RNA secondary structure may be regulated in a tissue- and/or developmental time-specific manner.

To address this concern, we have modified this statement to soften the conclusiveness of our language to the following.

> **"Similar to protein binding, RNA secondary structure scores were higher in the CDS compared to the 5' UTR and 3' UTR. This is contrary to previous findings in the nuclei from 10-day-old whole seedlings and roots (Gosai et al., 2015; Foley et al., 2017a), suggesting that RNA secondary structure may be regulated in a tissue- and/or developmental time-specific manner."**

As mentioned above, the starting material (nuclei from INTACT) was the same in both Gosai et al., 2015 and Foley et al., 2017a, thus any variations observed in our sample is likely due to biological variations.

Moreover, the authors have a totally different conclusion regarding RSS and mRNA stability, abundance, and translation product in figure 5 from the other labs. How could the authors validate and/or exclude whether the opposite patterns were simply due to the technique pitfalls?

As noted above, we do not believe that there are technical pitfalls in our PIP-seq approach and technology as it has been validated for this study and those published previously in an exhaustive and thorough manner. However, to address this concern, we have expanded on our comparisons with this study by directly comparing reactivity scores generated by Tack et al., 2020 to our PIP-seq derived structure scores for transcripts identified in control- and salt-treated tissue in both PIP-seq and their study (new Supplemental Figure 6) and added the following text to the manuscript.

> **"A previous study by Tack and colleagues examining RNA secondary structure in the total cellular RNA of shoots from 24-day-old Col-0 plants treated with short-term salt stress using a chemical-based structure probing assay to modify ssRNA found a similar trend of structural changes (Tack et al., 2020).**
>
> **To examine if RNA secondary structure is decided in the nucleus and maintained in the cytoplasm during salt stress response, we compared structure inferred by nucleotide reactivity to the chemical DMS by Tack and colleagues from whole shoot tissue treated with short-term salt stress (Tack et al., 2020) to our nuclear structure scores calculated by PIP-seq. While not overly striking, there was a significant correlation between average structure score calculated by PIP-seq (where lower scores indicated lower structure/more single-stranded) and reactivity (where higher reactivity indicated lower structure/more single-stranded) in both control- and salt-treated tissue, especially in the CDS but also in the 5' UTR, 3' UTR, and when the whole transcript was analyzed (Supplemental Figures 6A-H). These findings suggest that RNA secondary structure formed in the nucleus is a least partly maintained upon export into the cytosol."**

Be noted that nuclei compositions in seedlings and adult plants, especially salt-treated plants, are certainly different, do the enzymes work well?

As noted above, the RNases are acting on purified nuclei, thus the input to PIP-seq RNase digestion is essentially the same as that from nuclei isolated from 10-day-old whole seedlings as well as root hair and non-hair cells. Thus, the tissue, developmental time point, or treatment should not affect the function of the enzymes since the material being used in library construction at this point is nearly identical in nature (RNA in the RNase buffer). Furthermore, similar numbers of nuclei were used as starting material for all nuclear PIP-seq studies performed in the Gregory lab (Gosai et al., 2015; Foley et al., 2017a; this study).

5) Line 512 -526: The anti-correlation between m6A and RNA secondary structure does not indicate that the high abundance of m6A in these regions resulted in drastic decreases in RNA secondary structure--- this statement is not convincing without experimental evidence. The authors indicate that m6A position shift may affect RNA secondary structure at both the start codon and 3'UTR in Fig 3.

We have softened the language to acknowledge that future studies are required to examine a direct role of m^6A and RNA secondary structure.

> **"Interestingly, the regions with the highest changes in m^6A density for control-treated (3' CDS) and salt-treated (3' UTR) tissue demonstrate the largest anti-correlations, suggesting that the high density of m^6A in these regions resulted in drastic decreases in RNA secondary structure."**
>
> **"While future studies are required to confirm this, we hypothesize that this shift in m^6A density and associated widening of the dip in RNA secondary structure at the start codon in salt-treated tissue may lead to increased translation when exported into the cytoplasm."**

In Fig 4, authors used two examples to support their opinion.

In the original Figure 4, we were looking at all control- or salt-specific m^6A sites. The two examples cited here were representative images to demonstrate that the center of m^6A peaks have the highest read coverage.

Upon reexamination of the original Figure 4, we recalculated structure scores at control- and salt-specific m^6A peaks as well as equal sized regions flanking the peaks. This has now become Figures 3E-F because of the interesting and supportive nature of these results and is explained in the revised manuscript as quoted below.

> **"To do this, we took the entire length of the control- and salt-specific high-confidence m^6A peaks, divided each peak into equal sized bins and graphed the average structure score along the length of these peaks as well as equal sized regions flanking the m^6A peaks. At salt-specific m^6A**

peaks located in the 3’ UTR, there is a significant decrease in RNA secondary structure in salt-treated tissue compared to control (Figure 3E; p-value < 2.2 x 10^{-16}; Wilcoxon test). There is also a significant loss of RNA secondary structure in salt-treated tissue in the region upstream of the m^6A peak, suggesting that salt-dependent m^6A deposition causes loss of structure not only at the m^6A peak, but can also affect structure of a wider distance (Figure 3E; p-value < 2.2 x 10^{-16}; Wilcoxon test). This pattern was specific to m^6A peaks as shuffled, equal-sized control regions did not show this structural pattern (Supplemental Figures 7C-D). Additionally, this change in structure results in an overall decrease in RBP binding as compared to control conditions likely from a decrease in control-specific RBP binding events. Overall, these results suggest that an increase in RBP binding events is not the main driver of these structural changes (Supplemental Figures 7E-F).

To determine if this local change in structure was a feature common to all m^6A sites, we examined RNA secondary structure at control-specific m^6A sites located in the 3’ UTR as well. While one might expect that there would be lower structure during control conditions compared to salt stress conditions at control-specific m^6A sites, we did not see this trend (Figure 3F). This may be due to the major shift in localization of m^6A in control conditions, resulting in significantly fewer m^6A peaks located in the 3’ UTR in control conditions compared to salt (Figures 3A-B and Supplemental Figure 7B). Overall, our results suggest that salt-dependent m^6A located in the 3’ UTR can cause significant local changes in RNA secondary structure in the Arabidopsis transcriptome.”

However, there are contradictions that the authors present a change rather than a shift. Additionally, it has been reported that most mRNAs only carry one m6A modified site in plants, do these two genes contain two m6A sites at 5'UTR and 3’ UTR, or how does one m6A site shift affect two halves' RNA secondary structure?

To address this concern, we have performed additional analyses to distinguish a change in m^6A deposition from the shifting of m^6A location in response to salt treatment. These results of these analyses are presented in new Supplemental Figures 7A-B and the supporting text that we have added to the revised manuscript can be found below:

“We then took a closer look at this phenomenon by extracting transcripts that (1) contained m^6A in control-treated tissue, but lost all m^6A in during salt treatment, (2) did not contain m^6A in control-treated tissue but gained m^6A during stress, and (3) contained m^6A in both conditions, but in independent locations (Supplemental Figure 7A). m^6A located on transcripts that were m^6A modified in both conditions (Group 3) were located in the CDS and 3’ UTR in close to equal frequencies in control- and salt-treated tissues (Supplemental Figure 7B), suggesting that if a transcript is modified in both conditions, the new m^6A added during salt stress occurs in a similar transcript location (i.e. loss in 3’ UTR in control

and gain in this same region in salt) (Supplemental Figures 7A-B). However, the m^{6}A events in transcripts that completely lose this mark upon salt stress remains primarily in the CDS, while upon salt stress, previously unmodified transcripts mostly gain m^{6}A in the 3' UTR (Supplemental Figure 7B)."

7) The authors did not use any marker detect salt treated worked or not, and the negative control (like IgG) in PIP-seq or not. The brief workflow of experiment performed in this paper and the key details of should be annotated in methods.

For our study, we used salt concentrations optimized and published previously (Monihan et al., 2019, 2020). Our experimental salt concentrations were chosen based on the decrease in fresh weigh determined previously (Monihan et al., 2019, 2020). We have modified the methods to include these citations and an additional short explanation of our salt treatments for this study.

> **"Salt concentrations were optimized based on the decrease in fresh weigh determined previously (Monihan et al., 2019, 2020)."**

1. In Supplemental Figure 1C, CNX1/2 (ER marker) is clearly detectable in the nuclei from both control and salt-treated samples, which is in contrast to the author description that "we isolated highly pure nuclei enriched in the nuclear marker H3 but devoid of cytoplasmic and endoplasmic reticulum markers, PEPC and CNX1/2, respectively". Because the pure nuclei serve as the experimental basis of this manuscript, the contamination by the ER portion could largely compromise the quality of subsequent analyses.

We have modified this statement to acknowledge the presence of CNX1/2 in our nuclear samples. Additionally, we have provided representative images of DAPI stained, bead-bound nuclei after INTACT to demonstrate that the INTACT method isolated only nuclei and is absent from cellular debris, suggesting our samples our mostly nuclear with some nucleus-associated ER membrane in the samples. Therefore, we have modified the text in the revised manuscript as can be seen below.

> **"Using the INTACT system, we isolated nuclei enriched in the nuclear marker H3 but devoid of the cytoplasmic marker PEPC (Supplemental Figure 1C). It was also noticed that there were detectable levels of the endoplasmic reticulum (ER) marker, CNX1/2, in the isolated nuclei, indicating that our sample contains a majority of nuclear RNAs as well as some RNAs associated with the ER. However, the isolated nuclei were free from cytoplasmic and cellular debris, as visualized by microscopy and DAPI staining (Supplemental Figure 1D)."**

3. Figure 4 only shows the correlation between RNA secondary structure and m6A modification rather than indicating any causal link between them. The conclusion "our results demonstrate that m6A located in the 3' UTR can cause local changes in RNA secondary structure in a condition specific manner in the Arabidopsis changes in RNA

secondary structure in a condition specific manner in the Arabidopsis transcriptome" largely over-interprets the observation without solid evidence.

As mentioned in the above comment, we have reexamined this data and reinterpreted the data in new Figures 3E-F. Additionally, we have also softened the language to emphasize that this is a correlation and that additional experiments are required to directly demonstrate the function of m^{6}A in regulating RNA secondary structure. The revised text from the manuscript revisions can be seen below.

> **"To do this, we took the entire length of the control- and salt-specific high-confidence m^{6}A peaks, divided each peak into equal sized bins and graphed the average structure score along the length of these peaks as well as equal sized regions flanking the m^{6}A peaks. At salt-specific m^{6}A peaks located in the 3' UTR, there is a significant decrease in RNA secondary structure in salt-treated tissue compared to control (Figure 3E; p-value $< 2.2 \times 10^{-16}$; Wilcoxon test). There is also a significant loss of RNA secondary structure in salt-treated tissue in the region upstream of the m^{6}A peak, suggesting that salt-dependent m^{6}A deposition causes loss of structure not only at the m^{6}A peak, but can also affect structure of a wider distance (Figure 3E; p-value $< 2.2 \times 10^{-16}$; Wilcoxon test). This pattern was specific to m^{6}A peaks as shuffled, equal-sized control regions did not show this structural pattern (Supplemental Figures 7C-D). Additionally, this change in structure results in an overall decrease in RBP binding as compared to control conditions likely from a decrease in control-specific RBP binding events. Overall, these results suggest that an increase in RBP binding events is not the main driver of these structural changes (Supplemental Figures 7E-F).**
>
> **To determine if this local change in structure was a feature common to all m^{6}A sites, we examined RNA secondary structure at control-specific m^{6}A sites located in the 3' UTR as well. While one might expect that there would be lower structure during control conditions compared to salt stress conditions at control-specific m^{6}A sites, we did not see this trend (Figure 3F). This may be due to the major shift in localization of m^{6}A in control conditions, resulting in significantly fewer m^{6}A peaks located in the 3' UTR in control conditions compared to salt (Figures 3A-B and Supplemental Figure 7B). Overall, our results suggest that salt-dependent m^{6}A located in the 3' UTR can cause significant local changes in RNA secondary structure in the Arabidopsis transcriptome."**

4. In Figure 6, the authors claim that m6A deposition and the associated decrease in RNA secondary structure lead to increased mRNA stability and translation of stress related proteins. The analysis in this figure fundamentally lacks a negative control, such as genes with loss of m6A upon salt stress. The conclusion is mainly based on the analysis of the MS data and Western blot data of P5CS1, which is too preliminary.

Figure 6D,E show the selected examples, but should not be considered as a validation for the conclusion.

To address this concern, we have added the negative control of transcripts that have control-specific m^{6}A and are stabilized or destabilized during salt stress as new Supplemental Figure 10 and have added the following text to the revised manuscript to describe these new findings from this additional negative control. We note that the results are as expected, where these negative controls do not demonstrate increased protein abundance unlike the those that have salt-specific m^{6}A peaks and are stabilized.

> **"As a control, we also examined genes with or without control-specific m^{6}A peaks that are stabilized or destabilized during salt stress response (Supplemental Figure 10). Transcripts that have control-specific m^{6}A maintain the loss of RNA secondary structure in the 3' UTR during salt stress regardless of whether they are stabilized or destabilized (Supplemental Figures 10A-B). The presence of control-specific m^{6}A also does not appear to regulate protein abundance (Supplemental Figure 10C) and is still enriched in the CDS regardless of stability (Supplemental Figure 10D), suggesting that the location of m^{6}A within a transcript is essential for affecting mRNA abundance, stability, and secondary structure (Figures 3A and 3F)."**

Additionally, we have expanded the analyses of P5CS1 to include RNA abundance, mRNA stability, and RNA structure surrounding the m^{6}A peaks and included these results in a revised version of Figure 6. Finally, we have also softened the language to explain this is a hypothesis that requires future testing.

> **"To test the model that transcripts that have m^{6}A and are stabilized during salt stress indeed produce more protein, we focused on the salt stress related transcript *AT2G39800* (*DELTA 1-PYRROLINE-5-CARBOXYLATE SYNTHASE; P5CS1*). *P5CS1* encodes an enzyme that catalyzes the rate-limiting step in the biosynthesis of proline (Yoshiba et al., 1995) and is known to function during water deprivation, desiccation, and salt stress response (Feng et al., 2016; Székely et al., 2008). In fact, plants lacking P5CS1 are highly sensitive to water stress (Chen et al., 2018). Our results revealed that *P5CS1* contains two salt-specific m^{6}A peaks in its 3' UTR (Figure 6A; denoted peak A and B), increases in RNA abundance, is stabilized upon salt stress (Figure 6B) (Supplemental Data Set 9), and loses RNA secondary structure in the area surrounding its two m^{6}A peaks (Figures 6C-D and Supplemental Figures 11A-B). In western blots of protein lysates from two biological replicates, P5CS1 indeed increased ~5-fold in protein abundance in salt-treated tissue compared to control (Figure 6E), further supporting the hypothesized model that that deposition of m^{6}A, and the associated mRNA stabilization and loss of RNA secondary structure in salt stress correlates with an increase protein abundance (Figure 7)."**

The long-term salt stress treatment performed in this study may have significant indirect and secondary effects on the expression of many transcripts and proteins. There is no valid evidence in this manuscript that supports the direct role of m6A deposition and the associated decrease in RNA secondary structure in affecting mRNA stability and translation of stress related proteins.

In order to minimize indirect and secondary effects of expression on our conclusions, we enforce a read coverage filter to only examine transcripts that have at least 50 reads in PIP-seq and only examining transcripts that pass this threshold in both control- and salt-treated tissue. We agree that we have not provided direct evidence that m^6A regulates mRNA stability, structure, and translation and have attempted to soften our language throughout the manuscript to address this concern throughout our revised manuscript (highlighted in manuscript).

-- Reviewer comments:

Reviewer #1 (Comments for the Author):

The manuscript entitled, "N6-methyladenosine and RNA secondary structure affect transcript stability and translation during systemic salt stress in Arabidopsis" provides both structure and protein-protection data of nuclear RNAs in both control and salt conditions. It is interesting to explore the dynamics of structure and protein binding in response to salt stress. However, the current version is lack of both novelty and solid evidence. In general, the data analysis could be strengthened by adding better controls, determining the biological variations, using the dataset from nuclear RNAs for correlation studies and performing deeper data mining on both structure and protein protection patterns. A direct comparison with previous mature mRNA structure from DMS data in response to salt stress could bring up the novelty of this study. PIP-seq study in the essential mutants could help tackling the mechanism rather than the correlation.

My major concerns are as follows:
1. A previous study from Tack and coworkers found that the salt stress-induced RNA structure change is anti-correlated with RNA abundance (Tack et al., 2020). It will be interesting to perform a direct comparison between DMS-based structure data and RNase structure data. For instance, are the nuclear RNA structures of those photosynthesis genes similar to the mature mRNA structures? The authors might be able to observe some consistent and inconsistent global structural changes between nuclear RNAs and mature mRNAs in response to salt stress. Also, DMS modified the single-strandedness of A, which could be aligned directly with the authors' m6A data in mature mRNAs. The direct comparison with the DMS data on mature mRNAs could bring up the novelty of this study, which is currently lacking.

We thank the reviewer for this suggestion. We have directly compared reactivity from DMS-seq in Tack et al., 2020 and included this data as new Supplemental Figure 6 and addressed this in the manuscript as follows.

> **"To examine if RNA secondary structure is decided in the nucleus and maintained in the cytoplasm during salt stress response, we compared structure inferred by nucleotide reactivity to the chemical DMS by Tack and colleagues from whole shoot tissue treated with short-term salt stress (Tack et al., 2020) to our nuclear structure scores calculated by PIP-seq. While not overly striking, there was a significant correlation between average structure score calculated by PIP-seq (where lower scores indicated lower structure/more single-stranded) and reactivity (where higher reactivity indicated lower structure/more single-stranded) in both control- and salt-treated tissue, especially in the CDS but also in the 5' UTR, 3' UTR, and when the whole transcript was analyzed (Supplemental Figures 6A-H). These findings suggest that RNA secondary structure formed in the nucleus is a least partly maintained upon export into the cytosol."**

We thank the reviewer for the suggestion to examine changes in mature and nuclear RNA secondary structure by comparing DMS-seq and PIP-seq structure scores. We show that, while there is some correlation between mature and nuclear RNA secondary structure, there are definitely changes in structure between nuclear and mature RNAs. The functional significance of these change will be an interesting topic for future studies.

2. In the introduction, the authors mentioned that the motivation of performing the salt stress study is from the previous RNA binding proteomics study in Arabidopsis. Marondedze and coworkers (2016) performed their RNA binding proteomics on mature mRNAs. PIP-seq captures the binding information of RNA binding proteins in the nucleus. Given the fact that the RNA binding proteins in the nucleus are very different from those in cytosol, is it more relevant to perform PIP-seq on mature mRNAs?

We thank the reviewer for this suggestion. We have previously performed PIP-seq on total cell samples (Silverman et al., 2014; unpublished Arabidopsis study) but find that the presence of ribosomes restricts examination of PPSs of mature mRNAs. We focused on nuclear samples to avoid this restriction and to focus on co-transcriptional and nuclear post-transcriptional RBPs as processing of RNAs in the nucleus is an essential step in regulation.

3. In Supplemental figure 2, the overlap of salt PPSs between replicates is 22.5% which is very low. Even under control condition, the PPSs between replicates is 63%. It seems like poor reproducibility between the replicates.

We have addressed this concern as described above.

4. Salt is known to strongly influence the binding affinity of a protein to the RNA in general. Could the authors address that the salt treatment they performed did not

globally affect the RBP protein activity? Did the authors also observe large differences on the structure score and RBP binding in those Us RNAs and snoRNAs?

The salt stress treatment chosen by the authors is sufficient to stress the plants, but mild enough to allow for the plants to develop properly and flower. While the salt concentrations that we chose could affect RBP activity, this is likely biologically relevant and of interest to study.

We thank the Reviewer for their suggestion to examine these classes of RNAs and the authors will examine this in future analyses. The authors feel that addition of comparisons in structure of these classes of RNAs would detract from the major focus of the study presented in this manuscript.

5. Following the salt treatment (150mM NaCl), the seedlings were transferred into the nuclear purification buffer (40mM NaCl). Given the fact that Arabidopsis response to Na+ change within minutes (Yang et al., 2017), how did the authors assure the sufficient salt treatment? Why not increase the NaCl concentration in the nuclear purification buffer?

We have addressed this concern as described above.

6. The Poisson-based test was used to identify the enriched region of footprinting signal by comparing to the structure-only signal, which did not take account for the biological variations for both footprinting signals and structure-only signals. A previous study (Choudhary et al., 2019) has demonstrated how to identify differentially reactive regions between two groups of samples based on the differential analysis accounting for biological variations. It is important to include the measurement of biological variations for both structure scores and RBP binding scores while comparing the variations between two groups (control vs salt conditions). For instance, if the authors plot the structure scores/RBP binding scores for each replicate for both control and salt conditions, then the authors could confidently identify the differentially reactive regions between control and salt conditions. In this way, the authors could avoid identifying both false positive and false negative signals using only average scores.

We have addressed this concern as described above.

7. Sun and coworkers (Sun et al., 2019) found that the single-stranded region correlates with the binding affinity of m6A reader proteins. Thus, m6A sites tends to be more single-stranded. However, the authors did not find any relationship between RBP binding difference and structural change but found the m6A density change is correlated with the structural change. The authors should provide some explanation and discussion on why m6A accumulates at the sites where the m6A readers do not prefer binding.

The authors apologize for the confusion. The RBP binding density used to examine PPS abundance at m^{6}A sites can only detect if globally the number of

RNA-protein interactions change in this region but cannot determine if the identity of the protein bound changes. Thus, while the amount of PPSs at m^6A sites does not change in a condition-specific manner, the identity of the proteins binding to the PPSs very likely changes, indicating that a m^6A reader protein(s) may in fact be binding in a condition-specific manner, but they are occupying the same region as a different RBP in the opposite condition. We have added text throughout the manuscript to emphasize that RBP binding density does not equate to differences in the identity of the proteins binding.

8. It is not clear why the authors used m6A data from mature mRNAs (mainly cytosolic mRNAs, Anderson et al., 2018) to correlate with the PIP-seq data in nuclear RNAs. m6A pattern on mature mRNAs should be more associated with the structure/RBP binding patterns on mature mRNAs. Similarly, m6A pattern on nuclear RNAs should be more associated with the structure/RBP binding patterns on nuclear RNAs. Is it more relevant to perform m6A-seq on the nuclear RNAs to reflect the relationship between m6A and structure/RBP binding in the nucleus?

We thank the Reviewer for this suggestion. We performed m^6A-seq on mature mRNAs because m^6A is predicted to be deposited co-transcriptionally in the nucleus. Thus, the presence of m^6A in a mature mRNA is indicative of nuclear deposition (Sun et al. 2019). We acknowledge that by examining m^6A in mature mRNAs we may be missing some m^6A sites that have been removed by demethylases in the cytoplasm and given the differences in structure between nuclear PIP-seq calculated structure score and DMS-seq generated reactivity scores in Tack et al., 2020 (new Supplemental Figure 6), it is of great future interest to examine RNA secondary structure at m^6A sites in the cytoplasm.

9. The sequence motif for m6A is essential. The m6A sequence motif distribution across genic regions might affect the m6A density. It is important to plot the m6A sequence motif distribution across the genic regions. The authors should also generate a similar plot as Fig5c in the previous study (Sun et al., 2019) to directly show the relationship between m6A site and structure pattern. Similarly, the direct alignment of m6A site with structure patterns for individual genes in Fig 4 A and B should be used to deliver a clear message. Please refer the Fig4a in the previous study (Sun et al., 2019).

We thank the Reviewer for this suggestion. Sun et al., 2019 utilized single-nucleotide-resolution of m^6A and thus were able to determine the exact location of the modification within a transcript and in relation to the motif. Our m^6A-seq data was not generated this way and thus we cannot determine the exact locale of the modification but rather a statistical window in which the modification is likely to be, with an average peak size ~100 nts. Given the degenerative nature of the RRACH motif, each peak can contain many m^6A motifs, thus it is impossible to determine the exact location of the modification. Additionally, there are tens of thousands of instances of RRACH throughout the Arabidopsis transcriptome, only a fraction of which are modified, thus any relationship between m^6A at the RRACH motif and structure, RBP binding, or m^6A density at RRACH sites would

likely be drowned out by background noise.

10. Following my previous concerns, it is more reasonable if the authors correlate the structure data for nuclear RNAs with nascent RNA-seq data (GRO-seq data) which reflects the expression levels of nuclear RNAs. Since the nascent RNA abundance is quite different from the mature mRNA abundance, this might be the reason that the authors observed different correlations in contrast to the previous results from Tack and coworkers. This could also explain why the authors could not find the correlations between RNA structure and RNA stability/translation which were found in the previous study in mammalians (Sun et al., 2019).

We were interested in examining the effect of nuclear RNA secondary structure on RNA abundance. However, the relationship between RNA secondary structure and nascent RNA abundance is of great interest as well. We agree with the Reviewer that the different correlations in contrast to Tack and co-workers as well as the lack of relationship between RNA structure and RNA stability/translation found in Sun et al. may be due to our examination of nuclear RNA structure. In fact, given the differences in structure between nuclear PIP-seq calculated structure score and DMS-seq generated reactivity scores in Tack et al., 2020 (new Supplemental Figure 6), it is of great future interest to examine the effect of cytoplasmic RNA secondary structure on these processes. The authors only suggest in this manuscript that we do not observe any relationship between nuclear RNA secondary and RNA abundance, stability, and translation.

11. The authors previously identified the structure patterns from PIP-seq which are associated with alternative splicing which occurs in the nucleus. Is there any salt-induced structure change that is associated with the alternative splicing change?

We thank the Reviewer for this suggestion. We will examine this in future studies.

12. The authors should include both mta and xrn mutants to provide a solid mechanism to explain the correlation between m6A deposition and RNA structure. If the single-strandedness is lost in mta mutant, it could provide the causality explanation that m6A deposition leads to the single-stranded. Additionally, the authors previously discovered that m6A inhibits local ribonucleolytic cleavage to stabilize mRNAs (Anderson et al., 2018). Is there any association between structure and local ribonucleolytic cleavage?

We thank the Reviewer for this suggestion. The authors have future plans to examine RNA secondary structure in the *mta* mutant as well as examine structure at cleavage sites.

Minor concerns:
1. In Supplemental Figure 1D, the authors should provide more details on the color key and histogram in the figure legend. The clustering of RNA abundance among different libraries is quite confusing, which needs to be clarified clearly. The structure score was calculated by subtracting ssRNase-seq coverage from dsRNase coverage. The RNA

abundance between ssRNase and dsRNase should be very similar to allow the calculation of the structure score.

The color key and histogram in new Supplemental Figure 2I is defined by DESeq2 (Love et al., 2014). We have added additional correlation plots in new Supplemental Figures 2A-H to help clarify and demonstrate the high quality and reproducibility of PIP-seq libraries.

2. A comparison of RBP-binding site and structure pattern between biological replicates on some well-known RNAs is the better evidence for the reproducibility.

We have included RBP binding and structure for each replicate separately in new Supplemental Figures 3C-D and 5A-B. Additionally, we observe many PPSs within known protein-bound snoRNAs as stated here:

> **"…the next largest subset of PPSs was found to be in small nucleolar RNAs (snoRNAs) (Figure 1C), which are known to be highly protein bound, nuclear-retained small RNAs (60-200 nt long) that guide modification of nucleotides in rRNAs (Reichow et al., 2007). Thus, PIP-seq can identify RBP binding sites within ncRNAs known to be highly protein-bound as well as identify condition-specific, global RBP-RNA interaction sites throughout the plant transcriptome."**

3. In Supplemental Figure 3A, the sequence conservation varies in different genomic regions. Corresponding PhastCon scores across different genomic regions should be added.

The regions flanking the PPSs are within the same genomic regions as the PPSs, thus act as a control again variation of sequence conservation in different genomic regions. Conservation within the PPS is directly compared to that of the flanking regions. We have attempted to clarify this in the revised manuscript as can be seen below.

> **"PPSs in all three classes were significantly (p-value < $1x10^{-10}$, Kolmogorov-Smirnov test) more conserved than regions within the same genomic regions flanking the PPS (Supplemental Figure 4A)"**

4. The authors previously discovered that dynamic m6A addition stabilizes transcripts during response to salt stress. Both structure score and RBP binding depend on the RNA abundance. If the RNA abundance of one transcript shifts dramatically in salt stress in comparison with control condition, it is quite difficult to eliminate the effect of abundance change on the structure/RBP binding comparison between two conditions. Is there any coverage or abundance threshold for the data analysis?

Yes—we only examine genes that have a minimum of 50 reads in PIP-seq in both control- and salt-stress conditions.

5. Fig2A is similar to Fig2B. It could be moved to Supplemental figures.

We thank the Reviewer for this suggestion. Figure 2A has been moved to Supplemental Figure 5C.

6. In the Fig S4B in the authors' previous study (Anderson et al., 2018), the global m6A peak accumulation between control and salt conditions is very similar. However, the Fig 3B in this study showed a dramatic difference of m6A peak accumulation between control and salt conditions. The inconsistence is also observed between Fig S4C in Anderson et al., 2018 and Fig 3A in this study. The authors should provide more clarifications on these differences. Both Fig3A and 3B could move to Supplemental figures which is partially re-used in Fig 3C and 3D.

We apologize for the confusion. Figures S4B-C in Anderson et al., 2018 were high-confidence m^{6}A peaks identified in both biological replicates of control- or salt-treated tissue. In this study, we used peaks that were high-confidence control-specific, salt-specific, or shared, as stated in the manuscript as:

> **"While nearly 90% of high-confidence m^{6}A peaks identified in control-treated tissue were also identified in salt-treated tissue (shared; N=13,375), distinct classes of m^{6}A peaks were identified exclusively in control-treated tissue (control-specific; N=1,731), or in salt-treated tissue (salt-specific; N = 4,473)."**

This set of m^{6}A peaks are defined in Figure 6A in Anderson et al.

We felt that Figure 3A-B should not be moved into the supplement. We believe the comparisons between location of m^{6}A peaks demonstrated in these figures is an essential aspect of the study and findings presented in this manuscript. Specifically, these differential patterns of m^{6}A localization are key for identifying the class of transcripts that gain m^{6}A while losing structure in their 3' UTR and that ultimately increase their protein abundance in the context of systemic salt stress, which we find encode proteins that are likely important to adapting to these stressful growth conditions.

7. The Y-axis in Fig 4D should be m6A density. Similar to my major concern, the m6A sequence motif distribution should be taken account for the comparison.

This figure has been modified, and the y-axis is correctly labeled for the data currently presented.

8. The structure score and RBP binding of P5CS1 in control and salt conditions should be included.

As suggested by the Reviewer, we have included structure score surrounding the m^{6}A sites in *P5CS1* in Figures 6C-D and Supplemental Figures 11A-B in the revised manuscript.

Reviewer #2 (Comments for the Author):

RNA secondary structure (RSS) and posttranscriptional modifications such as N6-methyladensine (m6A), have been recently found to impact translational efficiency, mRNA stability, cellular localization among other events. Moreover, m6A has been reported to impact RSS by weakening intramolecular base pairing, referring there is crosstalk between the two events. Addition to this complicated regulation is that there are numerous RNA binding proteins in vivo and that the proteins might contribute to modulation of RNA secondary structure. In plants, earlier reports implicate the RNA binding proteins (RBPs) in response to osmotic or salt stress. In this context, the authors tried to fill the knowledge gap among RBP, RSS and m6A, and examine if there are correlations among them in a long-term salt stress condition. The authors treated plants with salt, cross-link with 1% formaldehyde, isolated nuclei using the INTACT method (based on biotin-attachment to nuclear envelope of specific cell types), following by protein interaction profile sequencing (PIP-seq) to identify protein bound regions on a transcriptome-wide scale and examine global patterns of RNA secondary structure during systemic salt stress response in Arabidopsis. In parallel, they mined their recently obtained m6A-seq datasets and re-purposed for this study of its relationship with RSS; and protein translation products through MS-Spec. the main conclusions included: There is a difference of RSS between control and salt stress, and the change of RSS appears not be related to RNA-RBP interaction; However, m6A is increased and its peak is shifted whereas RSS is relaxed in the 3'UTR regions of salt-responsive transcripts. Furthermore, this anti-correlation between m6A and RSS is somehow related to mRNA stability and protein translation of salt-responsive specific genes; although there is no coordination between overall patterns of RSS changes and mRNA abundance.

Overall, this is interesting work, and a very important topic in the plant field. The team has very strong expertise in computation and used in a sophisticated manner every piece of high-throughput data. They explored all possible correlations, aiming at identifying features and /or patterns. Indeed, they observed numerous features /changes in RSS, RNA binding, and m6A between control and the stress conditions that are important for plant field. However, the work also has many issues, some of which are serious, and many conclusions are rocky. First of all, discovery of co-incidence or co-relationship between two things does not mean there is cause/effect between them. Throughout the manuscripts many statements like RSS dynamics between control and salt stress, m6A causing relaxing of RSS are mostly speculation rather than conclusions. Second, one fundamental technique for this work is RNA footprinting that is heavily dependent on a group of enzymes like proteinase K, ribonucleases (RNases) that digest single-stranded RNA (ssRNA; ssRNase) or double stranded RNA (dsRNA; dsRNase--RNaseV1 which this reviewer assumed it is). The authors never experimentally validated whether these enzymes worked equally and efficiently with the nuclei samples that are isolated from different developmental stages, and plants growing different conditions. Missing critical internal and /or spiked controls would make the results very difficult for interpretation (see detailed points below). With these

limitations, I was uncertain whether the current work really represents a major breakthrough in the field.

Main concerns:

1) One fundamental approach is RNA footprinting, that is heavily dependent on a group of enzymes like proteinase K, ribonucleases (RNases) that digest single-stranded RNA (ssRNA; ssRNase--RNaseONE (?) or double stranded RNA (dsRNA; dsRNase--RNaseV1 (?). The authors did not provide detailed information for these enzymes---RNase V1 has been out of market for a while as far as this reviewer knows-if so, what did the authors use here?). The main issue is that whether enzymes work always well and equally efficiently with different samples prepared from plants with different developmental stages and plants that grow different environmental conditions? In fact, some results underscored this concern: Careful examination of protein protected sites (PPSs) in Supp Fig S2A and S2B does not really show the reproductivity of high-quality and specificity of the nuclear PIP-seq libraries as indicated. There are only ~22% of all PPSs in salt-treated tissue, identified in two biological replicates. Given there is a huge variation within samples with the same treatment, how could one compare the data between treatments? In addition, the authors pooled all PPSs even not reproducible, for further analysis in Fig 1A, Fig S3B, Fig 1B, Fig S3C, Fig 1C, etc, this reviewer is unable to not see how the conclusion could be drew (indicating that nuclear RBP-RNA interactions vary during salt stress response---P8, line 242).

We have addressed this concern as described above.

2) Another example: line 325-328 "Similar to protein binding, RNA secondary structure was higher in the CDS compared to the 5' UTR and 3' UTR. This is contrary to previous findings in the nuclei from 10-day-old whole seedlings and roots (Gosai et al., 2015; Foley et al., 2017a), signifying that RNA secondary structure may be regulated in a tissue- and/or developmental time-specific manner. Moreover, the authors have a totally different conclusion regarding RSS and mRNA stability, abundance, and translation product in figure 5 from the other labs. How could the authors validate and/or exclude whether the opposite patterns were simply due to the technique pitfalls? Be noted that nuclei compositions in seedlings and adult plants, especially salt-treated plants, are certainly different, do the enzymes work well?

We have addressed this concern as described above.

3) Line 416: "Overall, RNA secondary structure is highly dynamic during salt stress response"--- where does this conclusion come from? The presence of difference in RNA secondary structure in control vs salt-treated samples does not mean that one is static whereas the other is dynamic.

We have changed the language here to demonstrate that secondary structure shows significant changes, but not necessarily dynamic. The revised text can be seen below.

“Overall, RNA secondary structure significantly changes during salt stress response.”

4) Line 373--RNA secondary structure is a feature that can be used for unambiguous categorization of protein-coding transcripts and lncRNAs--- the huge variation of structure and RNA binding with lncRNAs might result from the low recovery reads (as written in method part--- only 5 reads for lncRNAs, but minimum 50 reads in mRNA).

We have softened the language as follows.

“Whereas there were distinct patterns of RNA structure and RBP binding at the start and stop codon of protein-coding transcripts, lncRNAs lacked any notable pattern, suggesting that RNA secondary structure is a feature that can be used for categorization of protein-coding transcripts and lncRNAs.”

Given that lncRNAs are generally less abundant than mRNAs, we had to use a lower cutoff of 5 reads to be able to examine structure and RBP binding. In our analyses, even when we look at RBP binding for lowly abundant transcripts, we still see distinct patterns of structure and RBP binding, thus it is unlikely that this is due merely to sequencing read coverage.

5) Line 512 -526: The anti-correlation between m6A and RNA secondary structure does not indicate that the high abundance of m6A in these regions resulted in drastic decreases in RNA secondary structure--- this statement is not convincing without experimental evidence. The authors indicate that m6A position shift may affect RNA secondary structure at both the start codon and 3'UTR in Fig 3. In Fig 4, authors used two examples to support their opinion. However, there are contradictions that the authors present a change rather than a shift. Additionally, it has been reported that most mRNAs only carry one m6A modified site in plants, do these two genes contain two m6A sites at 5'UTR and 3 UTR, or how does one m6A site shift affect two halves' RNA secondary structure?

We have addressed this concern as described above.

6) Line 626-641: the authors used mass spectrometry of protein lysates to refer to the translational efficiency--- This might not be correct as what the MS/Spec measures is the steady-state protein accumulation. Ribosome profiling datasets are better resources to test their models.

We thank the Reviewer for this suggestion and agree. To address this point, we have changed our language throughout the manuscript to acknowledge the possibility that the changes in protein abundance we observe may be due to changes in protein degradation. We will be performing ribosome profiling experiments to further test if the changes we observe are due to changes in translation or degradation as part of our future research directions.

Others:

7) The authors did not use any marker detect salt treated worked or not, and the negative control (like IgG) in PIP-seq or not. The brief workflow of experiment performed in this paper and the key details of should be annotated in methods.

We have addressed this concern as described above.

8) P254: Regarding PhastCon scores cross PPSs in control, salt and shared regions, the score is significantly higher in the shared regions-referring to housekeeping' RBP binding sites that are required for general molecular function-would it be better to conduct GO analysis to see if this indeed is true. Related to this question is Figure 2C and the description in Line 431-433) ", This suggests that RBPs that bind in the 5' UTR are important to regulate constitutive processes occurring in both control and salt conditions". Did the author mine the data to examine if they are indeed related to constitutive processes?

We have removed this from the manuscript, as reanalysis of high-confidence PPSs did not support this claim.

9) P331-334: In agreement with numerous studies of RNA secondary structure across multiple organisms (Ding et al., 2014b; Gosai et al., 2015; Foley et al., 2017a; Li et al., 2012b, there was a dip in RNA secondary structure directly over the start codon in both control- and salt-treated tissue---in fact, the dip patterns are opposite in this ms vs the reports as far as this reviewer recalled.

The authors are unsure of what the Reviewer referring to in this comment as the dip patterns being opposite. The decrease in secondary structure over the start codon is in the same location for all cited studies from our lab as well as others.

10) Line 405-408, While patterns of structure scores were overall similar in control- and salt-treated tissue, the dip in structure around the start codon in salt-treated tissue was broader and less pronounced than that found in control-treated tissue (p-value < 1.86 x 10-9; 407 Wilcoxon test). This indicates that, during salt stress, a larger region upstream of the start codon is alleviated of secondary structure--- this reviewer does not really see the conclusion here.

We apologize for the confusion. The lower structure upstream of the start codon is emphasized by the statistical bar and is now highlighted in light grey.

11) Line 410-411, further suggest that during salt stress, RNA secondary structure is determined in the nucleus and maintained in the cytoplasm---did the author measure RNA secondary structure from the cytoplasm?

We have added a direct comparison between our nuclear PIP-seq and DMS-seq from Tack et al. 2020 in Supplemental Figure 6. We have also modified this statement based on these results to the following in the revised manuscript.

> **"These findings suggest that RNA secondary structure formed in the nucleus is a least partly maintained upon export into the cytosol."**

12) Figure 2D is duplicate of Figure 1D?

We are confused by this comment. The figures are distinct from one another as noted in the Figure Legends.

13) Line 499, again and Line 542: m6A deposition is indeed dynamic during systemic salt stress--- the difference does not imply dynamics---

We have included additional analyses to address this point and they are described in the revised manuscript as follows.

> **"We then took a closer look at this phenomenon by extracting transcripts that (1) contained m^6A in control-treated tissue, but lost all m^6A in during salt treatment, (2) did not contain m^6A in control-treated tissue but gained m^6A during stress, and (3) contained m^6A in both conditions, but in independent locations (Supplemental Figure 7A). m^6A located on transcripts that were m^6A modified in both conditions (Group 3) were located in the CDS and 3' UTR in close to equal frequencies in control- and salt-treated tissues (Supplemental Figure 7B), suggesting that if a transcript is modified in both conditions, the new m^6A added during salt stress occurs in a similar transcript location (i.e. loss in 3' UTR in control and gain in this same region in salt) (Supplemental Figures 7A-B). However, the m^6A events in transcripts that completely lose this mark upon salt stress remains primarily in the CDS, while upon salt stress, previously unmodified transcripts mostly gain m^6A in the 3' UTR (Supplemental Figure 7B)."**

14) Line 722: mRNA of P5CS1?

We have included mRNA abundance of *P5CS1* in Figure 6B of the revised manuscript.

Reviewer #3 (Comments for the Author):

In this manuscript, the authors studied the effect of m6A on affecting RNA secondary structure and their combinatorial interplay in response to salt stress. They first studied RNA-protein interactions and RNA secondary structure at a global level in response to stress treatment, and found that RNA secondary structure is independent of changes in RNA-protein interactions. They further showed an anti-correlation between RNA secondary structure and m6A RNA methylation. Finally, the authors showed that m6A

deposition and the associated decrease in mRNA secondary structure may lead to increased mRNA stability and translation for some stress-related proteins. Although the topic discussed in this manuscript is of interest to a broad audience of plant biologists, the overall findings of this manuscript fall short of the quality and novelty for Plant Cell.

Major concerns:
1. In Supplemental Figure 1C, CNX1/2 (ER marker) is clearly detectable in the nuclei from both control and salt-treated samples, which is in contrast to the author description that "we isolated highly pure nuclei enriched in the nuclear marker H3 but devoid of cytoplasmic and endoplasmic reticulum markers, PEPC and CNX1/2, respectively". Because the pure nuclei serve as the experimental basis of this manuscript, the contamination by the ER portion could largely compromise the quality of subsequent analyses.

We have addressed this concern as described above.

2. In the PIP-seq of salt-treated tissues (Supplemental Figure 2B), the overlap between two replicates is only 22.5%, strongly arguing against the reliability and robustness of the PIP-seq experiments.

We have addressed this concern as described above.

3. Figure 4 only shows the correlation between RNA secondary structure and m6A modification rather than indicating any causal link between them. The conclusion "our results demonstrate that m6A located in the 3' UTR can cause local changes in RNA secondary structure in a condition specific manner in the Arabidopsis changes in RNA secondary structure in a condition specific manner in the Arabidopsis transcriptome" largely over-interprets the observation without solid evidence.

We have addressed this concern as described above.

4. In Figure 6, the authors claim that m6A deposition and the associated decrease in RNA secondary structure lead to increased mRNA stability and translation of stress related proteins. The analysis in this figure fundamentally lacks a negative control, such as genes with loss of m6A upon salt stress. The conclusion is mainly based on the analysis of the MS data and Western blot data of P5CS1, which is too preliminary. Figure 6D,E show the selected examples, but should not be considered as a validation for the conclusion. The long-term salt stress treatment performed in this study may have significant indirect and secondary effects on the expression of many transcripts and proteins. There is no valid evidence in this manuscript that supports the direct role of m6A deposition and the associated decrease in RNA secondary structure in affecting mRNA stability and translation of stress related proteins.

We have addressed this concern as described above.

5. The novelty of this study is also partially compromised by a recent paper (RNA 26:

492-511, 2020, Tissue-specific changes in the RNA structurome mediate salinity response in Arabidopsis), in which the authors reported the association of dynamic changes in RNA secondary structure with mRNA abundance under salt stress in Arabidopsis. In addition, the proposed mechanism of the stress-specific m6A modification in stabilizing stress response protein transcripts has been revealed in their previous study (Cell Rep. 25: 1146-1157.e3, 2018, N6-Methyladenosine Inhibits Local Ribonucleolytic Cleavage to Stabilize mRNAs in Arabidopsis), which also weakens the novelty of this study.

The authors do not agree with this comment from the Reviewer. We believe that the novelty in this study and corresponding manuscript is within the structural changes observed for nuclear structure during salt stress, the relationship between m^6A and nuclear RNA secondary structure and the evidence that mRNAs that are stabilized with m^6A during salt stress produce more protein, all of which have not been demonstrated previously.

Minor issues:
1. In Supplemental Figure 3A, I do not see any data supporting that "these shared PPSs occur in highly conserved regions of the transcriptome"

We have removed this from the manuscript, as reanalysis of high-confidence PPSs did not support this claim.

and "the importance and evolutionary pressure to retain these shared sequences".

We have added an additional statement to support this claim in the revised manuscript as can be seen below.

> **"Since RBPs tend to bind in a sequence dependent manner, there is likely evolutionary pressure to retain the sequences of these sites."**

Also, there is no data showing that "This high protein binding in the CDS is likely indicative of the importance to maintain and protect the CDS from external factors, aid in co-transcriptional processes such as mRNA splicing, and to ultimately help direct export into the cytoplasm."

We have modified this statement to the following in the revised manuscript.

> **"Thus, the high protein binding in the CDS appears to be an inherent quality of nuclear mRNAs in Arabidopsis. This high protein binding in the CDS may be indicative of the importance to maintain and protect the CDS from external factors, aid in co-transcriptional processes such as mRNA splicing, and ultimately help direct export into the cytoplasm, but additional studies are needed to test this hypothesis."**

2. "PPSs common to both conditions were significantly...... indicating that these PPSs may be a set of 'housekeeping' RBP binding sites that are required for general

molecular function". The authors could not conclude "housekeeping" through only comparing two conditions.

We have removed this from the manuscript, as reanalysis of high-confidence PPSs did not support this claim

3. It seems that the authors want to compare the number of single-stranded or double-stranded RNA. However, the claim that "RNA secondary structure was higher in...." is confusing because the structure could not be higher or lower.

We apologize for the confusion. We have modified this to: “RNA secondary structure <u>scores</u> were higher”

4. Are transcript levels of P5CS1 changed in salt-stress samples?

We have included this information in new Figure 6B in the revised manuscript.
